# Supplementary material for: Total Synthesis and Late‐Stage C−H Oxidations of ent‐Trachylobane Natural Products
Source: Angew Chem Weinheim Bergstr Ger. 2021 Nov 27;134(3):e202113829. doi: 10.1002/ange.202113829 (PMC10947344; doi:10.1002/ange.202113829)
Supplement: Supplementary file 3 — Supporting Information [file ANGE-134-0-s002.pdf]

Angewandte Chemie
Eine Zeitschrift der Gesellschaft Deutscher Chemiker

Supporting Information

Total Synthesis and Late-Stage C–H Oxidations of *ent*-Trachylobane Natural Products

*L. A. Wein, K. Wurst, T. Magauer**

Content

General Experimental Details S1

Experimental Part S3

Aldehyde S-3........ S3
Phosphonium Ylide S-5........ S3
Ester S-6........ S4
Acid S-9........ S4
Methylester S-10........ S5
Alkene S-11........ S6
Acetal S-12........ S6
Ketone 9 .... S7
Decalin 10........ S8
Enone 11........ S8
α-Methyl enone S-13 .... S10
Secondary alcohol S-15 .... S11
α-Vinyl enone 12........ S12
Allylic alcohol S-16 .... S13
Triene 13........ S14
Tricyclooctene 14........ S15
ent-Trachyloban-9-en-19-oate 16........ S17
Methyl *ent*-trachyloban-19-oate 15 .... S18
11-Oxomethyl *ent*-trachylobanoate 18........ S21
11-Oxomethyl *ent*-trachylobanoate 18 and enone 20........ S21
Enone 20........ S22
11-Oxomethyl *ent*-trachylobanoate 18 and 11-hydroxymethyl *ent*-trachylobanoate 19 .... S23
11α-Hydroxymethyl *ent*-trachylobanoate 19........ S23
11-Oxomethyl *ent*-trachylobanoate 18........ S24
ent-Trachylobanoate 6 .... S27
11-Oxo *ent*-trachylobanoate 3 .... S31
Secondary alcohol 22 .... S32
ent-Trachyloban-19-ol 17 .... S33
Iodide 25........ S37
Imine 26........ S39
Imine S-18........ S40
ent-3β-Acetoxy-trachyloban-19-al 1 .... S41
ent-Trachyloban-19-iodide S-19 .... S45
ent-Trachylobane 5 .... S46

Mechanistic Proposals........ S49

Computational Studies S52

Computational Methodology S52
Cartesian Coordinates S53
C-11 Radical S-20 .... S53
Ring Opening Transition State TS-20a S54
C-16 Radical S-21 .... S55
Ring Opening Transition State TS-20b S56
C-13 Radical S-27 .... S57
C-15 Radical S-28 .... S58
Ring Opening Transition State TS-28a S59
C-13 Radical S-29 .... S60
Ring Opening Transition State TS-28b S61
C-12 Radical S-31 .... S62

NMR Spectra S64

X-Ray Data S92

Carboxylic Acid 6 .... S92
Secondary Alcohol 22 .... S93

Chiral GC Data S94

Alcohol S-7 .... S94

References S95

General Experimental Details

All reactions were carried out with magnetic stirring, and if moisture or air sensitive, under nitrogen or argon atmosphere using standard Schlenk techniques in oven-dried glassware (100 °C oven temperature). If required glassware was further dried under vacuum with a heat-gun at 650 °C. External bath thermometers were used to record all reaction temperatures. Low temperature reactions were carried out in a Dewar vessel filled with acetone/dry ice (temperature between –78 °C and 0 °C), distilled water/ice (0 °C), sodium chloride/ice (–19 °C). High temperature reactions were conducted using a heated silicon oil bath or a metal block in reaction vessels equipped with a reflux condenser or in a pressure tube. Tetrahydrofuran (THF) was dried over molecular sieve (4 Å). All other solvents were purchased from Acros Organics as 'extra dry' reagents. All other reagents were obtained from commercial sources (Sigma Aldrich, Acros, Alfa Aesar, Tokyo Chemical Industry and others) with a purity > 95% and used without further purification unless otherwise stated.

Flash column chromatography (FCC) was carried out with Merck silica gel 60 (0.040–0.063 mm). Analytical thin layer chromatography (TLC) was carried out using Merck silica gel 60 F254 glass-backed plates or Macherey-Nagel ALUGRAM® aluminum foils and visualized under UV light at 254 nm. Staining was performed with ceric ammonium molybdate (CAM) or potassium permanganate ($KMnO_4$) and subsequent heating.

NMR spectra (^1H NMR and ^{13}C NMR) were recorded in deuterated chloroform ($CDCl_3$) on a Bruker Avance Neo 400 MHz spectrometer or a Bruker Avance II 600 MHz spectrometer and are reported as follows: chemical shift δ in ppm (multiplicity, coupling constant *J* in Hz, number of protons) for ^1H NMR spectra and chemical shift δ in ppm for ^{13}C NMR spectra. Multiplicities are abbreviated as follows: s = singlet, d = doublet, t = triplet, q = quartet, p = quintet, br = broad, m = multiplet, or combinations thereof. Residual solvent peaks of $CDCl_3$ (δ_H = 7.26 ppm, δ_C = 77.16 ppm or δ_C = 77.00 ppm for comparison of synthetic and isolated natural products) were used as internal reference. NMR spectra were assigned using information ascertained from COSY, HMBC, HSQC and NOESY experiments.

High resolution mass spectra (HRMS) were recorded on a Thermo Scientific™ LTQ Orbitrap XL™ Hybrid Ion Trap-Orbitrap Mass Spectrometer at the Institute of Organic Chemistry and Center for Molecular Biosciences, University of Innsbruck.

Infrared spectra (IR) were recorded from 4000 cm^{-1} to 450 cm^{-1} on a Bruker™ ALPHA FT-IR Spectrometer from Bruker. Samples were prepared as a neat film or a film by evaporation of a solution in $CDCl_3$. IR data in frequency of absorption (cm^{-1}) is reported as follows: w = weak, m = medium, s = strong, br = broad or combinations thereof.

Melting Points were measured with a SRS MPA120 EZ-Melt Melting Point Apparatus in open glass capillaries and are uncorrected.

Optical rotation values were recorded on a Schmidt+Haensch UniPol L1000 Peltier polarimeter. The specific rotation is calculated as follows: $[\alpha]_{\lambda}^{T} = \frac{\alpha \times 100}{c \times d}$. Thereby, the wavelength λ is reported in nm and the measuring temperature in °C. α represents the recorded optical rotation, *c* the concentration of the analyte in 10 mg/mL and *d* the length of the cuvette in dm. Thus, the specific rotation is given in $10^{-1}\cdot deg\cdot cm^{2}\ g^{-1}$. Use of the sodium *D* line (λ = 589 nm) is indicated by *D* instead of the wavelength in nm. The sample concentration as well as the solvent is reported in the relevant section of the experimental part.

X-ray diffraction analysis was carried out by Prof. Dr. Klaus Wurst at the Institute of Inorganic and Theoretical Chemistry and Center for Molecular Biosciences and Prof. Dr. Volker Kahlenberg at the Institute of Mineralogy and Petrograpgy, University of Innsbruck. The data collections were performed on a Bruker D8Quest using MoKα-radiation (λ = 0.71073 Å, Incoatec Microfocus) or on a Rigaku Oxford Diffraction CCD Gemini Ultra diffractometer using CuKα-radiation (λ = 1.54184 Å). The Bruker Apex III software or Rigaku Oxford Diffraction CrysAlis Pro software was applied for the integration, scaling and multi-scan absorption correction of the data. The structures were solved by direct methods with SHELXTL-XT-2014 and refined by least-squares methods against F2 with SHELXL-2014/7. All non-hydrogen atoms were refined anisotropically. The hydrogen atoms were placed in ideal geometry riding on their parent atoms. Further details are summarized in the tables at the corresponding sections. Plotting of thermal ellipsoids in this document and in the main text was carried out using MERCURY for Windows.

All yields are isolated, unless otherwise specified.

Experimental Part

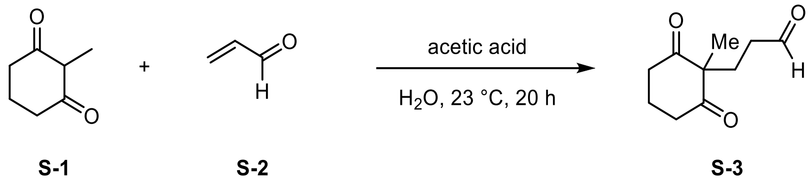

Aldehyde S-3. To a suspension of 2-methyl-1,3-cyclohexanedione **S-1** (23.3 g, 184 mmol, 1 equiv) in water (220 mL) was added acrolein **S-2** (18.5 mL, 278 mmol, 1.50 equiv), which was freshly distilled over anhydrous calcium sulfate. The reaction flask was wrapped in foil to exclude light. Acetic acid (525 µL, 9.20 mmol, 5.00 mol%) was added dropwise at 23 °C and the reaction mixture was stirred vigorously. After 20 h, the aqueous reaction mixture was extracted with ethyl acetate (3 × 120 mL). The combined organic layers were washed with saturated aqueous sodium chloride solution (120 mL) and the washed solution was dried over magnesium sulfate. The dried solution was filtered and the filtrate was concentrated to yield aldehyde **S-3** (33.5 g, 184 mmol) as a yellow oil, which was used in the next step without further purification. The analytical data were in full agreement with the literature.[1]

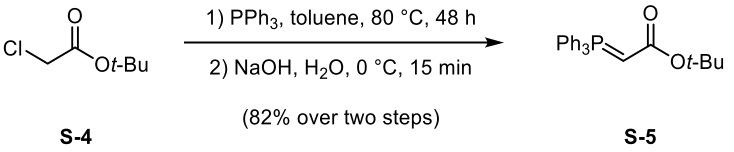

Phosphonium Ylide S-5. A suspension of *tert*-butylchloroacetate **S-4** (24.3 g, 161 mmol, 1 equiv) and triphenylphosphine (46.5 g, 177 mmol, 1.10 equiv) in toluene (200 mL) was heated to 80 °C and stirred vigorously for 48 h. After cooling to 23 °C, the obtained precipitate was filtered and the residue was washed with toluene (200 mL). The obtained white Wittig salt was dissolved in water (1 L) and aqueous sodium hydroxide solution (0.885 M, 200 mL, 177 mmol, 1.10 equiv) was added dropwise over 15 min. The precipitate was filtrated and the solid residue was washed with water (200 mL). The obtained powder was dried under high vacuum to yield phosphonium ylide **S-5** (49.5 g, 131 mmol, 82% over two steps) as a white solid, which was used in the next step without further purification. The analytical data were in full agreement with the literature.[1]

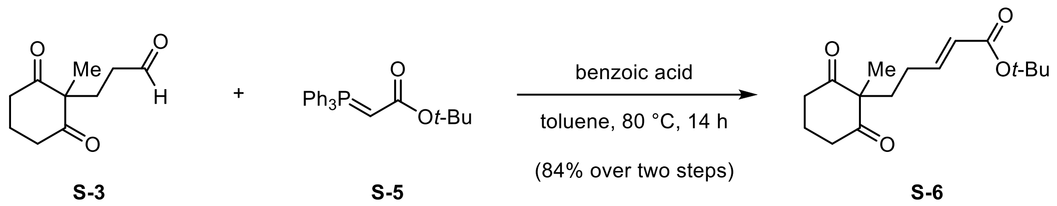

Ester S-6. A solution of aldehyde **S-3** (33.5 g, 184 mmol, 1 equiv), phosphonium ylide **S-5** (92.0 g, 245 mmol, 1.33 equiv) and benzoic acid (22.5 g, 184 mmol, 1.00 equiv) in toluene (550 mL) was heated to 80 °C and stirred for 14 h. After cooling to 23 °C, aqueous saturated sodium bicarbonate solution (400 mL) was added to the mixture and the layers were separated. The aqueous layer was extracted with ethyl acetate (3 × 400 mL). The combined organic layers were washed with saturated aqueous sodium chloride solution (300 mL) and the washed solution was dried over magnesium sulfate. The dried solution was filtered and the filtrate was concentrated. The residue was purified by flash column chromatography on silica gel (10% ethyl acetate in *n*-pentane grading to 50% ethyl acetate in *n*-pentane) to yield α,β-unsaturated ester **S-6** (43.5 g, 155 mmol, 84% over two steps) as a pale-yellow oil.The analytical data were in full agreement with the literature.[1]

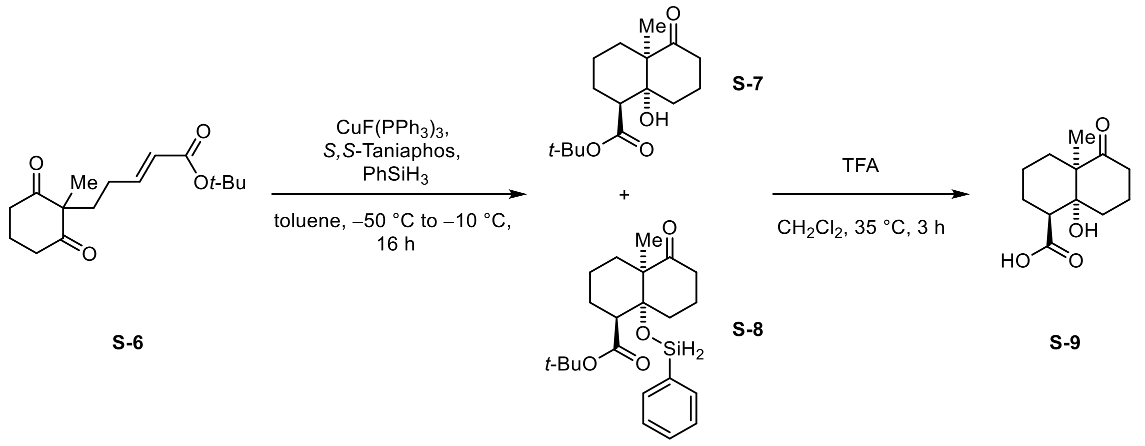

Acid S-9. A solution of fluorotris(triphenylphosphine)copper (308 mg, 0.330 mmol, 0.750 mol%) and *S,S*-Taniaphos (235 mg, 0.330 mmol, 0.750 mol%) in degassed (bubbling with argon for 30 min while sonicated) toluene (400 mL) was stirred for 30 min at 23 °C. Phenylsilane (7.60 mL, 61.6 mmol, 1.40 equiv) was added dropwise. A slight color change of the reaction mixture from pale orange to pale yellow was observed upon addition. The mixture was cooled to –50 °C and ester **S-6** (12.3 g, 44.0 mmol, 1 equiv) in degassed toluene (60 mL) was added via syringe pump over 60 min. The mixture was stirred at –50 °C for 2 h, then allowed to warm to –10 °C. After 14 h, aqueous hydrochloric acid (2 M, 500 mL) was added and the biphasic mixture was stirred for 30 min at 23 °C. The layers were separated and the aqueous layer was extracted with ethyl acetate (3 × 500 mL). The combined organic layers were washed with saturated aqueous sodium chloride solution (250 mL) and the washed

solution was dried over magnesium sulfate. The dried solution was filtered and the filtrate was concentrated to yield a mixture of crude products **S-7** and **S-8** as a brown oil. The enatiomeric excess was determined via GC-analysis of the crude material. (*vide infra*)

To a solution of crude products **S-7** and **S-8** in dichloromethane (440 mL) was added trifluoracetic acid (33.9 mL, 440 mmol, 10.0 equiv) dropwise at 23 °C. Immediate gas evolution was observed upon addition. The mixture was then heated to 35 °C and sparged with argon to remove dissolved isobutene gas. After stirring for 3 h, the mixture was cooled to 23 °C and water (300 mL) was added. The layers were separated and the aqueous layer was extracted with dichloromethane (3 × 400 mL). The combined organic layers were washed with saturated aqueous sodium chloride solution (250 mL) and dried over magnesium sulfate. The dried solution was filtered and the filtrate was concentrated. The residue was dissolved in dichloromethane (200 mL) and the solution was cooled to 0 °C. Aqueous sodium hydroxide solution (3 M, 200 mL) was added via a dropping funnel and the mixture was stirred for 10 min at 0 °C. The layers were separated and the aqueous layer was washed with dichloromethane (3 × 200 mL). Concentrated aqueous hydrochloric acid solution (37 wt.%) to the aqueous layer at 0 °C until pH = 1 was reached and the resulting mixture was stirred for 1 h at 0 °C. The aqueous layer was extracted with dichloromethane (3 × 400 mL) and the combined organic layers were dried over magnesium sulfate. The dried solution was filtered and the filtrate was concentrated to yield carboxylic acid **S-9** (9.80 g, 43.3 mmol) as a white solid, which was used in the next step without further purification. The analytical data were in full agreement with the literature.[1]

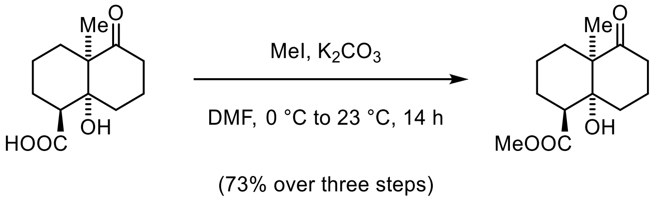

S-9 **S-10**

Methylester S-10. To a solution of carboxylic acid **S-9** (9.80 g, 43.3 mmol, 1 equiv) in *N,N*-dimethyl formamide (250 mL) was added potassium carbonate (18.0 g, 130 mmol, 3.00 equiv) in small portions and iodomethane (2.84 mL, 45.5 mmol, 1.05 equiv) dropwise at 0 °C. The mixture was warmed to 23 °C. After 14 h, saturated aqueous ammonium chloride solution (100 mL), water (100 mL) and ethyl acetate (300 mL) were added and the layers were separated. The aqueous layer was extracted with ethyl acetate (3 × 300 mL). The combined organic layers were washed with aqueous lithium chloride solution (10 wt.%, 2 × 100 mL) and saturated aqueous sodium chloride solution (100 mL) and dried over magnesium sulfate. The

dried solution was filtered and the filtrate was concentrated. The residue was purified by flash column chromatography on silica gel (14% ethyl acetate in *n*-pentane grading to 25% ethyl acetate in *n*-pentane) to yield methylester **S-10** (7.80 g, 32.0 mmol, 73% over three steps) as a colorless oil. The analytical data were in full agreement with the literature.[1]

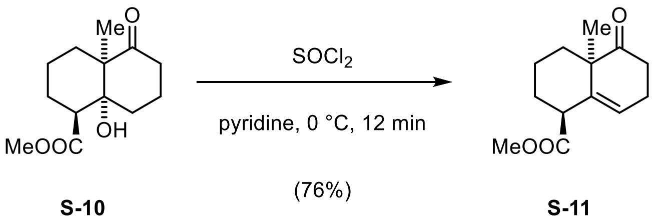

Alkene S-11. Tertiary alcohol **S-10** was divided into two equal batches, which were in parallel subjected to the reaction conditions. To each solution of tertiary alcohol **S-10** (3.88 g, 16.1 mmol, 1 equiv) in pyridine (90 mL) was added thionyl chloride (2.36 mL, 32.2 mmol, 2.00 equiv) dropwise over 2 min at 0 °C. Upon addition the color of the reaction mixture changed from pale to bright yellow. After 10 min at 0 °C, the mixture was diluted with water (90 mL). At this point the two batches were combined and the layers were separated. The aqueous layer was extracted with ethyl acetate (3 × 400 mL). The combined organic layers were washed with aqueous hydrochloric acid (1 M, 2 × 250 mL) and saturated aqueous sodium chloride solution (250 mL). The washed solution was dried over magnesium sulfate. The dried solution was filtered and the filtrate was concentrated. The residue was purified by flash column chromatography on silica gel (10% ethyl acetate in *n*-pentane) to yield olefin **S-11** (5.45 g, 24.5 mmol, 76%) as a colorless oil. The analytical data were in full agreement with the literature.[1]

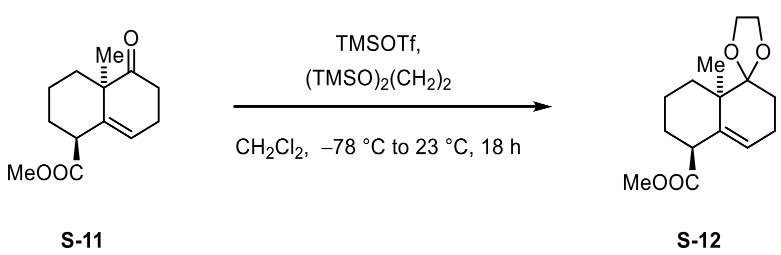

Acetal S-12. To a solution of ketone **S-11** (5.45 g, 24.5 mmol, 1 equiv) in dichloromethane (250 mL) was dropwise added freshly distilled trimethylsilyl trifluoromethyl sulfonate (222 µL, 1.23 mmol, 5.00 mol%) and ethylenedioxybis(trimethylsilane) (12.4 mL, 49.0 mmol, 2.00 equiv) successively at –78 °C. After 1 h, the reaction mixture was warmed to 23 °C. After 16 h, pyridine (2 mL) was added dropwise. Saturated aqueous sodium bicarbonate solution

(200 mL) was added and the layers were separated. The aqueous layer was extracted with dichloromethane (3 × 200 mL) and the combined organic layers were dried over magnesium sulfate. The dried solution was filtered and the filtrate was concentrated to yield acetal **S-12** (6.53 g, 24.5 mmol) as a white solid, which was used in the next step without further purification. The analytical data were in full agreement with the literature.[1]

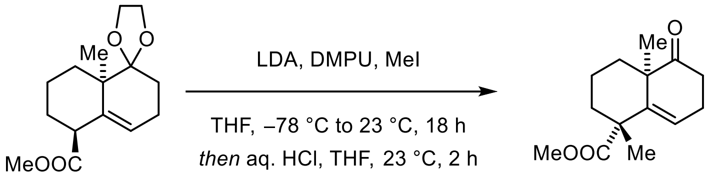

S-12 (77% over two steps) **9**

Ketone 9. To a solution of *N*,*N*′-diisopropylamine (17.5 mL, 123 mmol, 5.00 equiv) in tetrahydrofuran (280 mL) was added *n*-butyllithium (2.5 M in hexanes, 44.0 mL, 110 mmol, 4.50 equiv) via syringe pump over 20 min at –78 °C. The mixture was warmed to 0 °C. *N,N*′-dimethylpropyleneurea (29.6 mL, 245 mmol, 10.0 equiv) was added dropwise and the solution was stirred for 30 min at 0 °C. A solution of ester **S-12** (6.53 g, 24.5 mmol, 1 equiv) in tetrahydrofuran (20 mL) was added dropwise at 0 °C. After 90 min, the mixture was cooled to –78 °C and iodomethane (10.7 mL, 172 mmol, 7.00 equiv) was added dropwise, whereupon the dark red solution turned to pale yellow. The reaction mixture was allowed to warm to 23 °C. After 18 h, excess of iodomethane was removed by distillation under reduced pressure (23 °C, 200 mbar). Aqueous hydrochloric acid (6 M, 100 mL) was added and the mixture was stirred for 2 h at 23 °C. The layers were separated and the aqueous layer was extracted with ethyl acetate (3 × 400 mL). The combined organic layers were dried over magnesium sulfate. The dried solution was filtered and the filtrate was concentrated to yield ketone **9** (4.43 g, 18.7 mmol, 77% over two steps) as a white solid. The analytical data were in full agreement with the literature.[1] This sequence was repeated on similar scale twice to produce in total 11.8 g (50.1 mmol) of **9**.

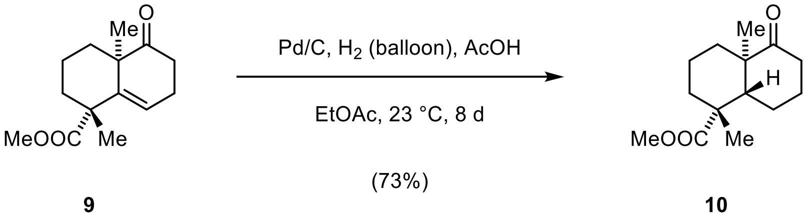

9 **10**

Decalin 10. A suspension of alkene **9** (6.00 g, 25.4 mmol, 1 equiv) and palladium on charcoal (10 wt.%, 2.03 g, 1.90 mmol, 7.50 mol%) in acetic acid (2.00 mL, 34.9 mmol, 1.38 equiv) and ethyl acetate (50 mL) was sparged with hydrogen gas 15 min. The mixture was stirred at 23 °C under an hydrogen atmosphere. After 8 d, the mixture was was filtered over a plug of silica and the filtrate was concentrated. The residue was purified by flash column chromatography (15% diethyl ether in *n*-pentane grading to 30% diethyl ether in *n*-pentane) to afford decalin **10** (4.42 g, 18.5 mmol, 73%) as a white crystalline solid.

TLC (10% ethyl acetate in *n*-pentane): R_f = 0.29 (CAM)

Melting point: 72–73 °C

$[\alpha]_D^{20}$ = +0.96 (c = 0.333 in dichloromethane)

^{1}H-NMR (400 MHz, $CDCl_3$): δ = 3.69 (s, 3H), 2.58 (td, *J* = 14.1, 6.4 Hz, 1H), 2.29 – 2.12 (m, 3H), 2.12 – 2.01 (m, 2H), 1.82 (tt, *J* = 13.6, 3.6 Hz, 1H), 1.73 – 1.68 (m, 1H), 1.60 – 1.52 (m, 2H), 1.49 (t, *J* = 4.2 Hz, 1H), 1.40 (dd, *J* = 12.6, 2.9 Hz, 1H), 1.21 (s, 3H), 1.05 – 1.01 (m, 1H), 0.98 (d, *J* = 0.8 Hz, 3H) ppm.

^{13}C-NMR (101 MHz, $CDCl_3$): δ = 215.6, 177.6, 54.8, 51.6, 49.6, 44.5, 38.2, 37.9, 33.4, 28.8, 26.6, 23.0, 19.0, 17.2 ppm.

IR (ATR, neat): $\tilde{\nu}$ = 2948 (m), 2875 (w), 1725 (s), 1705 (s), 1449 (m), 1379 (w), 1315 (w), 1249 (m), 1225 (m), 1209 (m), 1150 (s), 1100 (m), 1048 (w), 980 (w), 949 (w), 820 (w), 773 (w).

HRMS (ESI): calcd for $C_{14}H_{23}O_3$ $[M+H]^+$: 239.1642; found: 239.1651.

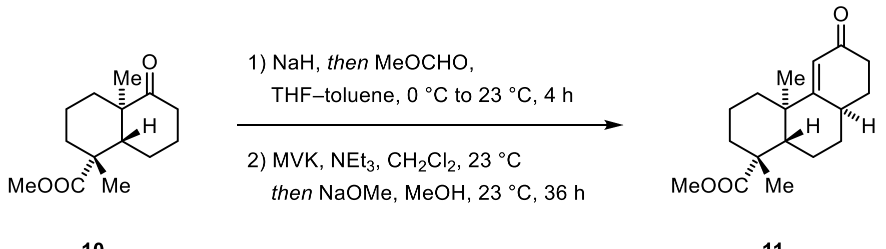

10 (41% over two steps) **11**

Enone 11. To a solution of decalin **10** (4.40 g, 18.5 mmol, 1 equiv) in a mixture of tetrahydrofuran (220 mL) and toluene (110 mL) was added sodium hydride suspended in mineral oil (60.0 wt.%, 2.95 g, 73.8 mmol, 4.00 equiv) in one portion at 0 °C. Upon addition the color of the mixture changed from colorless to yellow. After 60 min, methyl formate (16.1 mL, 258 mmol, 14.0 equiv) was added dropwise to the suspension. The reaction mixture was warmed to 23 °C. After 4 h, saturated aqueous ammonium chloride solution (50 mL) was added and the layers were separated. The aqueous layer was extracted with ethyl acetate

(3 × 50 mL). The combined organic layers were washed with saturated aqueous sodium chloride solution (50 mL) and dried over magnesium sulfate. The dried solution was filtered and the filtrate was concentrated to yield the aldehyde as a yellow oil.

To a solution of crude aldehyde in dichloromethane (123 mL) were added dropwise triethyl amine (10.0 mL, 73.9 mmol, 4.00 equiv) followed by freshly distilled methyl vinyl ketone (7.60 mL, 92.4 mmol, 5.00 equiv) at 23 °C. The mixture was stirred for 48 h under exclusion of light. The mixture was concentrated to yield the crude Michael adduct as a brownish oil. The residue was dissolved in methanol (66 mL) and sodium methoxide (25.0 wt.% in MeOH, 12.0 mL, 55.4 mmol, 3.00 equiv) was added dropwise while stirring at 23 °C. After 24 h, the mixture was diluted with ethyl acetate (200 mL) and saturated aqueous ammonium chloride solution (100 mL) was added. The layers were separated and the aqueous layer was extracted with ethyl acetate (3 × 100 mL). The combined organic layers were washed with saturated aqueous sodium chloride solution (50 mL) and dried over magnesium sulfate. The dried solution was filtered and the filtrate was concentrated. The residue was purified by flash column chromatography on silica gel (20% ethyl acetate in *n*-pentane) to yield enone **11** (2.21 g, 7.59 mmol, 41% over two steps) as a single diastereomer and as a pale yellow foam.

TLC (10% ethyl acetate in *n*-pentane): R_f = 0.29 (UV, CAM)

Melting point: 76–77 °C

$[\alpha]_D^{20}$ = –3.4 (c = 0.793 in dichloromethane)

^{1}H-NMR (400 MHz, $CDCl_3$): δ = 5.89 (d, *J* = 2.0 Hz, 1H), 3.67 (s, 3H), 2.54 (ddtd, *J* = 12.2, 8.8, 5.0, 2.0 Hz, 1H), 2.37 (dt, *J* = 16.1, 4.9 Hz, 1H), 2.30 – 2.23 (m, 1H), 2.22 – 2.18 (m, 1H), 2.10 – 2.06 (m, 1H), 2.05 – 1.95 (m, 3H), 1.94 – 1.85 (m, 1H), 1.76 (dtd, *J* = 12.8, 3.4, 1.6 Hz, 1H), 1.68 – 1.50 (m, 3H), 1.48 – 1.38 (m, 1H), 1.30 (dd, *J* = 11.7, 3.4 Hz, 1H), 1.20 (s, 3H), 1.06 – 0.99 (m, 1H), 0.95 (d, *J* = 0.9 Hz, 3H) ppm.

^{13}C-NMR (101 MHz, $CDCl_3$): δ = 201.3, 177.6, 175.4, 121.2, 54.5, 51.6, 44.7, 41.3, 37.9, 36.9, 36.1, 35.6, 34.8, 29.5, 28.9, 23.2, 20.1, 19.6 ppm.

IR (ATR, neat): $\tilde{\nu}$ = 2946 (m), 2855 (w), 1723 (s), 1672 (s), 1602 (w), 1450 (m), 1378 (w), 1327 (w), 1262 (w), 1234 (m), 1216 (m), 1147 (m), 1095 (w), 992 (w), 879 (w).

HRMS (LTP): calcd for $C_{18}H_{27}O_3$ $[M+H]^+$: 291.1955; found: 291.1970.

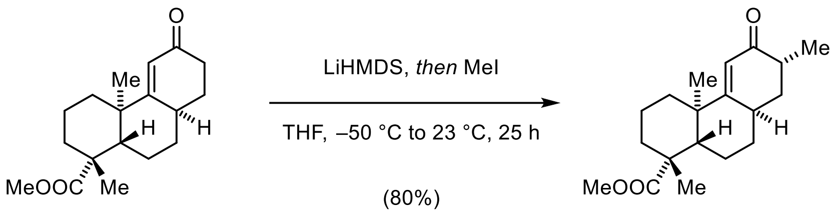

11 **S-13**

α-Methyl enone S-13. To a pale yellow mixture of lithium bis(trimethylsilyl)amide solution (1.00 M in tetrahydrofuran, 7.93 mL, 7.93 mmol, 1.05 equiv) and tetrahydrofuran (100 mL) was added a solution of enone **11** (2.21 g, 7.59 mmol, 1 equiv) in tetrahydrofuran (50mL) dropwise at –50 °C. The obtained bright yellow solution was stirred for 80 min at –50 °C before iodomethane (2.36 mL, 38.0 mmol, 5.00 equiv) was added dropwise. The reaction mixture was warmed slowly to 23 °C. After 24 h, saturated aqueous sodium bicarbonate solution (250 mL) was added and the color of the reaction mixture changed from intense orange to pale yellow. The layers were separated and the aqueous layer was extracted with ethyl acetate (3 × 200 mL). The combined organic layers were washed with saturated aqueous sodium chloride solution and dried over magnesium sulfate. The dried solution was filtered and the filtrate was concentrated. The residue was purified by flash column chromatography on silica gel (10% ethyl acetate in *n*-pentane grading to 20% ethyl acetate in *n*-pentane) to yield α-methyl enone **S-13** (1.85 g, 6.08 mmol, 80%) as a white crystalline solid.

TLC (10% ethyl acetate in *n*-pentane): R_f = 0.54 (UV, CAM)

Melting point: 152–153 °C

$[\alpha]_D^{20}$ = +43.6 (c = 0.280 in dichloromethane)

^{1}H-NMR (400 MHz, $CDCl_3$): δ = 5.79 (d, *J* = 1.5 Hz, 1H), 3.68 (s, 3H), 2.62 (dq, *J* = 11.1, 5.3 Hz, 1H), 2.50 – 2.36 (m, 1H), 2.25 – 2.18 (m, 1H), 2.13 – 2.03 (m, 1H), 2.03 – 1.89 (m, 3H), 1.89 – 1.83 (m, 1H), 1.78 – 1.58 (m, 3H), 1.57 – 1.43 (m, 1H), 1.35 – 1.23 (m, 2H), 1.19 (s, 3H), 1.09 (d, *J* = 7.0 Hz, 3H), 1.02 (dd, *J* = 13.5, 4.2 Hz, 1H), 0.96 (d, *J* = 0.8 Hz, 3H) ppm.

^{13}C-NMR (101 MHz, $CDCl_3$): δ = 204.1, 177.6, 174.1, 119.4, 55.1, 51.6, 44.7, 41.4, 38.1, 38.0, 36.9, 36.4, 34.9, 32.7, 28.9, 23.6, 19.7, 19.0, 15.7 ppm.

IR (ATR, neat): $\tilde{\nu}$ = 2923 (m), 2853 (w), 1721 (s), 1670 (s), 1612 (w), 1449 (m), 1376 (w), 1328 (w), 1223 (m), 1162 (s), 1140 (s), 1093 (w), 1069 (w), 1038 (w), 981 (w), 875 (m), 848 (w), 818 (w).

HRMS (ESI): calcd for $C_{19}H_{29}O_3$ $[M+H]^+$: 305.2111; found: 305.2107.

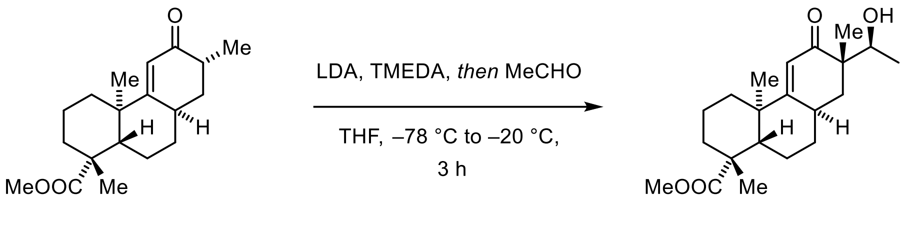

S-14 S-15

Secondary alcohol S-15. To a solution of *N*,*N*-diisopropylamine (3.42 mL, 24.3 mmol, 4.00 equiv) in tetrahydrofuran (40mL) was added a solution of *n*-butyllithium (2.48 M in hexanes, 7.35 mL, 18.2 mmol, 3.00 equiv) dropwise at –78 °C. After 5 min, *N*,*N*,*N'*,*N'*-tetramethylethane-1,2-diamine (11.0 mL, 72.9 mmol, 12.0 equiv) was added dropwise. After 30 min, a solution of enone **S-14** (1.85 g, 6.08 mmol, 1 equiv) in tetrahydrofuran (60 mL) was added dropwise, whereupon the pale yellow solution turned bright yellow. The reaction mixture was stirred at –78 °C for 1 h, then warmed to –18 °C and stirred at this temperature for 1 h. After cooling to –78 °C, acetaldehyde (6.88 mL, 122 mmol, 20.0 equiv) was added dropwise over 5 min, whereupon the bright yellow solution turned pale yellow. After 30 min, saturated aqueous ammonium chloride solution (50 mL) and water (15 mL) were added. The layers were separated and the aqueous layer was extracted with diethylether (4 × 100 mL). The combined organic layers were dried over magnesium sulfate. The dried solution was filtered and the filtrate was concentrated. The residue was purified by flash column chromatography on silica gel (30% ethyl acetate in *n*-pentane) to yield secondary alcohol **S-15** (2.33 g, 6.02 mmol, 99%) as a white solid.

TLC (50% ethyl acetate in *n*-pentane): R_f = 0.44 (UV, CAM)

Melting point: 134 –135 °C

$[\alpha]_D^{20}$ = –13.2 (c = 0.640 in dichloromethane)

^{1}H-NMR (400 MHz, $CDCl_3$): δ = 5.84 (d, J = 1.9 Hz, 1H), 3.84 (q, J = 6.4 Hz, 1H), 3.67 (s, 3H), 2.62 – 2.51 (m, 1H), 2.24 – 2.19 (m, 1H), 2.05 – 1.88 (m, 5H), 1.78 (dd, J = 10.8, 2.6 Hz, 1H), 1.67 – 1.57 (m, 1H), 1.47 (ddd, J = 17.3, 13.8, 9.2 Hz, 2H), 1.30 (dd, J = 12.0, 3.4 Hz, 1H), 1.20 (s, 3H), 1.15 (d, J = 6.4 Hz, 3H), 1.10 – 1.06 (m, 1H), 1.04 (s, 3H), 1.03 – 0.97 (m, 1H), 0.95 (d, J = 0.9 Hz, 3H) ppm.

^{13}C-NMR (101 MHz, $CDCl_3$): δ = 206.2, 177.5, 174.7, 119.6, 69.4, 54.5, 51.6, 48.0, 44.7, 41.1, 40.0, 37.9, 37.0, 36.5, 32.2, 28.8, 23.2, 20.00, 19.6, 17.2, 15.9 ppm.

IR (ATR, neat): $\tilde{\nu}$ = 3447 (br, w), 2935 (br, m), 2853 (w), 1723 (s), 1655 (s), 1450 (m), 1377 (w), 1226 (m), 1187 (w), 1147 (s), 1116 (m), 1082 (m), 1019 (w), 982 (w), 904 (w), 884 (w), 823 (w).

HRMS (ESI): calcd for $C_{21}H_{32}NaO_4$ $[M+Na]^+$: 371.2193; found: 371.2191.

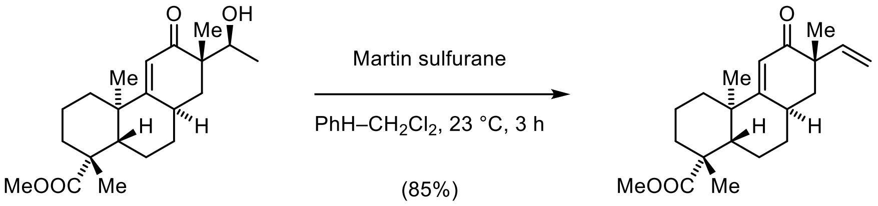

S-15 **12**

Preparation of Martin sulfurane. To a solution of 1,1,1,3,3,3-hexafluoro-2-phenyl-2-propanol (15.0 g, 61.4 mmol, 2.00 equiv) in anhydrous dichloromethane (61 mL) was added sodium hydride suspended in mineral oil (60.0 wt.%, 2.46 g, 61.4 mmol, 2.00 equiv) in portions at 23 °C.[2] After 30 min, diphenyl sulfide (5.16 mL, 30.7 mmol, 1 equiv) and bromine (1.58 mL, 30.7 mmol, 1.00 equiv) were added successively. After 1 h, the resulting suspension was filtered under argon atmosphere to provide a stock solution of Martin's sulfurane in chloroform (0.50 M), which was used without further purification.

α-Vinyl enone 12. To a solution of secondary alcohol **S-15** (2.33 g, 6.02 mmol, 1 equiv) in benzene (40 mL) was added a solution of Martin's sulfurane (0.500 M in dichloromethane, 15.0 mL, 7.52 mmol, 1.25 equiv) dropwise at 23 °C. After 3 h, saturated aqueous sodium bicarbonate solution (30 mL) was added to the reaction mixture. The layers were separated and the aqueous layer was extracted with ethyl acetate (3 × 50 mL). The combined organic layers were dried over sodium sulfate. The dried solution was filtered and the filtrate was concentrated. The residue was purified by flash column chromatography on silica gel (5% ethyl acetate in *n*-pentane grading to 18% ethyl acetate in *n*-pentane) to yield α-vinyl enone **12** (1.69 g, 5.10 mmol, 85%) as a white solid.

TLC (10% ethyl acetate in *n*-pentane): R_f = 0.66 (UV, CAM)

Melting point: 120–121 °C

$[\alpha]_D^{20}$ = –35.5 (c = 0.500 in dichloromethane)

^{1}H-NMR (400 MHz, $CDCl_3$): δ = 5.87 (d, *J* = 2.1 Hz, 1H), 5.74 (dd, *J* = 17.5, 10.7 Hz, 1H), 4.99 (dd, *J* = 10.7, 0.9 Hz, 1H), 4.91 (dd, *J* = 17.5, 0.9 Hz, 1H), 3.66 (s, 3H), 2.56 (dddd, *J* = 12.4, 7.2, 6.1, 3.6 Hz, 1H), 2.27 – 2.14 (m, 1H), 2.07 – 1.94 (m, 4H), 1.93 – 1.84 (m, 1H), 1.79 (dtd, *J* = 12.8, 3.5, 1.5 Hz, 1H), 1.67 – 1.58 (m, 1H), 1.48 (dd, *J* = 13.7, 10.5 Hz, 1H), 1.45 – 1.38 (m, 1H), 1.31 (dd, *J* = 11.5, 3.8 Hz, 1H), 1.20 (s, 3H), 1.15 (s, 3H), 1.12 – 1.05 (m, 1H), 1.02 (dd, *J* = 13.5, 4.2 Hz, 1H), 0.89 (d, *J* = 0.9 Hz, 3H) ppm.

^{13}C-NMR (101 MHz, $CDCl_3$): δ = 202.9, 177.6, 173.8, 140.7, 120.2, 114.3, 54.3, 51.5, 47.8, 44.6, 42.8, 40.9, 37.9, 37.0, 35.9, 32.8, 28.8, 23.1, 23.1, 20.0, 19.6 ppm.

IR (ATR, neat): $\tilde{\nu}$ = 2927 (m), 2854 (w), 1724 (s), 1671 (s), 1631 (w), 1607 (w), 1451 (m), 1377 (w), 1328 (w), 1224 (m), 1187 (w), 1147 (m), 1090 (w), 1034 (w), 992 (w), 917 (w), 884 (w).

HRMS (ESI): calcd for $C_{21}H_{31}O_3$ [M+H]$^+$: 331.2268; found: 331.2276.

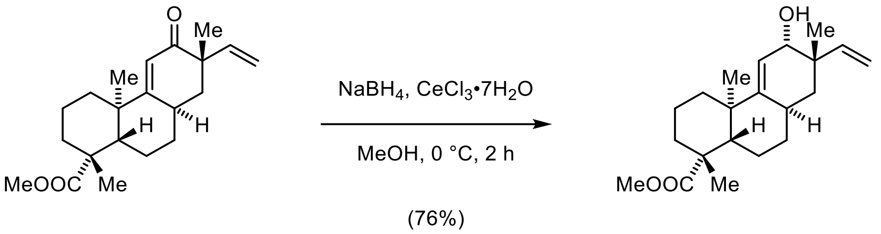

12 **S-16**

Allylic alcohol S-16. A vigorously stirred solution of enone **12** (1.50 g, 4.54 mmol, 1 equiv) in methanol (100 mL) was cooled to 0 °C. Cerium(III) chloride heptahydrate (1.86 g, 4.99 mmol, 1.10 equiv) and sodium tetrahydroborate (343 mg, 9.08 mmol, 2.00 equiv) were sequentially added in one portion. Upon addition, immediate gas evolution was observed. After 2 h, saturated aqueous sodium bicarbonate solution (60 mL), water (15 mL) and ethyl acetate (150 mL) were added. The layers were separated and the aqueous layer was extracted with ethyl acetate (4 × 150 mL). The combined organic layers were washed with saturated aqueous sodium chloride solution (2 × 50 mL) and dried over sodium sulfate. The dried solution was filtered through a plug of silica gel (approx. 5 g) and Celite (approx. 5 g). The filter cake was rinsed with dichloromethane (200 mL) and the solution was concentrated. The residue was purified by flash column chromatography on silica gel (10% ethyl acetate in *n*-pentane) to yield allylic alcohol **S-16** (1.15 g, 3.44 mmol, 76%) as a colorless oil.

TLC (10% ethyl acetate in *n*-pentane): R_f = 0.41 (CAM)

$[\alpha]_D^{20}$ = +13.4 (c = 1.37 in dichloromethane)

^{1}H-NMR (400 MHz, $CDCl_3$): δ = 5.9 (dd, *J* = 17.5, 11.1 Hz, 1H), 5.3 (t, *J* = 1.9 Hz, 1H), 5.2 – 5.1 (m, 1H), 5.2 (dd, *J* = 17.5, 1.7 Hz, 1H), 3.9 (d, *J* = 2.9 Hz, 1H), 3.6 (s, 3H), 2.3 (dddt, *J* = 15.6, 10.5, 5.6, 3.0 Hz, 1H), 2.2 (dtd, *J* = 13.4, 3.3, 1.5 Hz, 1H), 2.0 – 1.8 (m, 4H), 1.8 – 1.7 (m, 1H), 1.7 – 1.7 (m, 1H), 1.6 (dtd, *J* = 14.1, 4.2, 2.2 Hz, 1H), 1.5 – 1.4 (m, 2H), 1.2 – 1.2 (m, 1H), 1.2 (s, 3H), 1.1 (s, 3H), 1.0 (td, *J* = 13.5, 4.1 Hz, 1H), 1.0 – 0.9 (m, 1H), 0.9 (d, *J* = 0.9 Hz, 3H) ppm.

^{13}C-NMR (101 MHz, $CDCl_3$): δ = 177.9, 151.1, 139.9, 120.4, 116.5, 75.3, 55.2, 51.4, 44.6, 43.2, 40.7, 39.5, 38.3, 37.7, 36.5, 31.8, 28.9, 24.0, 23.6, 19.8, 19.3 ppm.

IR (ATR, neat): $\tilde{\nu}$ = 3407 (w,br), 2912 (m), 2948 (m), 1724 (s), 1637 (w), 1449 (m), 1413 (m), 1377 (m), 1326 (w), 1228 (s), 1188 (m), 1147 (s), 1114 (w), 1092 (w), 1041 (m), 989 (m), 909 (m), 891 (w), 852 (w), 822 (w), 773 (w), 731 (m).

HRMS (ESI): calcd for $C_{21}H_{32}NaO_3$ [M+Na]$^+$: 355.2244; found: 355.2238.

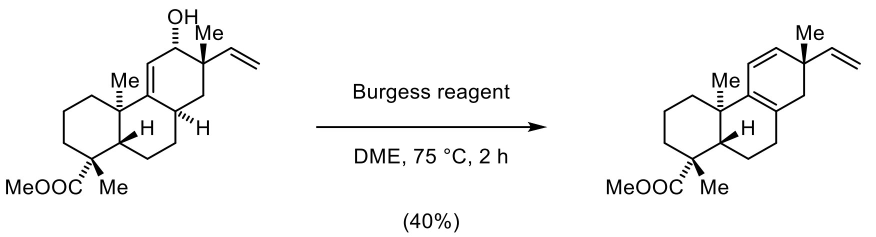

S-16 **13**

Triene 13. A solution of 1-Methoxy-*N*-triethylammonio-sulfonyl-methanimidate (Burgess reagent, 824 mg, 3.46 mmol, 1.10 equiv) in dimethoxyethane (15 mL) was added to allylic alcohol **S-16** (1.05 g, 3.14 mmol, 1 equiv). The mixture was heated to 75 °C. After 2 h, the mixture was diluted with diethyl ether (100 mL) and filtered through a plug of silica. The filter cake was rinsed with diethyl ether (150 mL) and the solution was concentrated. The residue was purified by flash column chromatography on silica gel (2.5% ethyl acetate in *n*-pentane grading to 20% ethyl acetate in *n*-pentane) to yield triene **13** (394 mg, 1.25 mmol, 40%) as a colorless oil.

TLC (4% ethyl acetate in cyclohexane): R_f = 0.36 (UV, CAM)

$[\alpha]_D^{20}$ = –31.1 (c = 0.273 in dichloromethane)

^{1}H-NMR (400 MHz, $CDCl_3$): δ = 5.93 (d, *J* = 9.8 Hz, 1H), 5.82 (dd, *J* = 17.4, 10.6 Hz, 1H), 5.46 (d, *J* = 9.8 Hz, 1H), 4.95 (dd, *J* = 17.5, 1.4 Hz, 1H), 4.90 (dd, *J* = 10.5, 1.4 Hz, 1H), 3.63 (s, 3H), 2.24 – 2.19 (m, 1H), 2.12 – 1.97 (m, 4H), 1.85 (dddd, *J* = 21.6, 15.9, 7.7, 2.9 Hz, 4H), 1.73 (ddd, *J* = 13.2, 11.5, 5.7 Hz, 1H), 1.54 – 1.48 (m, 1H), 1.35 (dd, *J* = 12.5, 1.8 Hz, 1H), 1.21 (s, 3H), 1.07 – 1.04 (m, 1H), 1.02 (s, 3H), 0.80 (s, 3H) ppm.

^{13}C-NMR (101 MHz, $CDCl_3$): δ = 178.1, 146.4, 135.1, 132.4, 127.3, 122.8, 110.8, 53.4, 51.3, 44.0, 42.1, 37.9, 37.5, 37.2, 36.9, 33.5, 28.5, 24.5, 20.8, 19.6, 18.2 ppm.

IR (ATR, neat): $\tilde{\nu}$ = 2956 (m, br), 1728 (s), 1465 (m), 1378 (w), 1232 (m), 1160 (m), 1139 (m), 1099 (w), 1031 (w), 991 (w), 912 (w).

HRMS (ESI): calcd for $C_{21}H_{31}O_2$ [M+H]$^+$:315.2319; found: 315.2318.

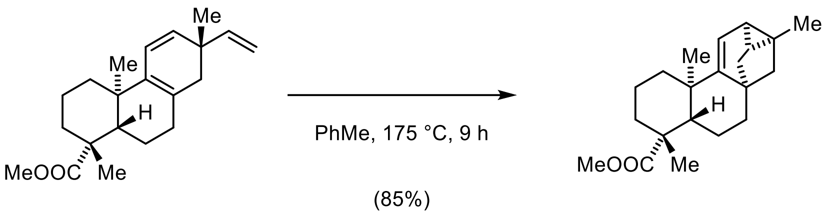

13 **14**

Tricyclooctene 14. A solution of triene **13** (368 mg, 1.12 mmol, 1 equiv) in toluene (40 mL) was heated to 175 °C in a pressure tube. After 9 h, heating was discontinued and the reaction mixture was concentrated. The residue was purified by flash column chromatography on silica gel (1% diethyl ether in *n*-pentane grading to 3% diethyl ether in *n*-pentane) to yield tricyclooctene **14** (313 mg, 994 µmol, 85%) as a colorless oil.

TLC (5% ethyl acetate in cyclohexane): R_f = 0.51 (CAM)

$[\alpha]_D^{20}$ = –26.1 (c = 0.233 in dichloromethane)

^{1}H-NMR (400 MHz, $CDCl_3$): δ = 5.66 (d, *J* = 6.0 Hz, 1H), 3.63 (s, 3H), 2.19 (dtd, *J* = 13.4, 3.3, 1.6 Hz, 1H), 1.99 – 1.79 (m, 5H), 1.53 (dddd, *J* = 14.2, 12.8, 6.7, 3.3 Hz, 2H), 1.38 (dd, *J* = 10.9, 2.0 Hz, 1H), 1.40 (dd, *J* = 7.7, 5.6 Hz, 1H), 1.33 – 1.29 (m, 1H), 1.29 – 1.25 (m, 1H), 1.24 (s, 3H), 1.20 (d, *J* = 10.9 Hz, 1H), 1.18 (s, 3H), 1.09 (dd, *J* = 7.2, 2.2 Hz, 1H), 1.01 (td, *J* = 13.3, 4.2 Hz, 1H), 0.89 (s, 3H), 0.80 (d, *J* = 10.9 Hz, 1H), 0.70 (d, *J* = 11.0 Hz, 1H) ppm.

^{13}C-NMR (101 MHz, $CDCl_3$): δ = 178.3, 152.8, 114.0, 52.6, 51.3, 44.6, 44.4, 42.1, 39.7, 38.8, 38.2, 38.1, 34.6, 28.5, 24.4, 24.2, 23.6, 23.3, 21.2, 20.1, 18.8 ppm.

IR (ATR, neat): $\tilde{\nu}$ = 2923 (s, br), 2852 (m), 1727 (s), 1460 (m), 1376 (w), 1328 (w), 1216 (m), 1143 (m), 1093 (m), 1033 (w), 841 (w), 803 (m).

HRMS (ESI): calcd for $C_{21}H_{31}O_2$ $[M+H]^+$: 315.2319; found: 315.2297.

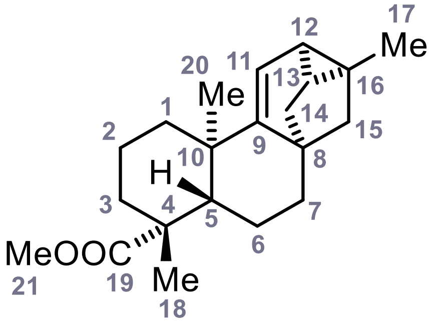

methyl *ent*-trachyloban-9-en-19-oate (14)

Atom number	Isolated **14**[3] (270 MHz, $CDCl_3$) δ_H [ppm]	Synthetic **14** (400 MHz, $CDCl_3$) δ_H [ppm]	$\lvert\Delta\delta_H\rvert$ [ppm]
3	2.21 (ddd, *J* = 13, 3, 3 Hz, 1H)	2.19 (dtd, *J* = 13.4, 3.3, 1.6 Hz, 1H)	0.02
	1.03 (ddd, *J* = 13, 13, 3.5 Hz, 1H)	1.01 (td, *J* = 13.3, 4.2 Hz, 1H)	0.02
11	5.67 (d, *J* = 5.5 Hz, 1H)	5.66 (d, *J* = 6.0 Hz, 1H)	0.01
12	1.40 (dd, *J* = 7, 5.5 Hz, 1H)	1.40 (dd, *J* = 7.7, 5.6 Hz, 1H)	0.00
13	1.11 (dd, *J* = 7, 2 Hz, 1H)	1.09 (dd, *J* = 7.2, 2.2 Hz, 1H)	0.02
14	0.81 (d, *J* = 11 Hz, 1H)	0.80 (d, *J* = 10.9 Hz, 1H)	0.01
	1.40 (dd, *J* = 11, 2 Hz, 1H)	1.38 (dd, *J* = 10.9, 2.0 Hz, 1H)	0.02
15	1.20 (d, *J* = 11 Hz, 1H)	1.20 (d, *J* = 10.9 Hz, 1H)	0.00
	0.72 (d, *J* = 11 Hz, 1H)	0.70 (d, *J* = 11.0 Hz, 1H)	0.02
17	1.25 (s, 3H)	1.24 (s, 3H)	0.01
18	1.19 (s, 3H)	1.18 (s, 3H)	0.01
20	0.90 (s, 3H)	0.89 (s, 3H)	0.01
21	3.64 (s, 3H)	3.63 (s, 3H)	0.01

No ^{13}C-NMR data of isolated **14** available.

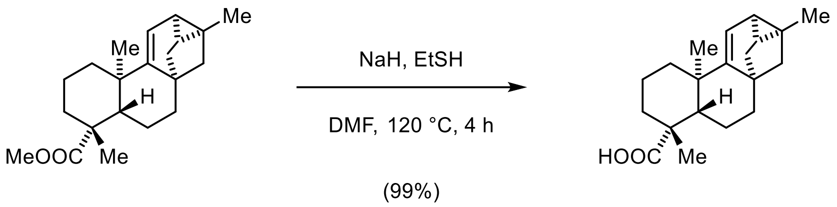

14 **16**

***ent*-Trachyloban-9-en-19-oate 16.** Ethanethiol (50.0 µL, 693 µmol, 10.0 equiv) was added dropwise to a suspension of sodium hydride (60 wt.% in mineral oil, 27.7 mg, 693 µmol, 10.0 equiv) in *N*,*N*-dimethyl formamide (1 mL) at 23 °C and under a slight stream of argon. Upon addition, immediate gas evolution was observed. After 20 min, a solution of methyl ester **14** (21.8 mg, 69.3 µmol, 1 equiv) in *N*,*N*-dimethyl formamide (1 mL) was added and the mixture was heated to 120 °C. The colorless solution turned ocher upon heating. After 5 h, heating was discontinued and aqueous hydrochloric acid was added (2 M, 15 mL). The aqueous layer was extracted with ethyl acetate (4 × 20 mL) and the combined organic layers were dried over magnesium sulfate. The dried solution was filtered and the filtrate was concentrated. Residual *N*,*N*-dimethyl formamide was removed by azeotropic distillation with *n*-heptane. The residue was purified by flash column chromatography on silica gel (10% ethyl acetate and 2% acetic acid in *n*-pentane) to yield acid **16** (20.8 mg, 69.2 µmol, 99%) as a colorless oil.

TLC (10% ethyl acetate and 2% acetic acid in *n*-pentane): R_f = 0.51 (CAM)

$[\alpha]_D^{20}$ = –72.6 (c = 1.39 in dichloromethane)

^{1}H-NMR (400 MHz, $CDCl_3$): δ = 5.66 (d, *J* = 6.0 Hz, 1H), 2.17 (ddd, *J* = 13.3, 4.1, 2.2 Hz, 1H), 2.07 – 1.80 (m, 6H), 1.53 (tdd, *J* = 9.8, 7.6, 3.7 Hz, 2H), 1.42 – 1.27 (m, 5H), 1.24 (s, 3H), 1.23 (s, 3H), 1.19 (d, *J* = 11.0 Hz, 1H), 1.10 (dd, *J* = 6.9, 2.0 Hz, 1H), 0.99 (s, 3H), 0.80 (d, *J* = 11.0 Hz, 1H), 0.70 (d, *J* = 11.0 Hz, 1H) ppm.

^{13}C-NMR (101 MHz, $CDCl_3$): δ = 183.5, 152.7, 114.0, 52.5, 44.6, 44.3, 42.1, 39.7, 38.9, 38.1, 38.0, 34.4, 28.6, 24.5, 24.2, 23.6, 23.3, 21.1, 20.0, 18.8 ppm.

IR (ATR, neat): $\tilde{\nu}$ = 2922 (s), 2852 (s), 1693 (s), 1464 (m), 1411 (w), 1376 (w), 1327 (w), 1261 (m), 1177 (w), 959 (w), 841 (w), 801 (w).

HRMS (ESI): calcd for $C_{20}H_{29}O_2$ $[M+H]^+$: 301.2162; found: 301.2164.

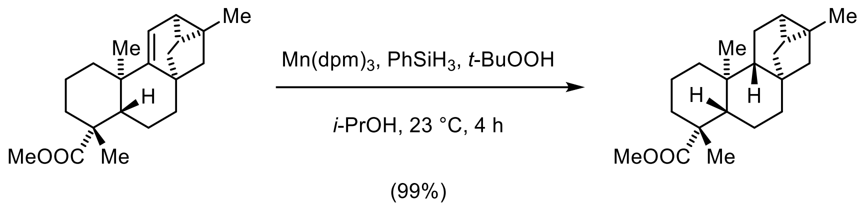

14 **15**

Methyl *ent*-trachyloban-19-oate 15. To a stirred pale brown solution of manganese(III) (Z)-2,2,6,6-tetramethyl-5-oxohept-3-en-3-olate (144 mg, 238 µmol, 0.250 equiv) and alkene **14** (300 mg, 954 µmol, 1 equiv) in degassed (sparged with argon for 10 min while sonicated) isopropanol (13 mL) was added phenylsilane (270 µL, 2.19 mmol, 2.30 equiv) at 23 °C. Upon addition, a colorless solution was obtained. A solution of *tert*-butyl hydroperoxide in decane (5.5 M, 538 µL, 2.96 mmol, 3.10 equiv) was added dropwise. After 3 h, an additional portion of phenylsilane (50.0 µL, 406 µmol, 0.426 equiv) and *tert*-butyl hydroperoxide (5.5 M in decane, 100 µL, 550 µmol, 0.576 equiv) were added dropwise in sequence. After 1 h, the reaction mixture was concentrated. The residue was dissolved in ethyl acetate (15 mL) and saturated aqueous sodium thiosulfate solution was added (15 mL). The layers were separated and the aqueous layer was extracted with ethyl acetate (3 × 20 mL). The combined organic layers were dried over magnesium sulfate and the dried solution filtered. The filtrate was concentrated and the residue was purified by flash column chromatography on silica gel (2% diethyl ether in *n*-pentane) to yield methyl ester **15** (300 mg, 948 µmol, 99%) as a colorless solid.

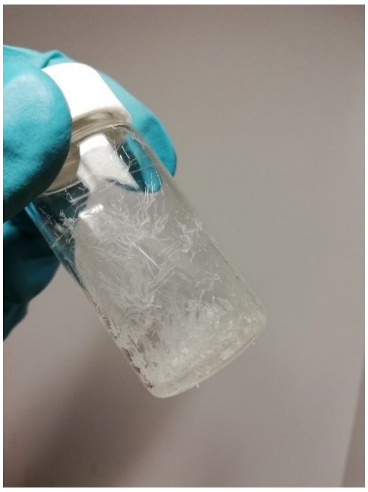

Figure 1| 300 mg of colorless needles of methyl ester **15**.

TLC (3% diethyl ether in *n*-pentane): R_f = 0.43 (CAM)

Melting point: 82–83 °C

$[\alpha]_D^{20}$ = –44.2 (c = 0.907 in dichloromethane)

^{1}**H-NMR** (400 MHz, $CDCl_3$): δ = 3.63 (s, 3H), 2.13 (dtd, *J* = 13.4, 3.4, 1.7 Hz, 1H), 2.02 (d, *J* = 11.7 Hz, 1H), 1.94 – 1.80 (m, 2H), 1.80 – 1.71 (m, 2H), 1.69 – 1.59 (m, 2H), 1.56 – 1.51 (m, 1H), 1.44 (dt, *J* = 12.6, 3.1 Hz, 1H), 1.39 – 1.36 (m, 1H), 1.34 – 1.30 (m, 1H), 1.22 (d, *J* = 11.3 Hz, 1H), 1.19 – 1.16 (m, 1H), 1.14 (s, 3H), 1.12 (s, 3H), 1.07 (ddd, *J* = 11.3, 7.4, 2.0 Hz, 1H), 0.99 – 0.93 (m, 2H), 0.83 – 0.77 (m, 2H), 0.76 (s, 3H), 0.59 – 0.53 (m, 1H) ppm.

^{13}**C-NMR** (101 MHz, $CDCl_3$): δ = 178.0, 57.0, 52.7, 51.1, 50.3, 43.7, 40.7, 39.5, 39.2, 38.6, 38.1, 33.1, 28.7, 24.2, 22.4, 21.8, 20.5 (2 × C), 19.7, 18.8, 12.3 ppm.

IR (ATR, neat): $\tilde{\nu}$ = 2922 (br, m), 2853 (m), 2181 (w), 1727 (s), 1465 (m), 1377 (w), 1235 (m), 1196 (m), 1149 (s), 1096 (m), 1045 (m), 990 (w), 846 (m), 738 (w), 699 (w).

HRMS (ESI): calcd for $C_{21}H_{33}O_2$ $[M+H]^+$: 317.2475; found: 317.2474.

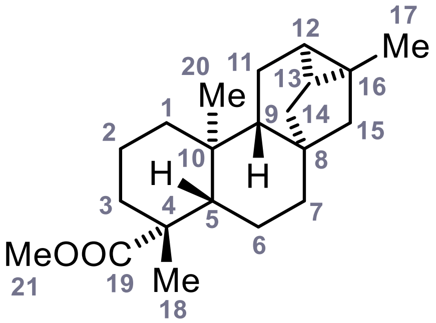

methyl *ent*-trachyloban-19-oate (15)

Atom number	Natural **15**[4] (200 MHz, $CDCl_3$) δ_H [ppm]	Synthetic **15** (400 MHz, $CDCl_3$) δ_H [ppm]	$\lvert\Delta\delta_H\rvert$ [ppm]
12	0.54 (m, 1H)	0.59 – 0.53 (m, 1H)	0.00
13	0.80 (m, 1H)	0.83 – 0.77 (m, 1H)	0.00
17	1.12 (s, 3H)	1.12 (s, 3H)	0.00
18	1.17 (s, 3H)	1.14 (s, 3H)	0.03
20	0.73 (s, 3H)	0.76 (s, 3H)	0.03
21	3.60 (s, 3H)	3.63 (s, 3H)	0.03

Atom number	Natural **15**[5] (68 MHz, $CDCl_3$) δ_C [ppm]	Synthetic **15** (101 MHz, $CDCl_3$) δ_C [ppm]	$\lvert\Delta\delta_C\rvert$ [ppm]
1	39.5	39.5	0.0
2	18.8	18.8	0.0
3	38.1	38.1	0.0
4	43.7	43.7	0.0
5	57.0	57.0	0.0
6	21.8	21.8	0.0
7	39.3	39.2	0.1
8	40.8	40.7	0.1
9	52.7	52.7	0.0
10	38.6	38.6	0.0
11	19.7	19.7	0.0
12	20.5	20.5	0.0
13	24.2	24.2	0.0
14	33.1	33.1	0.0
15	50.4	50.3	0.1
16	22.4	22.4	0.0
17	20.5	20.5	0.0
18	28.7	28.7	0.0
19	177.8	178.0	0.2
20	12.3	12.3	0.0
21	–	51.1	–

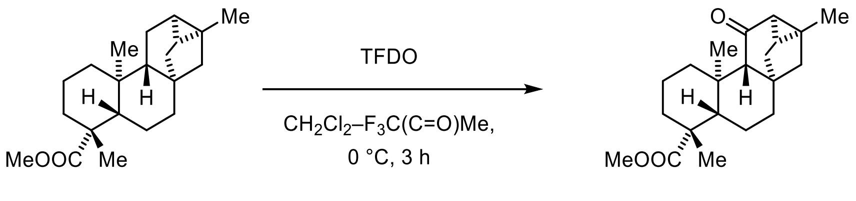

15 (43%) 18

Preparation of a methyl(trifluoromethyl)dioxirane (TFDO) stock solution. According Baran's procedure[6], a vigorously stirred slurry mixture of sodium bicarbonate (10.8 g, 129 mmol, 1.14 equiv) and water (10.8 mL) in a 3-necked flask was cooled to 0 °C. The flask was equipped with a Hickman distill, which was cooled to –78 °C. Oxone (20.0 g, 32.5 mmol, 0.289 equiv) was added in portions, whereupon an immediate gas evolution was observed. After 5 min, 1,1,1-trifluoroacetone (10.0 mL, 113 mmol, 1 equiv) was added and a clear yellow liquid started to condense at the Hickman distill. After 15 min, the freshly prepared stock solution of TFDO (5.0 mL) was transfered to a light protected flask and molecular sieves (0.3 g) were added.

0.5 mL of the above solution was added to a solution of potassium iodide (2.21 g, 13.3 mmol) in a mixture of acetic acid (1 mL) and water (30 mL). The resulting brownish solution was titrated with a solution of sodium thiosulfate in water (0.02 M). 17.0 mL were used, which corresponds to a concentration of 0.34 M TFDO in 1,1,1-trifluoroacetone.

11-Oxomethyl *ent*-trachylobanoate 18. To a solution of ester **15** (2.7 mg, 8.5 µmol, 1 equiv) in dichloromethane (85 µL) was added the freshly prepared stock solution of TFDO (0.34 M, 75 µL, 26 µmol, 3.0 equiv) at 0 °C. After 3 h, the solvent was removed and the residue was purified by flash column chromatography on silica gel (10% ethyl acetate in *n*-pentane) to yield C-11-ketone **18** (1.2 mg, 3.6 µmol, 43%) as a colorless wax.

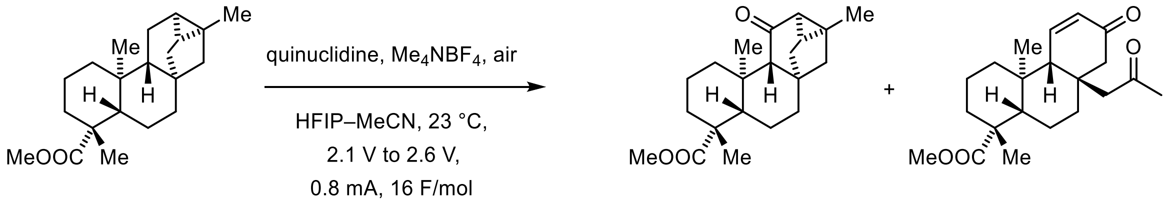

15 18 (36%) 20 (10%)

11-Oxomethyl *ent*-trachylobanoate 18 and enone 20. To a solution of ester **15** (10.6 mg, 33.5 µmol, 1 equiv), quinuclidine (7.5 mg, 67 µmol, 2.0 equiv) and tetramethylammonium tetrafluoroborate (67 mg, 0.41 mmol, 0.10 M, 12 equiv) in acetonitrile (3.6 mL) was added 1,1,1,3,3,3-hexafluoropropan-2-ol (0.42 mL) in an open glass vial at 23 °C. The anode

(Reticulated Vitreous Carbon foam) and cathode (Ni) were immerged in the solution, and the reaction mixture was electrolyzed under galvanostatic conditions (0.8 mA, 16 h, 16.0 F/mol, 2.1 V to 2.6 V, IKA® Electrasyn 2.0 utilized as the galvanostat). The reaction mixture was concentrated and the residue was purified by flash column chromatography on silica gel (25% diethyl ether in *n*-pentane) to yield C-11-ketone **18** (4.0 mg, 12 µmol, 36%) as a colorless wax and enone **20** (1.1 mg, 3.2 µmol, 10%) as a pale yellow wax.

Enone 20.

TLC (33% ethyl acetate in *n*-pentane): R_f = 0.33 (UV, CAM)

$[\alpha]_D^{20}$ = –31 (c = 0.073 in dichloromethane)

^{1}H-NMR (600 MHz, $CDCl_3$): δ = 6.88 (dd, *J* = 10.3, 6.1 Hz, 1H), 6.18 (d, *J* = 10.2 Hz, 1H), 3.63 (s, 3H), 2.76 (d, *J* = 18.0 Hz, 1H), 2.44 (s, 2H), 2.40 (d, *J* = 6.1 Hz, 1H), 2.21 (d, *J* = 13.3 Hz, 1H), 2.15 (d, *J* = 18.3 Hz, 1H), 2.06 (s, 3H), 1.94 – 1.88 (m, 1H), 1.87 – 1.80 (m, 1H), 1.76 (d, *J* = 12.5 Hz, 2H), 1.71 (dq, *J* = 7.4, 3.9, 3.4 Hz, 2H), 1.48 (dt, *J* = 14.0, 2.7 Hz, 1H), 1.29 (d, *J* = 2.0 Hz, 1H), 1.22 (s, 3H), 1.21 – 1.19 (m, 1H), 1.11 (td, *J* = 13.6, 4.2 Hz, 1H), 0.74 (s, 3H) ppm.

^{13}C-NMR (151 MHz, $CDCl_3$): δ = 208.1, 200.1, 177.6, 150.1, 130.8, 55.1, 53.7, 53.4, 51.5, 45.5, 44.1, 40.9, 40.3, 39.0, 37.8, 37.3, 32.9, 28.8, 20.2, 19.0, 15.0 ppm.

IR (ATR, neat): $\tilde{\nu}$ = 2917 (s), 2850 (m), 1721 (w), 1671 (w), 1472 (w), 1428 (w), 1265 (w), 1111 (m), 822 (w), 738 (s), 703 (m).

HRMS (ESI): calcd for $C_{21}H_{30}NaO_4$ $[M+Na]^+$: 369.2036; found: 369.2032.

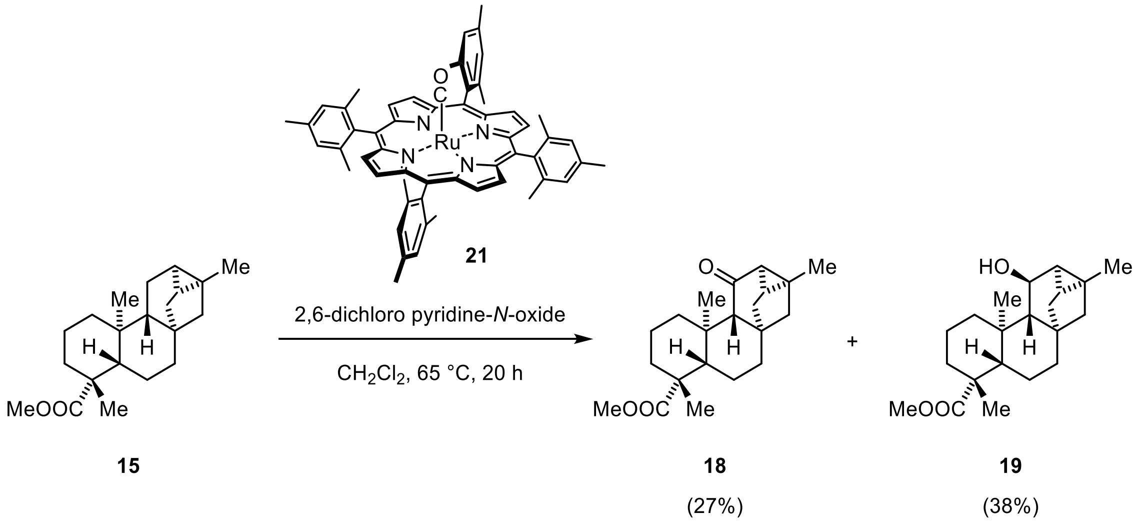

11-Oxomethyl *ent*-trachylobanoate 18 and 11-hydroxymethyl *ent*-trachylobanoate 19. A stock solution of carbonyl[5,10,15,20-tetrakis(2,4,6-trimethylphenyl)-21H,23H-porphinato] ruthenium(II) (**21**) (1.0 mM in dichloromethane, 22 µL, 0.022 µmol, 0.20 mol%) was added to methyl ester **15** (3.5 mg, 11 µmol, 1 equiv) in dichloromethane (80 µL). 2,6-dichloropyridine-*N*-oxide (1.8 mg, 11 µmol, 1.0 equiv) was added and the reaction vial was sealed and heated to 65 °C. After 20 h, heating was discontinued and the mixture was concentrated. The residue was purified by flash column chromatography on silica gel (10% ethyl acetate in *n*-pentane grading to 20% ethyl acetate in *n*-pentane) to yield C-11-ketone **18** (1.0 mg, 3.0 µmol, 27%) as a colorless wax and secondary alcohol **19** (1.4 mg, 4.2 µmol, 38%) as a colorless wax.

11α-Hydroxymethyl *ent*-trachylobanoate 19.

TLC (10% ethyl acetate in *n*-pentane): R_f = 0.22 (CAM)

$[\alpha]_D^{20}$ = –20 (c = 0.093 in dichloromethane)

^{1}H-NMR (400 MHz, $CDCl_3$): δ = 4.11 (d, *J* = 2.7 Hz, 1H), 3.63 (s, 3H), 2.15 (dd, *J* = 13.4, 8.2 Hz, 1H), 1.91 (d, *J* = 11.9 Hz, 1H), 1.83 (ddt, *J* = 27.0, 13.7, 3.4 Hz, 2H), 1.74 (d, *J* = 13.2 Hz, 1H), 1.65 (tdd, *J* = 13.9, 12.1, 3.4 Hz, 1H), 1.49 (tt, *J* = 13.6, 3.1 Hz, 2H), 1.41 (d, *J* = 6.4 Hz, 2H), 1.32 (td, *J* = 13.2, 3.9 Hz, 2H), 1.20 (s, 3H), 1.16 (s, 3H), 1.14 (dd, *J* = 3.1, 1.3 Hz, 1H), 1.09 – 0.97 (m, 4H), 0.93 (dt, *J* = 5.0, 3.0 Hz, 2H), 0.74 (s, 3H) ppm.

^{13}C-NMR (101 MHz, $CDCl_3$): δ = 178.0, 68.3, 64.3, 56.9, 51.3, 49.1, 43.9, 40.3, 40.0, 38.7, 38.3, 38.2, 32.2, 28.8, 28.2, 24.4, 22.0, 21.8, 20.7, 18.8, 14.0 ppm.

IR (ATR, neat): $\tilde{\nu}$ = 3412 (w, br), 2918 (s), 2849 (m), 1722 (m), 1464 (w), 1378 (w), 1265 (m), 1235 (w), 1192 (w), 1153 (w), 987 (w), 738 (s).

HRMS (ESI): calcd for $C_{21}H_{33}O_3$ $[M+H]^+$: 333.2424; found: 333.2422.

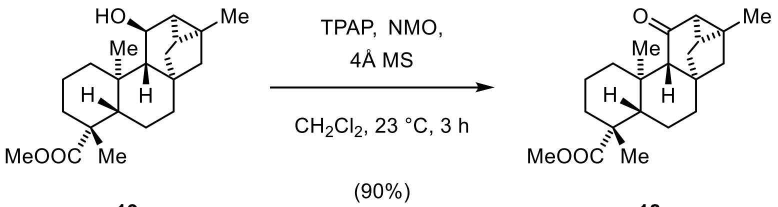

19 **18**

11-Oxomethyl *ent*-trachylobanoate 18. A mixture of secondary alcohol **19** (0.7 mg, 2 µmol, 1 equiv), *N*-methyl morpholine-*N*-oxide (0.7 mg, 6 µmol, 3 equiv), tetrapropylammonium

perruthenate (0.1 mg, 0.4 µmol, 0.2 equiv) and molecular sieves (4 Å, 1 corn) in dichloromethane (1 mL) were stirred at 23 °C. After 3 h, the mixture was filtered through a small plug of silica and concentrated to yield C-11 ketone **18** (0.6 mg, 2 µmol, 90%) as a colorless wax.

11-Oxomethyl *ent*-trachylobanoate 18.

TLC (20% diethyl ether in *n*-pentane): R_f = 0.27 (CAM)

$[\alpha]_D^{20}$ = –9.5 (c = 0.267 in dichloromethane)

^{1}H-NMR (600 MHz, $CDCl_3$): δ = 3.63 (s, 3H), 2.82 (td, *J* = 13.1, 3.8 Hz, 1H), 2.26 (d, *J* = 12.3 Hz, 1H), 2.15 (dt, *J* = 13.4, 3.5 Hz, 1H), 1.86 (dq, *J* = 15.1, 3.8 Hz, 1H), 1.79 (tt, *J* = 13.9, 3.9 Hz, 1H), 1.72 (d, *J* = 7.4 Hz, 2H), 1.66 (d, *J* = 12.2 Hz, 1H), 1.60 (d, *J* = 7.5 Hz, 1H), 1.49 (dd, *J* = 12.3, 2.9 Hz, 1H), 1.40 (s, 1H), 1.36 (d, *J* = 11.9 Hz, 1H), 1.25 (s, 3H), 1.16 (s, 3H), 1.11 (s, 3H), 1.09 – 1.04 (m, 2H), 1.02 (td, *J* = 13.4, 4.4 Hz, 1H), 0.77 (s, 3H) ppm.

^{13}C-NMR (151 MHz, $CDCl_3$): δ = 211.8, 177.7, 65.5, 56.8, 51.2, 48.5, 43.9, 41.4, 40.3, 39.8, 39.7, 38.3, 37.8, 34.2, 31.3, 31.1, 29.0, 21.3, 19.4, 18.8, 14.9 ppm.

IR (ATR, neat): $\tilde{\nu}$ = 2924 (s), 2852 (m), 1726 (s), 1679 (s), 1464 (m), 1382 (w), 1318 (w), 1232 (m), 1190 (m), 1149 (m), 1025 (w), 939 (w), 858 (w), 825 (w).

HRMS (ESI): calcd for $C_{21}H_{31}O_3$ $[M+H]^+$: 331.2268; found: 331.2265.

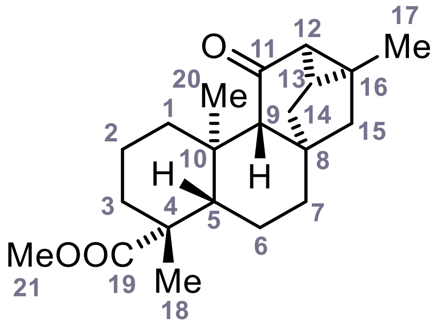

11-oxo methyl *ent*-trachyloban-19-oate (18)

Atom number	Isolated **18**[7] (270 MHz, $CDCl_3$) δ_H [ppm]	Synthetic **18** (400 MHz, $CDCl_3$) δ_H [ppm]	$\lvert\Delta\delta_H\rvert$ [ppm]
1	2.83 (dt, *J* = 13, 3 Hz, 1H)	2.83 (dt, *J* = 13.1, 3.8 Hz, 1H)	0.00
	1.10 (m, 1H)	1.09 – 1.04 (m, 1H)	0.01
3	2.15 (dt, *J* = 13, 3 Hz, 1H)	2.15 (dt, *J* = 13.4, 3.5 Hz, 1H)	0.00
	1.03 (td, *J* = 13, 4 Hz, 1H)	1.02 (td, *J* = 13.4, 4.4 Hz, 1H)	0.01
5	1.10 (m, 1H)	1.09 – 1.04 (m, 1H)	0.01
6	1.87 (dq, *J* = 13, 3 Hz, 1H)	1.86 (dq, *J* = 15.1, 3.8 Hz, 1H)	0.01
9	1.41 (s)	1.40 (s, 1H)	0.01
14	2.27 (d, *J* = 12 Hz, 1H)	2.26 (d, *J* = 12.3 Hz, 1H)	0.01
	1.49 (d)	1.49 (dd, *J* = 12.3, 2.9 Hz, 1H)	0.00
15	1.67 (d, *J* = 12 Hz, 1H)	1.66 (d, *J* = 12.2 Hz, 1H)	0.01
	1.37 (d, *J* = 12 Hz, 1H)	1.36 (d, *J* = 11.9 Hz, 1H)	0.01
17	1.26 (s, 3H)	1.25 (s, 3H)	0.01
18	1.17 (s, 3H)	1.16 (s, 3H)	0.01
20	0.78 (s, 3H)	0.77 (s, 3H)	0.01
21	3.63 (s, 3H)	3.63 (s, 3H)	0.00

Atom number	Isolated **18**[7] (68 MHz, $CDCl_3$) δ_C [ppm]	Synthetic **18** (101 MHz, $CDCl_3$) δ_C [ppm]	$\lvert\Delta\delta_C\rvert$ [ppm]
1	41.4	41.4	0
2	18.8	18.8	0
3	37.9	37.8	0.1
4	43.9	43.9	0
5	56.8	56.8	0
6	21.3	21.3	0
7	38.3	38.3	0
8	39.8	39.8	0
9	65.6	65.5	0.1
10	39.7	39.7	0
11	211.6	211.8	0.2
12	40.2	40.3	0.1
13	31.1	31.1	0
14	34.2	34.2	0
15	48.6	48.5	0.1
16	31.2	31.3	0.1
17	19.4	19.4	0
18	29.9	29.0	0.9*
19	177.6	177.7	0.1
20	14.9	14.9	0
21	51.2	51.2	0

*There is no literature ^{13}C spectrum for comparison available (*Phytochemistry* **1983**, *22*, 2543). We can only speculate that the peak picking for the literature compound was not accurate as "grease" does provide a peak at 29.8 ppm (*J. Org. Chem.* **1997**, *62*, 21, 7512-7515). The ^{13}C shift of closely related compounds (*Phytochemistry* **1983**, *22*, 11, 2543, Table 2) also speak for a potential misassignment (28.6 ppm for compound **9b**, B half).

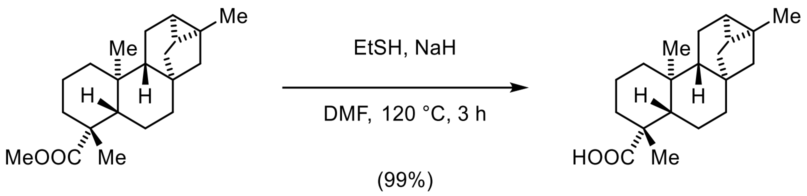

15 **6**

***ent*-Trachylobanoate 6.** Ethanethiol (45.5 µL, 632 µmol, 10.0 equiv) was added dropwise to a suspension of sodium hydride (60.0 wt.% in mineral oil, 25.3 mg, 632 µmol, 10.0 equiv) in *N*,*N*-dimethyl formamide (1 mL) at 23 °C under a slight stream of argon. Upon addition, immediate gas evolution was observed. After 20 min, a solution of methyl ester **15** (20.0 mg, 63.2 µmol, 1 equiv) in *N*,*N*-dimethyl formamide (1 mL) was added and the mixture was heated to 120 °C. The colorless solution turned ocher upon heating. After 3 h, heating was discontinued and aqueous hydrochloric acid was added (2 M, 15 mL), whereupon the solution turned colorless. The aqueous layer was extracted with dichloromethane (4 × 10 mL) and the combined organic layers were washed with saturated aqueous sodium chloride solution (2 × 5 mL). The washed solution was dried over magnesium sulfate and the dried solution was filtered. The filtrate was concentrated and residual *N*,*N*-dimethyl formamide was removed by azeotropic distillation with *n*-heptane. The residue was purified by flash column chromatography on silica gel (10% ethyl acetate and 2% acetic acid in *n*-pentane) to yield acid **6** (19.0 mg, 62.8 µmol, 99%) as a colorless oil.

TLC (10% ethyl acetate and 1% acetic acid in *n*-pentane): R_f = 0.37 (CAM)

$[\alpha]_D^{20}$ = –41.1 (c = 1.24 in dichloromethane)

^{1}H-NMR (400 MHz, $CDCl_3$): δ = 12.68 – 10.05 (m, 1H), 2.17 – 2.07 (m, 1H), 2.04 (d, *J* = 11.8 Hz, 1H), 1.87 (dtd, *J* = 14.0, 11.0, 3.5 Hz, 2H), 1.79 – 1.71 (m, 2H), 1.66 (ddd, *J* = 14.6, 7.6, 2.5 Hz, 1H), 1.56 (d, *J* = 13.1 Hz, 1H), 1.45 (dt, *J* = 13.2, 3.3 Hz, 1H), 1.39 (d, *J* = 11.2 Hz, 1H), 1.33 (dd, *J* = 17.3, 7.4 Hz, 1H), 1.24 (d, *J* = 6.7 Hz, 1H), 1.21 (s, 3H), 1.17 (dd, *J* = 3.3, 1.7 Hz, 1H), 1.13 (s, 3H), 1.10 – 1.04 (m, 1H), 1.00 (dd, *J* = 10.3, 3.5 Hz, 2H), 0.96 – 0.90 (m, 1H), 0.88 (s, 3H), 0.82 (dd, *J* = 8.1, 3.2 Hz, 1H), 0.76 (dd, *J* = 13.4, 4.3 Hz, 1H), 0.57 (dt, *J* = 7.8, 2.7 Hz, 1H) ppm.

^{13}C-NMR (101 MHz, $CDCl_3$): δ = 184.0, 57.0, 52.7, 50.3, 43.6, 40.7, 39.4, 39.2, 38.9, 37.8, 33.1, 28.9, 24.3, 22.4, 21.7, 20.5 (2 × C), 19.7, 18.7, 12.4 ppm.

IR (ATR, neat): $\tilde{\nu}$ = 2921 (m, br), 2855 (m), 1693 (s), 1468 (w), 1263 (m), 1208 (w), 1021 (w), 972 (w), 846 (w), 799 (w), 740 (w), 465 (w).

HRMS (ESI): calcd for $C_{20}H_{30}NaO_2$ $[M+Na]^+$: 325.2138; found: 325.2136.

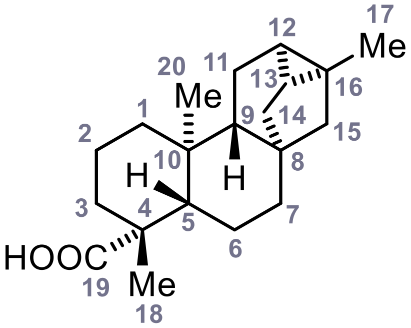

***ent*-trachyloban-19-oate (6)**

Atom number	Natural **6**[8] (500 MHz, $CDCl_3$) δ_H [ppm]	Synthetic **6** (400 MHz, $CDCl_3$) δ_H [ppm]	$\lvert\Delta\delta_H\rvert$ [ppm]
1	0.78 (dd, *J* = 13.1, 4.0 Hz, 1H)	0.76 (dd, *J* = 13.4, 4.3 Hz, 1H)	0.02
2	1.84 (ddd, *J* = 13.7, 10.1, 6.4 Hz, 1H)	1.87 (dtd, *J* = 14.0, 11.0, 3.5 Hz, 2H)	0.03
3	0.99 (ddd, *J* = 13.4, 13.4, 4.3 Hz, 1H)	1.00 (dd, *J* = 10.3, 3.5 Hz, 2H)	0.01
	2.12 (ddd, *J* = 7.3, 3.4, 3.4 Hz, 1H)	2.17 – 2.07 (m, 1H)	–
5	1.00 (dd, *J* = 11.0, 2.8 Hz, 1H)	1.00 (dd, *J* = 10.3, 3.5 Hz, 2H)	0.00
6	1.72 (ddd, *J* = 12.4, 9.2, 1.2 Hz, 1H)	1.79 – 1.71 (m, 2H)	–
	1.75 (dt, *J* = 6.1, 2.7 Hz, 1H)	1.79 – 1.71 (m, 2H)	–
7	1.32 (dt, *J* = 13.1, 4.9 Hz, 1H)	1.33 (dd, *J* = 17.3, 7.4 Hz, 1H)	0.01
	1.45 (dt, *J* = 6.7, 3.4 Hz, 1H)	1.45 (dt, *J* = 13.2, 3.3 Hz, 1H)	0.00
9	1.08 (br., 1H)	1.10 – 1.04 (m, 1H)	–
11	1.67 (ddd, *J* = 14.7, 7.3, 2.4 Hz, 1H)	1.66 (ddd, *J* = 14.6, 7.6, 2.5 Hz, 1H)	0.01
	1.87 (ddd, *J* = 11.4, 3.1, 3.1 Hz, 1H)	1.87 (dtd, *J* = 14.0, 11.0, 3.5 Hz, 2H)	0.00
12	0.58 (dt, *J* = 7.9, 2.4 Hz, 1H)	0.57 (dt, *J* = 7.8, 2.7 Hz, 1H)	0.01
13	0.82 (dd, *J* = 7.9, 3.3 Hz, 1H)	0.82 (dd, *J* = 8.1, 3.2 Hz, 1H)	0.00
14	1.19 (m, 1H)	1.17 (dd, *J* = 3.3, 1.7 Hz, 1H)	–
15	1.23 (d, *J* = 11.3 Hz, 1H)	1.24 (d, *J* = 6.7 Hz, 1H)	0.01
	1.39 (d, *J* = 11.3 Hz, 1H)	1.39 (d, *J* = 11.2 Hz, 1H)	0.00
17	1.14 (s, 3H)	1.13 (s, 3H)	0.01
18	1.21 (s, 3H)	1.21 (s, 3H)	0.00
20	0.88 (s, 3H)	0.88 (s, 3H)	0.00

Atom number	Natural **6** [8] (125 MHz, $CDCl_3$) δ_C [ppm]	Synthetic **6** (101 MHz, $CDCl_3$) δ_C [ppm]	$\lvert\Delta\delta_C\rvert$ [ppm]
1	39.4	39.4	0.0
2	18.7	18.7	0.0
3	37.8	37.8	0.0
4	43.7	43.6	0.1
5	57.0	57.0	0.0
6	21.7	21.7	0.0
7	39.2	39.2	0.0
8	40.7	40.7	0.0
9	52.8	52.7	0.1
10	38.9	38.9	0.0
11	19.7	19.7	0.0
12	20.5	20.5	0.0
13	24.3	24.3	0.0
14	33.1	33.1	0.0
15	50.3	50.3	0.0
16	22.4	22.4	0.0
17	20.5	20.5	0.0
18	28.9	28.9	0.0
19	184.5	184.5	0.0
20	12.4	12.4	0.0

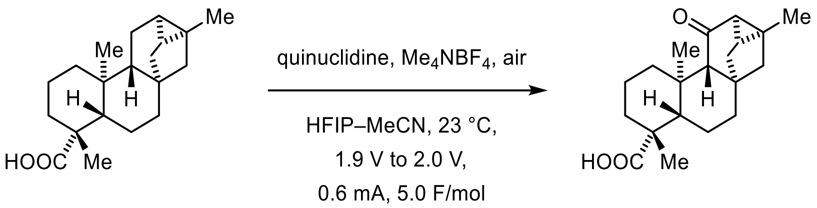

6 **3**

(29%)

11-Oxo *ent*-trachylobanoate 3. To a solution of carboxylic acid **6** (7.2 mg, 24 µmol, 1 equiv), quinuclidine (7.9 mg, 71 µmol, 3.0 equiv) and tetramethylammonium tetrafluoroborate (65 mg, 0.39 mmol, 0.10 M, 16 equiv) in acetonitrile (3.6 mL) was added 1,1,1,3,3,3-hexafluoropropan-2-ol (0.42 mL) in an open glass vial at 23 °C.[Ref] The anode (Reticulated Vitreous Carbon foam) and cathode (Ni) were immerged in the solution, and the reaction mixture was electrolyzed under galvanostatic conditions (0.6 mA, 5 h, 5.0 F/mol, 1.9 V to 2.0 V, IKA® Electrasyn 2.0 utilized as the galvanostat). After full conversion of starting material, aqueous hydrochloric acid (2 M, 10 mL) was added. The aqueous layer was extracted with ethyl acetate (5 × 10 mL) and the combined organic layers were dried over magnesium sulfate. The dried solution was filtered and the filtrate was concentrated. The residue was purified by flash column chromatography on silica gel (10% ethyl acetate and 2% acetic acid in *n*-pentane) to yield C-11-ketone **3** (2.2 mg, 7.0 µmol, 29%) as a colorless wax.

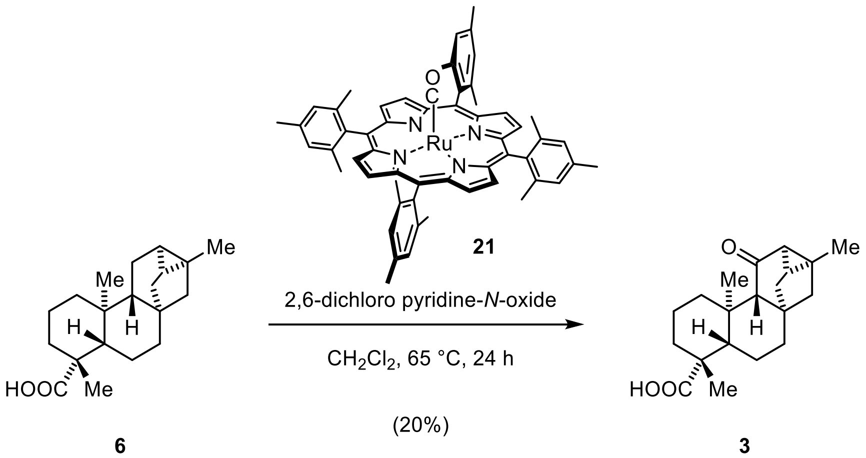

(20%)

6 **3**

11-Oxo *ent*-trachylobanoate 3. A stock solution of carbonyl[5,10,15,20-tetrakis(2,4,6-trimethylphenyl)-21H,23H-porphinato]ruthenium(II) (1.0 mM in dichloromethane, 32 µL, 0.032 µmol, 0.20 mol%) was added to carboxylic acid **6** (4.8 mg, 16 µmol, 1 equiv) in dichloromethane (130 µL). 2,6-dichloropyridine-*N*-oxide (2.6 mg, 16 µmol, 1.0 equiv) was added and the reaction vial was sealed and heated to 65 °C. After 24 h, heating was discontinued and the mixture was concentrated. The residue was purified by flash column

chromatography on silica gel (10% ethyl acetate and 2% acetic acid in *n*-pentane) to yield C-11-ketone **3** (1.0 mg, 3.2 µmol, 20%) as a colorless wax.

TLC (10% ethyl acetate and 2% acetic acid in *n*-pentane): R_f = 0.46 (CAM)

$[\alpha]_D^{20}$ = –56 (c = 0.033 in dichloromethane)

^{1}H-NMR (600 MHz, $CDCl_3$): δ = 2.84 (d, J = 13.4 Hz, 1H), 2.27 (d, J = 12.3 Hz, 1H), 2.14 (d, J = 13.7 Hz, 1H), 1.88 – 1.73 (m, 3H), 1.67 (dd, J = 12.6, 3.7 Hz, 2H), 1.60 (d, J = 7.4 Hz, 1H), 1.49 (dd, J = 12.8, 3.3 Hz, 1H), 1.47 – 1.38 (m, 3H), 1.37 (d, J = 11.9 Hz, 1H), 1.25 (s, 3H), 1.24 (s, 1H), 1.22 (s, 3H), 1.09 (dt, J = 13.5, 3.2 Hz, 2H), 1.04 (td, J = 13.4, 4.3 Hz, 1H), 0.88 (s, 3H) ppm.

^{13}C-NMR (151 MHz, $CDCl_3$): δ = 211.9, 181.2, 65.7, 56.8, 48.7, 43.8, 41.4, 40.4, 40.1, 39.9, 38.4, 37.9, 34.4, 31.5, 31.4, 29.2, 21.4, 19.6, 18.8, 15.2 ppm.

IR (ATR, neat): $\tilde{\nu}$ = 3340 (m, br), 2917 (s), 2849 (s), 1664 (m, br), 1463 (m), 1261 (w), 730 (w).

HRMS (ESI): calcd for $C_{20}H_{29}O_3$ [M+H]$^+$: 317.2111; found: 317.2110.

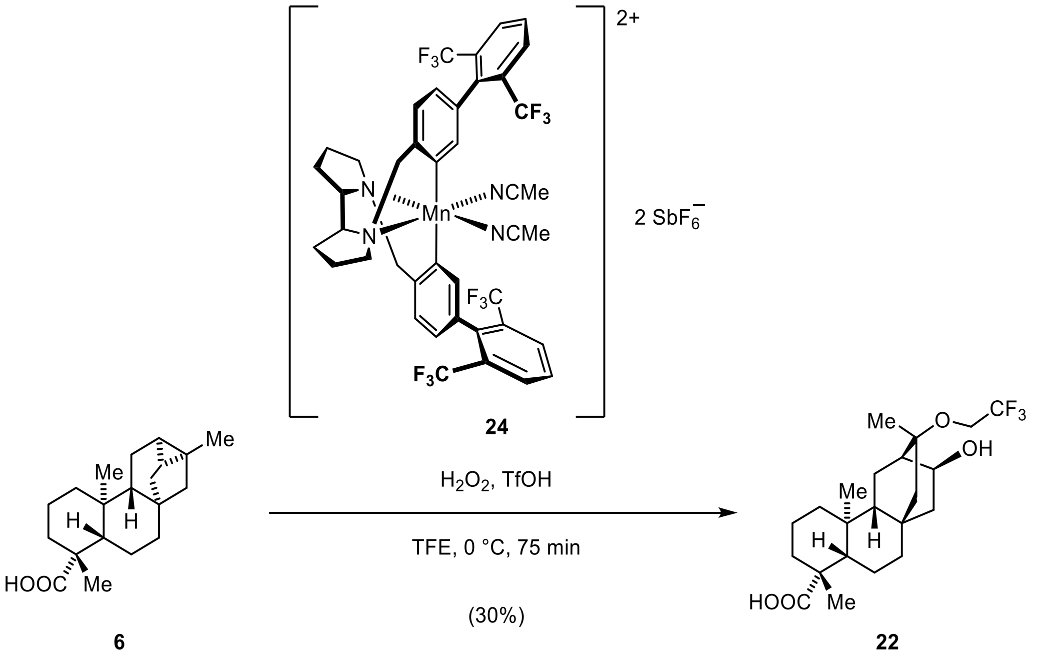

Secondary alcohol 22. (2R,2'R)-1,1'-bis((5-(2,6-bis(trifluoromethyl)phenyl)pyridin-2-yl)methyl)-2,2'-bipyrrolidinebis(acetonitrile)manganese(II) hexafluoroantimonate (0.100 mM in 2,2,2-trifluoroethanol, 3.31 mL, 0.331 µmol, 1.00 mol%) was added to carboxylic acid **6** (10.0 mg, 33.1 µmol, 1 equiv) in 2,2,2-trifluoroethanol (3.5 mL) and the mixture was cooled to

0 °C under air. Triflic acid (30.0 mM in 2,2,2-trifluoroethanol, 3.31 µmol, 0.100 equiv) and hydrogen peroxide (50.0% in water, 6.43 µL, 99.2 µmol, 3.00 equiv) in 2,2,2-trifluoroethanol (100 µL) were added in parallel via syringe pump over 30 min at 0 °C. After 45 min, the mixture was diluted with a mixture of 2% acetic acid in ethyl acetate (40 mL) and filtered through a small plug of silica. The filtrate was concentrated and the residue was purified by flash column chromatography on silica gel (5% ethyl acetate, 2% methanol and 2% acetic acid in cyclohexane) to yield secondary alcohol **21** (4.2 mg, 10 µmol, 30%) as colorless crystals. The structure of **22** was verified by X-ray crystallography.

Melting point: 176–177 °C

TLC (4% methanol in dichloromethane): R_f = 0.28 (CAM)

$[\alpha]_D^{20}$ = –20.3 (c = 0.160 in dichloromethane)

^{1}H-NMR (400 MHz, $CDCl_3$): δ = 3.8 – 3.8 (m, 2H), 3.8 (d, *J* = 3.0 Hz, 1H), 2.5 (ddd, *J* = 13.9, 9.9, 2.9 Hz, 1H), 2.2 (d, *J* = 13.3 Hz, 1H), 2.0 (s, 1H), 1.8 (d, *J* = 15.3 Hz, 2H), 1.6 (d, *J* = 13.8 Hz, 2H), 1.6 (d, *J* = 8.7 Hz, 1H), 1.5 (d, *J* = 10.0 Hz, 2H), 1.4 (ddd, *J* = 10.2, 5.2, 2.6 Hz, 1H), 1.3 (s, 3H), 1.3 (s, 1H), 1.2 (s, 3H), 1.1 (dd, *J* = 13.2, 4.7 Hz, 1H), 1.0 (q, *J* = 4.7, 3.4 Hz, 3H), 1.0 – 0.9 (m, 1H), 0.9 (q, *J* = 4.1 Hz, 3H), 0.8 (s, 3H) ppm.

^{13}C-NMR (101 MHz, $CDCl_3$): δ = 181.5 (reconstructed from HMBC), 125.5 (q, reconstructed from ^{1}H, ^{13}C-HMBC), 82.4, 71.1, 59.9 (q, *J* = 31.3 Hz), 57.1, 52.3, 50.6, 43.6, 42.3, 40.4, 39.8, 38.8, 38.2, 38.1, 29.0, 25.6, 24.1, 22.8, 20.0, 18.8, 12.4 ppm.

IR (ATR, neat): $\tilde{\nu}$ = 2.924 (s), 2852 (m), 1695 (m), 1463 (m), 1379 (w), 1276 (m), 1164 (s), 1108 (m), 1048 (m), 969 (w), 873 (w), 800 (w).

HRMS (ESI): calcd for $C_{22}H_{33}F_3NaO_4$ $[M+Na]^+$: 441.2223; found: 441.2225.

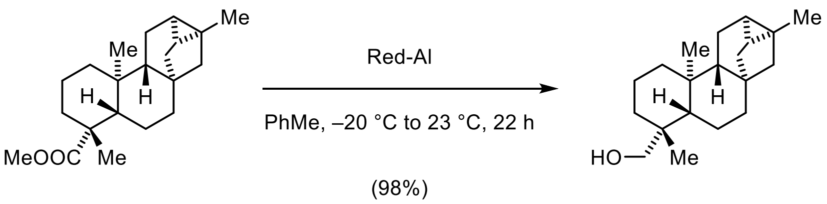

15 **17**

***ent*-Trachyloban-19-ol 17.** A solution of sodium bis(2-methoxyethoxy)aluminium hydride (Red-Al®, 60.0 wt.% in toluene, 291 µL, 894 µmol, 10.0 equiv) was added to a solution ester **15** (28.3 mg, 89.4 µmol, 1 equiv) in toluene (2.5 mL) at –20 °C. Upon addition, slight gas

evolution was observed. After complete addition, the reaction mixture was allowed to slowly warm to 23 °C. After 22 h, the reaction mixture was cooled to 0 °C and excess Red-Al was carefully quenched by the addition of water (5 mL). Diethyl ether (5 mL) was added and the layers were separated. The aqueous layer was extracted with diethyl ether (5 × 10 mL) and the combined organic layers were washed with saturated aqueous sodium chloride solution. The washed solution was dried over magnesium sulfate and the dried solution was filtered. The filtrate was concentrated and the residue was purified by flash column chromatography on silica gel (10% ethyl acetate in *n*-pentane grading to 20% ethyl acetate in *n*-pentane) to yield alcohol **17** (25.2 mg, 87.4 µmol, 98%) as a white solid.

Melting point: 109–110 °C

TLC (10% ethyl acetate in *n*-pentane): R_f = 0.29 (CAM)

$[\alpha]_D^{20}$ = –25.0 (c = 0.900 in dichloromethane)

^{1}H-NMR (400 MHz, $CDCl_3$): δ = 3.72 (d, *J* = 10.9 Hz, 1H), 3.42 (dd, *J* = 11.0, 1.2 Hz, 1H), 2.02 (d, *J* = 11.8 Hz, 1H), 1.87 (ddd, *J* = 14.4, 11.3, 3.1 Hz, 1H), 1.75 (ddd, *J* = 13.6, 5.0, 3.1 Hz, 1H), 1.63 (ddd, *J* = 14.6, 7.0, 2.5 Hz, 1H), 1.60 – 1.52 (m, 2H), 1.51 – 1.48 (m, 1H), 1.44 (dd, *J* = 8.9, 3.6 Hz, 1H), 1.37 (d, *J* = 10.7 Hz, 1H), 1.34 – 1.30 (m, 1H), 1.30 – 1.23 (m, 1H), 1.22 (d, *J* = 10.9 Hz, 1H), 1.17 (d, *J* = 10.2 Hz, 1H), 1.14 (d, *J* = 3.0 Hz, 1H), 1.12 (s, 3H), 1.10 (d, *J* = 6.5 Hz, 1H), 0.93 (s, 3H), 0.91 (s, 3H), 0.90 (s, 1H), 0.88 (dd, *J* = 4.6, 1.5 Hz, 1H), 0.80 (dd, *J* = 7.8, 3.3 Hz, 1H), 0.73 (dd, *J* = 13.8, 4.2 Hz, 1H), 0.56 (dt, *J* = 7.7, 2.8 Hz, 1H) ppm.

^{13}C-NMR (101 MHz, $CDCl_3$): δ = 65.6, 56.8, 53.4, 50.4, 40.7, 39.4, 39.3, 38.3, 38.2, 35.7, 33.4, 26.8, 24.2, 22.4, 20.6, 20.5, 20.3, 19.9, 17.8, 15.1 ppm.

IR (ATR, neat): $\tilde{\nu}$ = 3338 (w,br), 2919 (s), 2857 (s), 1444 (m), 1368 (w), 1291 (w), 1023 (m), 973 (m), 930 (w), 844 (w), 788 (w).

HRMS (ESI): calcd for $C_{20}H_{32}NaO$ $[M+Na]^+$: 311.2345; found: 311.2343.

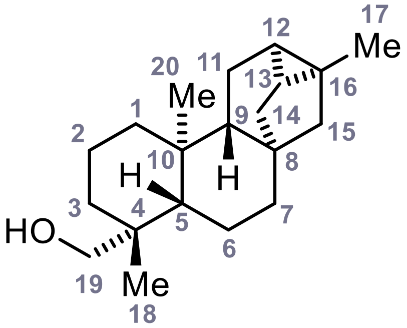

ent-trachyloban-19-ol (17)

Atom number	Natural **17**[8] (500 MHz, $CDCl_3$) δ_H [ppm]	Synthetic **17** (400 MHz, $CDCl_3$) δ_H [ppm]	$\lvert\Delta\delta_H\rvert$ [ppm]
1	0.72 (dd, *J* = 13.4, 3.7 Hz, 1H)	0.73 (dd, *J* = 13.8, 4.2 Hz, 1H)	0.01
	–	1.60 – 1.52 (m, 1H)	–
2	1.31 (m, 1H)	1.34 – 1.29 (m, 1H)	0.00
	1.48 (m, 1H)	1.51 – 1.48 (m, 1H)	0.00
3	0.88 (m, 1H)	0.90 (s, 1H)	0.02
	1.73 (ddt, *J* = 11.9, 1.8, 1.5 Hz, 1H)	1.75 (ddd, *J* = 13.6, 5.0, 3.1 Hz, 1H)	0.02
5	0.86 (dd, *J* = 3.7, 1.5 Hz, 1H)	0.88 (dd, *J* = 4.6, 1.5 Hz, 1H)	0.02
6	1.13 (m), 1H	1.14 (d, *J* = 3.0 Hz, 1H)	0.01
	1.54 (m, 1H)	1.60 – 1.52 (m, 1H)	0.00
7	1.28 (dd, *J* = 9.2, 3.4 Hz, 1H)	1.30 – 1.23 (m, 1H)	0.00
	1.40 (dd, *J* = 6.7, 3.7 Hz, 1H)	1.44 (dd, *J* = 8.9, 3.6 Hz, 1H),	0.04
9	1.08 (m, 1H)	1.10 (d, *J* = 6.5 Hz, 1H)	0.02
11	1.61 (ddd, *J* = 14.6, 7.0, 2.4 Hz, 1H)	1.63 (ddd, *J* = 14.6, 7.0, 2.5 Hz, 1H)	0.02
	1.85 (ddd, *J* = 11.3, 3.1, 1.6 Hz, 1H)	1.87 (ddd, *J* = 14.4, 11.3, 3.1 Hz, 1H)	0.02
12	0.54 (dt, *J* = 7.9, 2.4 Hz, 1H)	0.56 (dt, *J* = 7.7, 2.8 Hz, 1H)	0.02
13	0.78 (dd, *J* = 7.9, 3.1 Hz, 1H)	0.80 (dd, *J* = 7.8, 3.3 Hz, 1H)	0.02
14	1.12 (m, 1H)	1.17 (d, *J* = 10.2 Hz, 1H)	0.05
	2.00 (d, *J* = 11.9 Hz, 1H)	2.02 (d, *J* = 11.8 Hz, 1H)	0.02
15	1.21 (d, *J* = 11.6 Hz, 1H)	1.22 (d, *J* = 10.9 Hz, 1H)	0.01
	1.35 (d, *J* = 11.3 Hz, 1H)	1.37 (d, *J* = 10.7 Hz, 1H)	0.02
17	1.10 (s, 3H)	1.12 (s, 3H)	0.02
18	0.91 (s, 3H)	0.93 (s, 3H)	0.02
19	3.39 (dd, *J* = 1.09, 0.9 Hz, 1H)	3.42 (dd, *J* = 11.0, 1.2 Hz, 1H)	0.03
	3.69 (d, *J* = 10.7 Hz, 1H)	3.72 (d, *J* = 10.9 Hz, 1H)	0.03
20	0.89 (s, 3H)	0.91 (s, 3H)	0.02

Atom number	Natural **17** [8] (125 MHz, $CDCl_3$) δ_C [ppm]	Synthetic **17** (101 MHz, $CDCl_3$) δ_C [ppm]	$\lvert\Delta\delta_C\rvert$ [ppm]
1	39.3	39.3	0.0
2	17.8	17.8	0.0
3	35.7	35.7	0.0
4	38.3	38.3	0.0
5	56.8	56.8	0.0
6	20.3	20.3	0.0
7	39.4	39.4	0.0
8	40.7	40.7	0.0
9	53.4	53.4	0.0
10	38.2	38.2	0.0
11	19.9	19.9	0.0
12	20.6	20.6	0.0
13	24.2	24.2	0.0
14	33.4	33.4	0.0
15	50.3	50.4	0.1
16	22.4	22.4	0.0
17	20.5	20.5	0.0
18	26.8	26.8	0.0
19	65.6	65.6	0.0
20	15.1	15.1	0.0

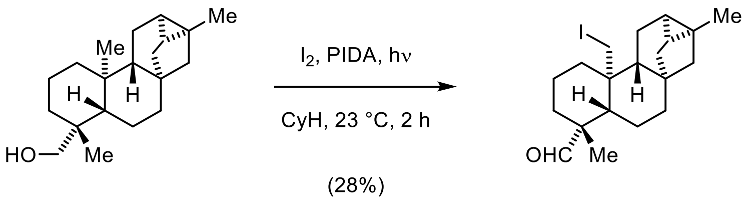

17 **25**

Iodide 25. Alcohol **17** (8.8 mg, 31 µmol, 1 equiv) in cyclohexane (500 µL) was added to a solution of iodine (7.7 mg, 31 µmol, 1.0 equiv) and (diacetoxyiodo)benzene (29 mg, 92 µmol, 3.0 equiv) in cyclohexane (540 µL). The stirred dark purple solution was irradiated with a mercury vapor UVB lamp (160 W) for 2 h at 23 °C. Excess (diacetoxyiodo)benzene was quenched with a mixture of a saturated aqueous sodium thiosulfate/ saturated aqueous sodium bicarbonate solution (1:1, 10 mL). The layers were separated and the aqueous layer was extracted with ethyl acetate (3 × 10 mL). The combined organic layers were dried over magnesium sulfate and the dried solution was filtered. The filtrate was concentrated and the residue was purified by flash column chromatography on silica gel (1% diethyl ether in *n*-pentane grading to 2% diethyl ether in *n*-pentane) to yield iodide **25** (3.5 mg, 8.5 µmol, 28%) as a colorless oil.

TLC (5% diethyl ether in *n*-pentane): R_f = 0.46 (CAM)

$[\alpha]_D^{20}$ = –3.7 (c = 0.233 in dichloromethane)

^{1}H-NMR (400 MHz, $CDCl_3$): δ = 9.69 (d, J = 1.4 Hz, 1H), 3.81 (dd, J = 11.2, 1.8 Hz, 1H), 2.99 (dd, J = 11.2, 1.3 Hz, 1H), 2.74 (ddd, J = 14.7, 8.0, 2.5 Hz, 1H), 2.20 (ddd, J = 13.8, 5.2, 3.4 Hz, 1H), 2.11 (d, J = 12.0 Hz, 2H), 1.90 (dd, J = 11.6, 3.2 Hz, 1H), 1.83 (dd, J = 14.5, 4.2 Hz, 1H), 1.61 (ddt, J = 13.0, 5.4, 3.1 Hz, 2H), 1.51 – 1.48 (m, 1H), 1.46 – 1.41 (m, 4H), 1.38 – 1.34 (m, 1H), 1.34 – 1.28 (m, 3H), 1.13 (s, 3H), 1.04 – 1.02 (m, 1H), 1.01 (s, 3H), 0.95 – 0.93 (m, 1H), 0.57 (d, J = 7.7 Hz, 1H) ppm.

^{13}C-NMR (101 MHz, $CDCl_3$): δ = 205.2, 57.3, 53.3, 51.5, 48.5, 40.5, 39.1, 39.0, 37.8, 33.9, 33.9, 24.6, 24.6, 22.1, 20.4, 20.4, 19.7, 18.8, 18.1, 9.2 ppm.

IR (ATR, neat): $\tilde{\nu}$ = 2919 (s), 1850 (s), 1717 (m), 1462 (m), 1373 (w), 1247 (w), 1018 (w), 846 (w), 726 (w).

HRMS (ESI): calcd for $C_{20}H_{29}NaIO$ [M+Na]$^+$: 435.1155; found: 435.1152.

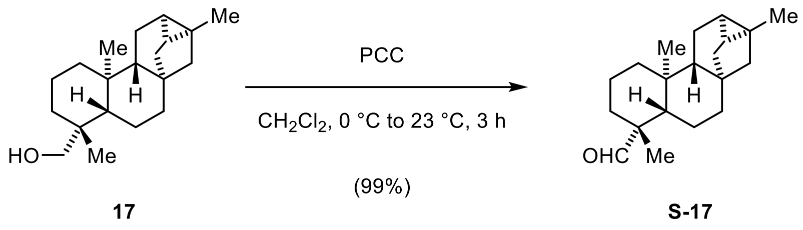

***ent*-Trachyloban-19-al S-17.** To a solution of crude alcohol **17** (18.8 mg, 65.2 µmol, 1 equiv) in dichloromethane (1.10 mL) was added pyridinium chlorochromate (21.5 mg, 97.8 µmol, 1.50 equiv) at 0 °C in one portion. The orange reaction mixture was warmed to 23 °C whereupon it turned brownish. After 3 h, the mixture was diluted with *n*-pentane and filtered through a small plug of silica. The filter cake was washed with a mixture of 20% diethyl ether in *n*-pentane (120 mL) and the filtered solution was concentrated to yield aldehyde **S-17** (18.6 mg, 64.9 µmol, 99%) as a colorless oil.

TLC (5% diethyl ether in *n*-pentane): R_f = 0.68 (CAM)

$[\alpha]_D^{20}$ = –21.6 (c = 0.587 in dichloromethane)

^{1}H-NMR (400 MHz, $CDCl_3$): δ = 9.72 (d, *J* = 1.5 Hz, 1H), 2.10 (dddd, *J* = 13.5, 4.2, 2.7, 1.7 Hz, 1H), 2.03 (d, *J* = 11.7 Hz, 1H), 1.89 (ddd, *J* = 14.5, 11.2, 3.2 Hz, 1H), 1.80 (ddt, *J* = 13.0, 4.8, 2.7 Hz, 1H), 1.66 – 1.62 (m, 1H), 1.60 – 1.48 (m, 4H), 1.43 – 1.34 (m, 3H), 1.25 (d, *J* = 11.2 Hz, 1H), 1.19 (ddd, *J* = 11.7, 3.4, 1.8 Hz, 1H), 1.13 (s, 3H), 1.09 (dd, *J* = 12.6, 2.2 Hz, 1H), 1.04 – 0.99 (m, 1H), 0.97 (s, 3H), 0.97 – 0.92 (m, 1H), 0.83 (dd, *J* = 7.7, 3.2 Hz, 1H), 0.81 (s, 3H), 0.77 – 0.71 (m, 1H), 0.58 (dt, *J* = 7.6, 2.3 Hz, 1H) ppm.

^{13}C-NMR (101 MHz, $CDCl_3$): δ = 206.1, 56.8, 52.3, 50.7, 48.4, 40.7, 39.2, 38.8, 38.7, 34.5, 33.5, 24.4, 24.3, 22.6, 20.6, 20.6, 20.1, 19.9, 18.2, 13.6 ppm.

IR (ATR, neat): $\tilde{\nu}$ = 2926 (s, br), 2852 (s), 1719 (s), 1462 (m), 1386 (w), 1292 (w), 1149 (w), 975 (w), 846 (w), 756 (w), 721 (w).

HRMS (ESI): calcd for $C_{20}H_{30}NaO$ $[M+Na]^+$: 309.2189; found: 309.2190.

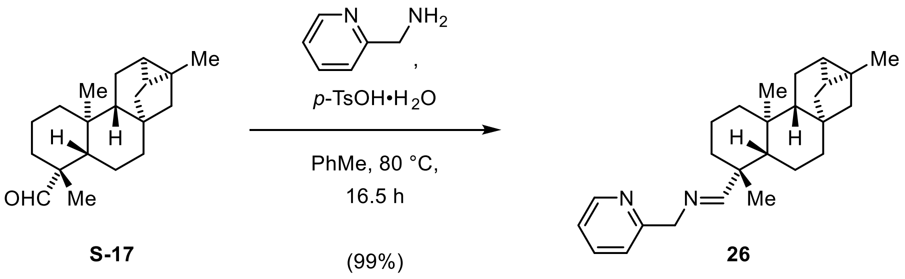

Imine 26. 2-Picolylamine (11.1 mg, 103 µmol, 3.00 equiv) was added to a solution of aldehyde **S-17** (9.80 mg, 34.2 µmol, 1 equiv) in a stock solution of *p*-toluenesulfonic acid hydrate (10.3 mM, 3.42 µmol, 0.100 equiv) in toluene (330 µL) and the mixture was heated to 80 °C. After 16.5 h, heating was discontinued and the mixture was diluted with ethyl acetate (5 mL). Saturated aqueous ammonium chloride solution (2 mL) was added and the layers were separated. The organic layer was sequentially washed with saturated aqueous ammonium chloride solution (2 mL), saturated aqueous sodium bicarbonate solution (2 mL) and saturated aqueous sodium chloride solution (1 mL). The organic layer was dried over sodium sulfate and the dried solution was filtered. The filtrate was concentrated to yield imine **26** (12.8 mg, 34.0 µmol, 99%) as a colorless wax.

TLC (instable on silica)

$[\alpha]_D^{20}$ = –22.1 (c = 0.860 in dichloromethane)

^{1}H-NMR (400 MHz, $CDCl_3$): δ = 8.53 (ddd, *J* = 4.8, 1.8, 0.9 Hz, 1H), 7.84 (q, *J* = 1.4 Hz, 1H), 7.65 (td, *J* = 7.7, 1.8 Hz, 1H), 7.35 (dt, *J* = 7.8, 1.1 Hz, 1H), 7.14 (ddd, *J* = 7.6, 4.9, 1.2 Hz, 1H), 4.73 (d, *J* = 15.0 Hz, 1H), 4.67 (d, *J* = 15.0 Hz, 1H), 2.27 (dt, *J* = 13.1, 4.0 Hz, 1H), 2.01 (d, *J* = 11.7 Hz, 1H), 1.86 (ddd, *J* = 14.4, 11.1, 3.2 Hz, 1H), 1.72 – 1.58 (m, 3H), 1.53 – 1.43 (m, 3H), 1.40 (dd, *J* = 11.3, 2.3 Hz, 2H), 1.36 – 1.31 (m, 1H), 1.23 (s, 1H), 1.18 – 1.14 (m, 1H), 1.12 (s, 3H), 1.11 – 1.09 (m, 1H), 1.09 – 1.05 (m, 1H), 1.03 (t, *J* = 1.4 Hz, 1H), 1.01 (s, 3H), 0.81 (d, *J* = 3.3 Hz, 1H), 0.78 (t, *J* = 4.2 Hz, 1H), 0.73 (s, 3H), 0.56 (dt, *J* = 8.0, 2.8 Hz, 1H) ppm.

^{13}C-NMR (101 MHz, $CDCl_3$): δ = 171.9, 160.0, 149.2, 136.6, 122.0, 121.9, 67.1, 56.9, 52.6, 50.7, 42.0, 40.8, 39.2, 39.2, 38.7, 36.8, 33.5, 28.4, 24.4, 22.6, 20.7, 20.6, 19.9, 19.9, 18.2, 13.8 ppm.

IR (ATR, neat): $\tilde{\nu}$ = 2920 (s, br), 2850 (s), 1720(w), 1661 (m), 1589 (m), 1462 (m), 1435 (m), 1385 (m), 753 (w).

HRMS (ESI): calcd for $C_{26}H_{37}N_2$ $[M+H]^+$: 377.2951; found: 377.2942.

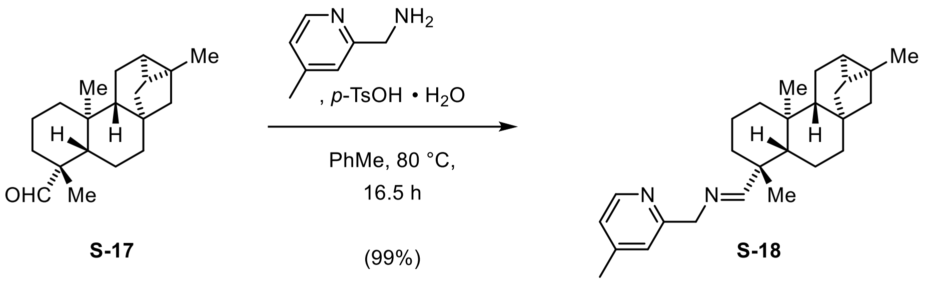

Imine S-18. (4-Methylpyridin-2-yl)methanamine (12.5 mg, 103 µmol, 3.00 equiv) was added to a solution of aldehyde **S-17** (9.80 mg, 34.2 µmol, 1 equiv) in a stock solution of *p*-toluenesulfonic acid hydrate (10.3 mM, 3.42 µmol, 0.100 equiv) in toluene (330 µL) and the mixture was heated to 80 °C. After 16.5 h, heating was discontinued and the mixture was diluted with ethyl acetate (5 mL). Saturated aqueous ammonium chloride solution (2 mL) was added and the layers were separated. The organic layer was washed sequentially with saturated aqueous ammonium chloride solution (2 mL), saturated aqueous sodium bicarbonate solution (2 mL) and saturated aqueous sodium chloride solution (1 mL). The organic layer was dried over sodium sulfate and the dried solution was filtered. The filtrate was concentrated to yield imine **S-18** (13.2 mg, 33.8 µmol, 98%) as a yellow wax.

TLC (instable on silica)

$[\alpha]_D^{20}$ = –28.8 (c = 0.893 in dichloromethane)

^{1}H-NMR (400 MHz, $CDCl_3$): δ = 8.38 (dd, *J* = 5.0, 0.8 Hz, 1H), 7.83 (q, *J* = 1.3 Hz, 1H), 7.18 – 7.13 (m, 1H), 6.96 (dd, *J* = 5.1, 0.9 Hz, 1H), 4.66 (s, 1H), 4.66 – 4.62 (m, 1H), 2.33 (s, 3H), 2.27 (ddd, *J* = 6.6, 3.1, 1.3 Hz, 1H), 2.01 (d, *J* = 11.8 Hz, 1H), 1.87 (ddd, *J* = 14.4, 11.2, 3.2 Hz, 1H), 1.71 – 1.59 (m, 4H), 1.53 – 1.43 (m, 3H), 1.41 – 1.38 (m, 2H), 1.35 – 1.27 (m, 1H), 1.16 (ddd, *J* = 12.0, 3.5, 1.9 Hz, 1H), 1.12 (s, 3H), 1.09 – 1.04 (m, 1H), 1.02 (s, 3H), 1.01 – 0.98 (m, 1H), 0.95 (dd, *J* = 3.8, 2.8 Hz, 1H), 0.79 (q, *J* = 2.0 Hz, 2H), 0.74 (s, 3H), 0.57 – 0.54 (m, 1H) ppm.

^{13}C-NMR (101 MHz, $CDCl_3$): δ = 171.9, 159.6, 149.0, 147.7, 122.9, 122.8, 67.1, 56.9, 52.6, 50.7, 41.9, 40.8, 39.2, 39.2, 38.7, 36.9, 33.5, 28.4, 24.4, 22.6, 21.3, 20.7, 20.6, 19.9, 19.9, 18.2, 13.8 ppm.

IR (ATR, neat): $\tilde{\nu}$ = 2921 (s, br), 2850 (s), 1661 (m), 1605 (m), 1562 (w), 1445 (m), 1384 (w), 1263 (w), 1013 (w), 973 (w), 846 (w), 819 (w), 737 (m).

HRMS (ESI): calcd for $C_{27}H_{39}N_2$ $[M+H]^+$: 391.3108; found: 391.3098.

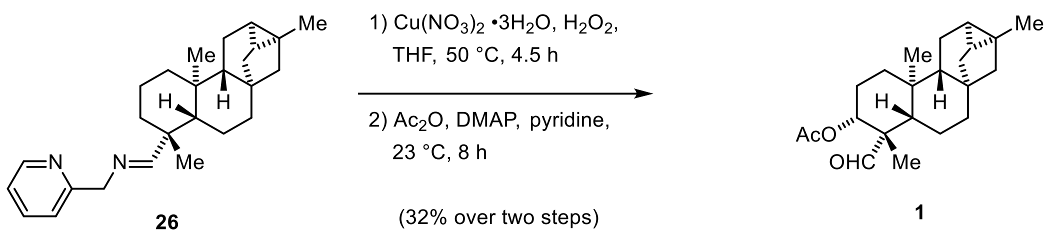

ent-3β-Acetoxy-trachyloban-19-al 1. A mixture of imine **26** (7.1 mg, 19 µmol, 1 equiv) and copper(II) nitrate trihydrate (4.3 mg, 18 µmol, 0.95 equiv) in degassed tetrahydrofuran (0.25 mL) was stirred for 15 min and. The deep blue mixture was heated to 50 °C and aqueous hydrogen peroxide (30%, 19 µL, 0.19 mmol, 10 equiv) was added. Thereby, the turned greenish. After 4.5 h, the mixture was cooled to 23 °C, diluted with ethyl acetate (1 mL) and a saturated aqueous tetrasodium 2,2′,2″,2‴-(ethane-1,2-diyldinitrilo)tetraacetate solution (1 mL) was added. After stirring the biphasic mixture for 18 h, the layers were separated and the aqueous layer was extracted with ethyl acetate (5 × 10 mL). The combined organic layers were dried over sodium sulfate and the dried solution filtered. The filtrate was concentrated and the residue was purified by was filtered through a plug of silica and the filtrate was concentrated. The residue was purified by flash column chromatography on silica gel (12.5% ethyl acetate in *n*-pentane) to yield the intermediate secondary alcohol as mixture with unidentified side products.

A solution of the obtained product mixture and 4-dimethylaminopyridine (7.3 mg, 60 µmol, 3.2 equiv) in pyridine (0.5 mL) was treated with acetic anhydride (0.10 mL, 1.1 mmol, 58 equiv) and stirred for 8 h at 23 °C. The mixture was diluted with ethyl acetate (5mL) and water (5 mL) was added. The layers were separated and the aqueous layer was extracted with ethyl acetate (4 × 10 mL). The combined organic layers were dried over magnesium sulfate and the dried solution was filtered. The filtrate was concentrated and the residue was purified by flash column chromatography on silica gel (2% ethyl acetate in *n*-pentane) to yield acetylated aldehyde **1** (2.1 mg, 6.0 µmol, 32% over two steps) as a colorless wax.

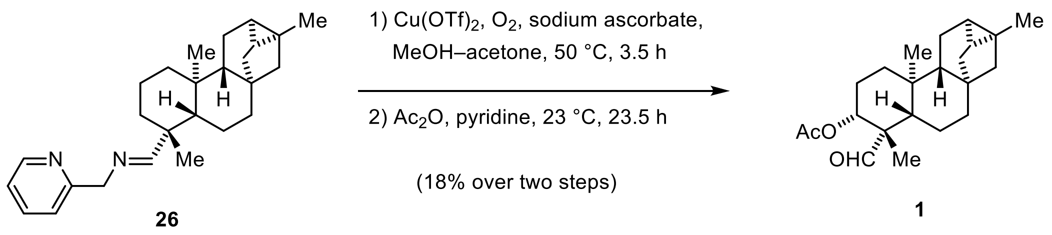

ent-3β-Acetoxy-trachyloban-19-al 1. A mixture of imine **26** (7.5 mg, 20 µmol, 1 equiv) sodium ascorbate (7.9 mg, 40 µmol, 2.0 equiv) and copper(II) triflate (9.4 mg, 26 µmol,

1.3 equiv) in methanol (0.25 mL) and acetone (0.25 mL) was stirred for 5 min and then sparged with oxygen from a balloon for 10 min. Thereby, the brownish mixture turned intense green. The mixture was heated to 50 °C and stirring was continued under an oxygen atmosphere. After 3.5 h, the mixture was cooled to 23 °C, diluted with ethyl acetate (4 mL) and a saturated aqueous tetrasodium 2,2′,2″,2‴-(ethane-1,2-diyldinitrilo)tetraacetate solution (4 mL) was added. After stirring the biphasic mixture for 2 h, the layers were separated and the aqueous layer was extracted with ethyl acetate (3 × 10 mL). The combined organic layers were washed with a saturated aqueous sodium bicarbonate solution and dried over sodium sulfate. The dried solution was filtered through a plug of silica and the filtrate was concentrated.

A solution of the crude product mixture in pyridine (0.5 mL) was treated with acetic anhydride (19 µL, 0.20 mmol, 10 equiv) and stirred for 23.5 h at 23 °C. Water (2 mL) was added and the mixture was extracted with ethyl acetate (4 × 5 mL). The combined organic layers were dried over sodium sulfate and the dried solution was filtered. The filtrate was concentrated and the residue was purified by flash column chromatography on silica gel (1% ethyl acetate in *n*-pentane grading to 3% ethyl acetate in *n*-pentane) to yield acetylated aldehyde **1** (1.2 mg, 3.5 µmol, 18% over two steps) as a colorless wax.

TLC (10% diethyl ether in *n*-pentane)): R_f = 0.37 (CAM)

$[\alpha]_D^{20}$ = –8.5 (c = 0.080 in dichloromethane)

^{1}H-NMR (400 MHz, $CDCl_3$): δ = 10.01 (s, 1H), 4.64 (dd, *J* = 11.9, 5.4 Hz, 1H), 2.04 (s, 3H), 1.96 (d, *J* = 1.7 Hz, 1H), 1.93 (s, 1H), 1.92 – 1.90 (m, 1H), 1.90 – 1.85 (m, 1H), 1.70 (d, *J* = 13.1 Hz, 1H), 1.66 (d, *J* = 3.4 Hz, 1H), 1.65 – 1.59 (m, 1H), 1.51 – 1.43 (m, 1H), 1.40 (d, *J* = 11.4 Hz, 1H), 1.33 (dd, *J* = 13.2, 3.8 Hz, 1H), 1.28 (d, *J* = 3.8 Hz, 1H), 1.23 (d, *J* = 11.7 Hz, 1H), 1.14 (s, 1H), 1.12 (s, 3H), 1.09 (d, *J* = 6.3 Hz, 1H), 1.07 (s, 1H), 1.05 (s, 3H), 1.04 – 0.99 (m, 1H), 0.84 (s, 3H), 0.82 (dd, *J* = 7.8, 3.2 Hz, 1H), 0.58 (dt, *J* = 7.7, 3.0, 2.3 Hz, 1H) ppm.

^{13}C-NMR (101 MHz, $CDCl_3$): δ = 204.7, 170.5, 78.6, 57.0, 51.8, 51.8, 50.2, 40.2, 38.5, 37.8, 36.5, 33.2, 24.1, 23.6, 22.5, 21.1, 20.8, 20.4, 20.3, 20.2, 20.2, 15.3 ppm.

IR (ATR, neat): $\tilde{\nu}$ = 3575 (br, w), 2922 (s), 2852 (s), 1745 (m), 1720 (m), 1462 (m), 1376 (m), 1235 (m), 1028 (m), 798 (w).

HRMS (ESI): calcd for $C_{22}H_{32}NaO_3$ $[M+Na]^+$: 367.2244; found: 367.2244.

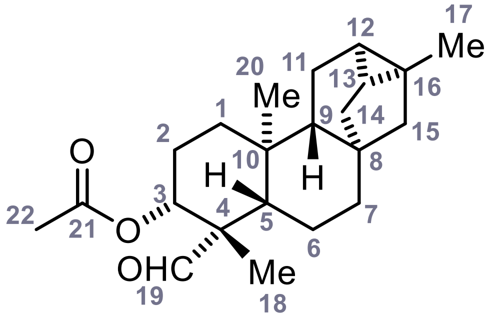

ent-3β-acetoxytrachyloban-19-al (1)

Atom number	Natural **1**[9] (500 MHz, $CDCl_3$) δ_H [ppm]	Synthetic **1** (400 MHz, $CDCl_3$) δ_H [ppm]	$\lvert\Delta\delta_H\rvert$ [ppm]
1	1.01 (m, 1H)	1.04 – 0.99 (m, 1H)	0.00
	1.65 (m, 1H)	1.66 (d, *J* = 3.4 Hz, 1H)	0.01
2	1.88 (brd, 1H)	1.90 – 1.85 (m, 1H)	0.00
	1.96 (m, 1H)	1.96 (d, *J* = 1.7 Hz, 1H)	0.00
3	4.62 (dd, *J* = 12.0, 5.3 Hz, 1H)	4.64 (dd, *J* = 11.9, 5.4 Hz, 1H)	0.02
5	1.06 (m, 1H)	1.09 (d, *J* = 6.3 Hz, 1H)	0.03
6	1.26 (m, 1H)	1.28 (d, *J* = 3.8 Hz, 1H)	0.02
	1.68 (m, 1H)	1.70 (d, *J* = 13.1 Hz, 1H)	0.02
7	1.45 (td, *J* = 13.4, 3.0 Hz, 1H)	1.51 – 1.43 (m, 1H)	0.00
	1.32 (td, *J* = 13.4, 4.0 Hz, 1H)	1.33 (dd, *J* = 13.2, 3.8 Hz, 1H)	0.01
9	1.04 (s, 1H)	1.07 (s, 1H)	0.03
11	1.89 (m, 1H)	1.92 – 1.90 (m, 1H)	0.01
	1.62 (ddd, *J* = 14.5, 7.2, 2.3 Hz, 1H)	1.65 – 1.59 (m, 1H)	0.00
12	0.57 (td, *J* = 7.8, 2.5 Hz, 1H)	0.58 (dt, *J* = 7.7, 3.0, 2.3 Hz, 1H)	0.01
13	0.80 (dd, *J* = 7.8, 3.1 Hz, 1H)	0.82 (dd, *J* = 7.8, 3.2 Hz, 1H)	0.02
14	1.94 (d, *J* = 11.7 Hz, 1H)	1.93 (s, 1H)	0.01
	1.14 (m, 1H)	1.14 (s, 1H)	0.00
15	1.40 (d, *J* = 11.3 Hz, 1H)	1.40 (d, *J* = 11.4 Hz, 1H)	0.00
	1.21 (d, *J* = 11.2 Hz, 1H)	1.23 (d, *J* = 11.6 Hz, 1H)	0.02
17	1.11 (s, 3H)	1.12 (s, 3H)	0.01
18	1.04 (s, 3H)	1.05 (s, 3H)	0.01
19	10.00 (s, 1H)	10.01 (s, 1H)	0.01
20	0.83 (s, 3H)	0.84 (s, 3H)	0.01
22	2.03 (s, 3H)	2.04 (s, 3H)	0.01

Atom number	Natural **1** [9] (125 MHz, $CDCl_3$) Δ_C [ppm]	Synthetic **1** (101 MHz, $CDCl_3$) δ_C [ppm]	$\lvert\Delta\delta_C\rvert$ [ppm]
1	36.5	36.5	0.0
2	23.6	23.6	0.0
3	78.6	78.6	0.0
4	51.8	51.8	0.0
5	57.1	57.0	0.1
6	20.2	20.2	0.0
7	38.5	38.5	0.0
8	40.2	40.2	0.0
9	51.8	51.8	0.0
10	37.9	37.8	0.1
11	20.2	20.2	0.0
12	20.3	20.3	0.0
13	24.1	24.1	0.0
14	33.2	33.2	0.0
15	50.2	50.2	0.0
16	22.5	22.5	0.0
17	20.4	20.4	0.0
18	20.8	20.8	0.0
19	204.6	204.7	0.1
20	15.3	15.3	0.0
21	170.5	170.5	0.0
22	21.1	21.1	0.0

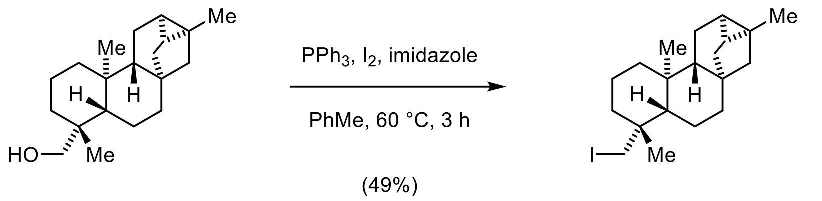

17 **S-19**

***ent*-Trachyloban-19-iodide S-19.** A solution of alcohol **17** (25.8 mg, 89.4 µmol, 1 equiv), imidazole (12.2 mg, 179 µmol, 2.00 equiv) and triphenyl phosphine (30.5 mg, 116 µmol, 1.30 equiv) in toluene (3 mL) was heated to 60 °C. A deep purple solution of iodine (29.5 mg, 116 µmol, 1.30 equiv) in toluene (1 mL) was dropwise added to the mixture, whereupon decolorization and precipitation of a white solid was observed. After complete addition, the mixture turned yellowish. After 3 h, the solvent was removed and the residue was purified by flash column chromatography on silica gel (100% *n*-pentane) to yield iodide **S-19** (17.5 mg, 89.4 µmol, 49%) as a colorless oil.

TLC (100% *n*-pentane): R_f = 0.67 (UV, CAM)

$[\alpha]_D^{20}$ = –11.0 (c = 0.420 in dichloromethane)

^{1}H-NMR (400 MHz, $CDCl_3$): δ = 3.65 (dd, *J* = 9.8, 1.0 Hz, 1H), 3.15 (dd, *J* = 9.8, 2.0 Hz, 1H), 2.01 (d, *J* = 11.8 Hz, 1H), 1.92 – 1.86 (m, 1H), 1.85 – 1.80 (m, 1H), 1.64 (ddd, *J* = 14.6, 6.9, 2.4 Hz, 1H), 1.55 – 1.48 (m, 2H), 1.44 – 1.38 (m, 2H), 1.38 – 1.30 (m, 3H), 1.29 – 1.20 (m, 3H), 1.18 – 1.13 (m, 1H), 1.12 (s, 3H), 1.10 (d, *J* = 2.3 Hz, 1H), 1.04 – 1.00 (m, 1H), 0.99 (d, *J* = 0.9 Hz, 3H), 0.94 (d, *J* = 0.9 Hz, 3H), 0.83 – 0.79 (m, 1H), 0.79 – 0.71 (m, 1H), 0.61 – 0.54 (m, 1H) ppm.

^{13}C-NMR (101 MHz, $CDCl_3$): δ = 55.5, 53.6, 50.4, 40.8, 39.2, 39.2, 39.2, 38.5, 36.5, 33.5, 32.6, 24.4, 22.6, 20.7, 20.7, 20.6, 20.3, 20.0, 17.6, 15.6 ppm.

IR (ATR, neat): $\tilde{\nu}$ = 2920 (s), 2857 (s), 1450 (m), 1378 (w), 1292 (w), 1200 (w), 1015 (w), 973 (w), 844 (w), 788 (w), 753 (w).

HRMS (ESI): calcd for $C_{20}H_{31}$ $[M–I^-]^+$: 271.2420; found: 271.2418.

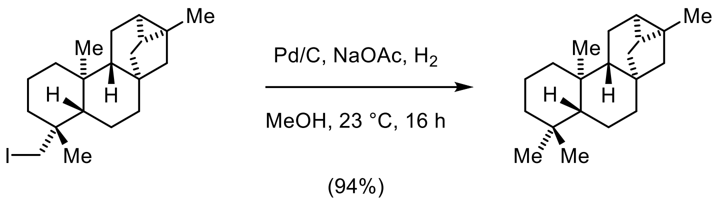

S-19 **5**

***ent*-Trachylobane 5.** A suspension of iodide **S-19** (9.3 mg, 23 µmol, 1 equiv), sodium acetate (3.8 mg, 47 µmol, 2.0 equiv) and palladium on charcoal (10 wt.%, 3.7 mg, 3.5 µmol, 0.15 equiv) in methanol (2 mL) was sparged with hydrogen for 15 min at 23 °C. After 16 h, the mixture was concentrated and the residue was purified by flash column chromatography on silica gel (100% *n*-pentane) to yield hydrocarbon **5** (6.0 mg, 22 µmol, 94%) as a colorless oil.

TLC (100% *n*-pentane): R_f = 0.87 (CAM)

$[\alpha]_D^{20}$ = –37.3 (c = 0.220 in dichloromethane)

^{1}**H-NMR** (400 MHz, $CDCl_3$): δ = 2.06 (d, *J* = 11.7 Hz, 1H), 1.86 (ddd, *J* = 14.4, 11.2, 3.1 Hz, 1H), 1.65 (ddd, *J* = 14.5, 7.2, 2.4 Hz, 1H), 1.51 – 1.42 (m, 3H), 1.41 – 1.27 (m, 6H), 1.25 – 1.21 (m, 2H), 1.15 (dd, *J* = 3.3, 1.8 Hz, 1H), 1.12 (s, 3H), 1.09 (d, *J* = 13.5 Hz, 2H), 0.93 (s, 3H), 0.82 (s, 3H), 0.79 (s, 3H), 0.75 – 0.72 (m, 1H), 0.68 (dd, *J* = 13.1, 3.1 Hz, 1H), 0.56 (dt, *J* = 7.3, 2.9 Hz, 1H) ppm.

^{13}**C-NMR** (101 MHz, $CDCl_3$): δ = 56.2, 53.4, 50.6, 42.2, 40.8, 39.3, 39.1, 38.4, 33.6, 33.5, 33.0, 24.3, 22.5, 21.7, 20.7, 20.6, 20.2, 19.8, 18.2, 14.6 ppm.

IR (ATR, neat): $\tilde{\nu}$ = 2922 (s), 2856 (m), 1462 (w), 1387 (w), 1367 (w), 844 (w).

HRMS (ESI): calcd for $C_{20}H_{33}$ $[M+H^+]^+$: 273.2577; found: 273.2578.

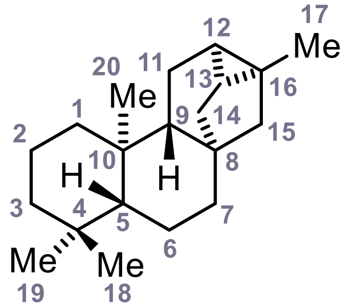

***ent*-trachylobane (5)**

Atom number	Natural **5**[10] (60 MHz, $CDCl_3$) δ_H [ppm]	Synthetic **5** (400 MHz, $CDCl_3$) δ_H [ppm]	$\lvert\Delta\delta_H\rvert$ [ppm]
17	1.10 (s, 3H)	1.12 (s, 3H)	0.02
18	0.82 (s, 3H)	0.82 (s, 3H)	0.00
19	0.78 (s, 3H)	0.79 (s, 3H)	0.01
20	0.93 (s, 3H)	0.93 (s, 3H)	0.00

Atom number	Semisynthetic **5**[11] (60 MHz, $CDCl_3$) δ_H [ppm]	Synthetic **5** (400 MHz, $CDCl_3$) δ_H [ppm]	$\lvert\Delta\delta_H\rvert$ [ppm]
17	1.12(5) (s, 3H)	1.12 (s, 3H)	0.00
18	0.82(5) (s, 3H)	0.82 (s, 3H)	0.00
19	0.80(0) (s, 3H)	0.79 (s, 3H)	0.01
20	0.94(0) (s, 3H)	0.93 (s, 3H)	0.01

Atom number	Natural **5**[5] (25.15 MHz, $CDCl_3$) δ_C [ppm]	Synthetic **5** (101 MHz, $CDCl_3$) δ_C [ppm]	$\lvert\Delta\delta_C\rvert$ [ppm]
1	39.2	39.3	0.1
2	18.3	18.2	0.1
3	42.2	42.2	0.0
4	33.0	33.0	0.0
5	56.2	56.2	0.0
6	20.2	20.2	0.0
7	39.1	39.1	0.0
8	40.8	40.8	0.0
9	53.4	53.4	0.0
10	38.4	38.4	0.0
11	19.7	19.8	0.1
12	20.7	20.7	0.0
13	24.4	24.3	0.1
14	33.5	33.5	0.0
15	50.6	50.6	0.0
16	22.5	22.5	0.0
17	20.7	20.6	0.1
18	33.5	33.6	0.1
19	21.7	21.7	0.0
20	14.6	14.6	0.0

Mechanistic Proposals

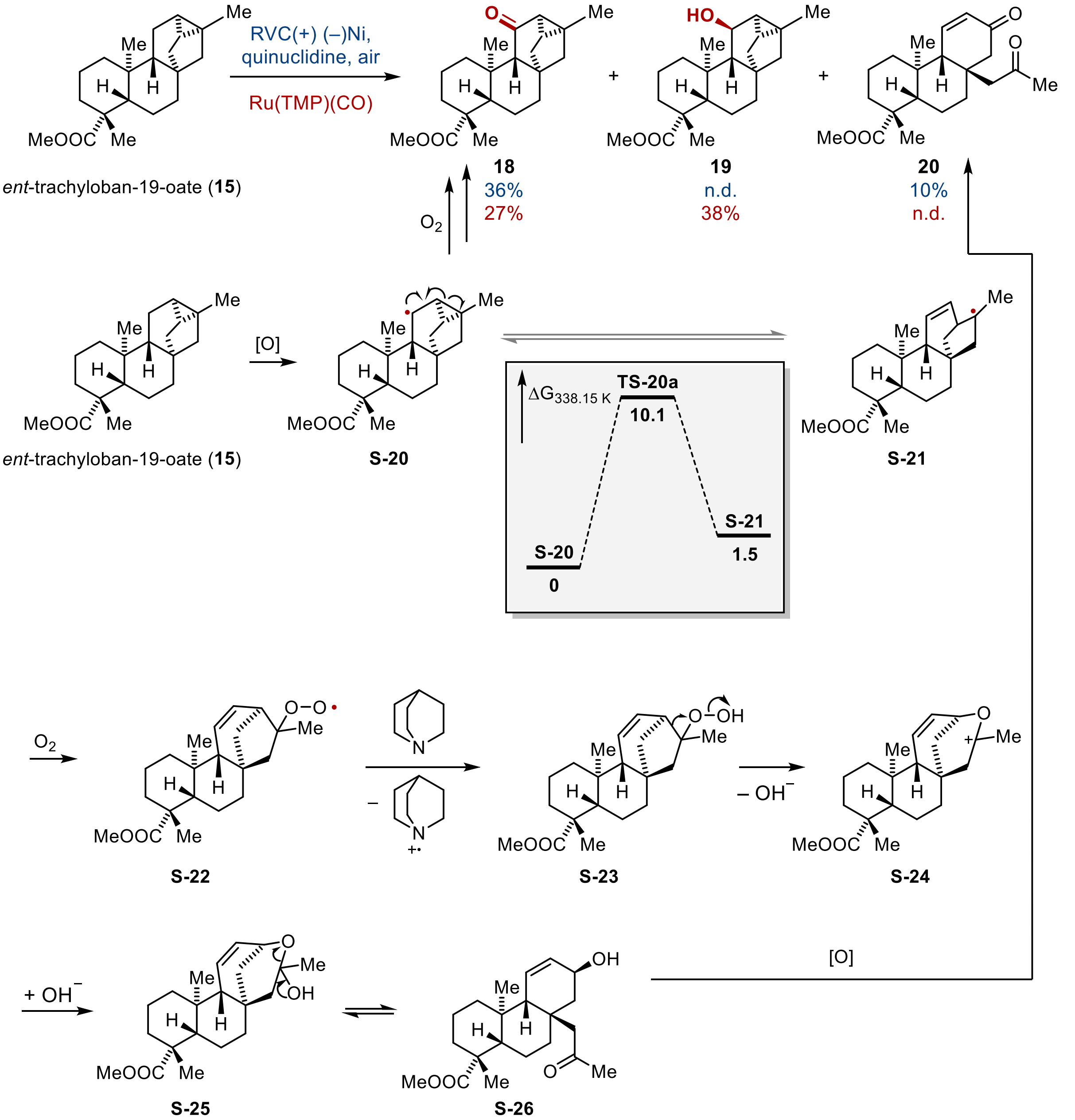

Scheme 1| Putative mechanistic scenario for C–H oxidations of methyl *ent*-trachyloban-19-oate **15**. Crucial equilibrium between radicals **S-20** and **S-21** calculated with uωB97xD/6-31+G(d,p) in dichloromethane treated as the implicit solvent. Relative Gibbs free energies at 338.15 K are given in kcal mol^{-1}.The energy of the respective radical **S-20** is arbitrarily set to zero.

For the ruthenium catalyted or electrochemical C–H oxidation of **15** the calculated equilibrium of the initial secondary C-11 radical **S-20** and the disfavored tertiary C-16 radical **S-21** (ΔG = +1.5 kcal mol^{-1}) reflect the observed experimental formations of **18**, **19** and minor **20** (in the electrochemical scenario) (Scheme 1). A pathway leading to the alternative secondary C-13 radical **S-27** could be excluded, as **S-27** is highly disfavored (ΔG = +4.8 kcal mol^{-1}). In addition, **TS-20b** is slightly higher in energy compared to **TS-20a** ($\Delta\Delta G$ = +0.3 kcal mol^{-1}) (Scheme 2).

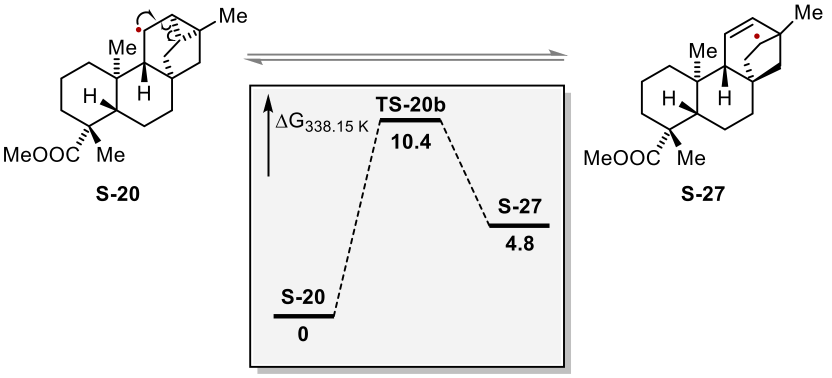

Scheme 2| Excluded alternative ring opening of radical **S-20** toward secondary radical **S-27**. Crucial equilibrium between radicals **S-20** and **S-27** calculated with uωB97xD/6-31+G(d,p) in dichloromethane treated as the implicit solvent. Relative Gibbs free energies at 338.15 K are given in kcal mol^{-1}.The energy of the respective radical **S-20** is arbitrarily set to zero.

For the manganese catalyted C–H oxidation of **6** the calculated equilibrium of the initial secondary C-15 radical **S-28** and the favored secondary C-13 radical **S-29** (ΔG = –2.9 kcal mol^{-1}) reflect the observed experimental formation of **22** (Scheme 3). A pathway leading to the alternative secondary C-12 radical **S-31** could be excluded, as **S-31** is highly disfavored (ΔG = +0.7 kcal mol^{-1}). In addition, **TS-28b** is slightly higher in energy compared to **TS-28a** ($\Delta\Delta G$ = +0.6 kcal mol^{-1}) (Scheme 4).

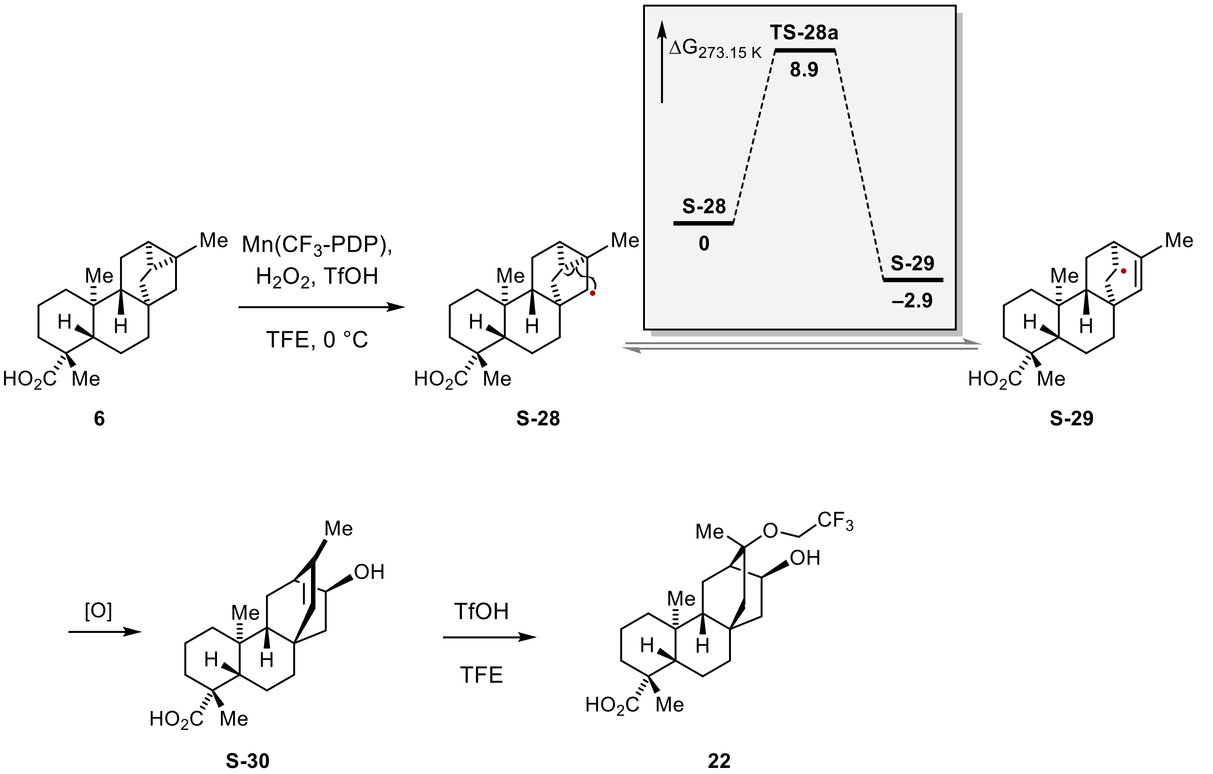

Scheme 3| Putative mechanistic scenario for the Mn(*R*,*R*-CF_3-PDP) catalyzed C–H oxidation of *ent*-trachyloban-19-oate **6**. Crucial equilibrium between radicals **S-28** and **S-29** calculated Calculated with uωB97xD/6-31+G(d,p) in 2,2,2-trifluoroethanol treated as the implicit solvent. Relative Gibbs free energies at 273.15 K are given in kcal mol^{-1}. The energy of the respective radical **S-28** is arbitrarily set to zero.

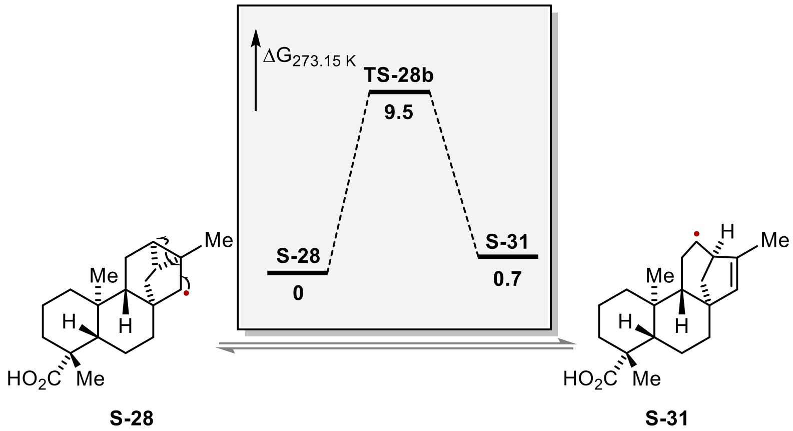

Scheme 4| Excluded alternative ring opening of radical **S-28** toward secondary radical **S-29**. Crucial equilibrium between radicals **S-28** and **S-31** calculated with uωB97xD/6-31+G(d,p) in 2,2,2-trifluoroethanol treated as the implicit solvent. Relative Gibbs free energies at 273.15 K are given in kcal mol^{-1}. The energy of the respective radical **S-28** is arbitrarily set to zero.

Computational Studies

Computational Methodology

All calculations were carried out with the Gaussian 16 package.[12] Investigated structures were fully optimized in implicit solvent with the uωB97xD hybrid density functional[13] and the 6-31G+(d,p)[14] basis set on all atoms. Bulk solvent effects were implicitly modelled by Integral Equation Formalism Polarizable Continuum Model (IEFPCM) of Tomasi and Pascual-Ahuir[15-17] as implemented in Gaussian 16. The internally stored parameters for dichloromethane ($\varepsilon = 8.93$) and 2,2,2-trifluoroethanol ($\varepsilon = 26.726$) were used. All species were calculated considering a open shell electronic configurations (doublet). Frequency analyses were carried out at the same level as the geometry optimizations and the nature of the stationary points was determined by analyses of the Hessian matrix. (Local) energy minima were confirmed to show only real eigenvalues, whereas transition states were confirmed to have one imaginary eigenvalue, while the corresponding eigenvector coincided with the change in the reaction coordinate. Zero-point energy and thermal corrections were calculated using the standard rigid-rotator/harmonic oscillator model to obtain Gibbs free energies at 298.15 K, 273.15 K or 338.15 K, respectively, no scaling of the frequencies was applied. The possibility of different conformations was considered for all structures. Structures were visualized by using CylView.[18]

Cartesian Coordinates

C-11 Radical S-20

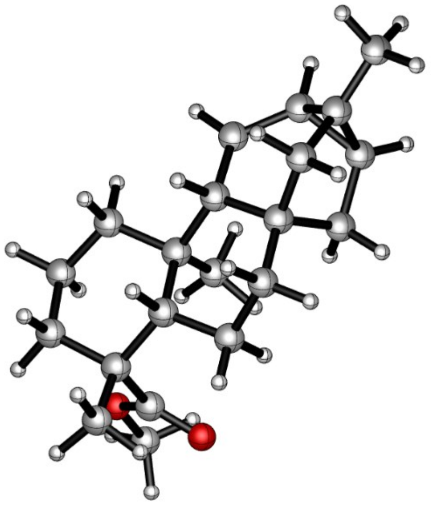

IEFPCM(CH_2Cl_2)uωB97xD/6-31+G(d,p) Electronic Energy = –969.118843

IEFPCM(CH_2Cl_2)uωB97xD/6-31+G(d,p) Free Energy (338.15 K) = –968.690555

Number of imaginary frequencies = 0

C	2.211511	2.014979	-1.257898	H	2.583939	2.365839	-0.290994
C	2.909582	0.711282	-1.637997	H	2.649621	0.465585	-2.676622
C	2.502872	-0.512159	-0.789672	H	3.996133	0.837260	-1.608725
C	0.942214	-0.617254	-0.796909	H	0.712319	-0.714825	-1.871280
C	0.209041	0.679647	-0.341228	H	0.335551	1.683344	-2.251621
C	0.692097	1.852756	-1.225569	H	0.234580	2.787743	-0.882782
C	0.351038	-1.866815	-0.130506	H	0.903056	-2.757535	-0.442247
C	-1.117176	-2.034133	-0.516771	H	0.453549	-1.812322	0.957316
C	-1.972801	-0.834230	-0.124425	H	-1.183660	-2.171561	-1.606006
C	-1.322918	0.482420	-0.632659	H	-1.530092	-2.942388	-0.060208
C	-3.394257	-0.903311	-0.733179	H	-1.366401	0.407290	-1.736597
C	-4.216925	0.052122	0.113821	H	-3.798407	-1.920158	-0.627856
C	-3.450036	1.337521	0.468032	H	-3.397667	-0.650180	-1.800786
C	-2.190296	1.627819	-0.209923	H	-4.074634	2.187312	0.729936
C	-2.300647	-0.754541	1.389584	H	-1.956421	2.646836	-0.499218
C	-3.518293	0.146187	1.435280	H	-1.474320	-0.388570	1.999386
C	-5.714598	0.083232	-0.039552	H	-2.567253	-1.754618	1.755878
C	0.478150	1.031995	1.134507	H	-4.074216	0.272881	2.360257
C	3.069678	-0.481931	0.630471	H	-6.001449	0.399630	-1.048715
O	2.871684	-1.346688	1.462727	H	-6.141540	-0.911478	0.136617
O	3.864352	0.567073	0.874923	H	-6.170653	0.776834	0.674147
C	4.424472	0.648274	2.189549	H	0.458828	0.159914	1.790320
C	3.125739	-1.764043	-1.446642	H	1.454654	1.505756	1.262889
H	2.475420	2.789135	-1.987125	H	-0.277283	1.740366	1.489700

H	5.032195	1.551257	2.192059	H	4.198405	-1.612035	-1.606619
H	3.628747	0.721384	2.934352	H	2.998622	-2.657859	-0.833820
H	5.040170	-0.228524	2.398788	H	2.659235	-1.938296	-2.421529

Ring Opening Transition State TS-20a

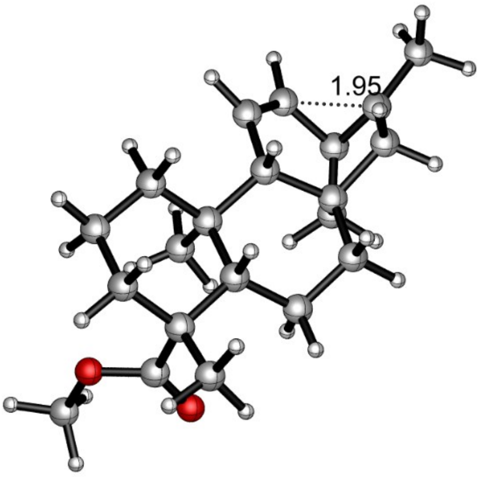

IEFPCM(CH_2Cl_2)uωB97xD/6-31+G(d,p) Electronic Energy = –969.102675

IEFPCM(CH_2Cl_2)uωB97xD/6-31+G(d,p) Free Energy (338.15 K) = –968.674387

Number of imaginary frequencies = 1 (550.40 i cm^{-1})

C	-2.218454	-1.962203	-1.326784	O	-3.866116	-0.583713	0.856410
C	-2.911703	-0.643130	-1.660699	C	-4.425137	-0.707762	2.168165
C	-2.500987	0.548590	-0.770214	C	-3.119361	1.824811	-1.383057
C	-0.939993	0.649100	-0.773238	H	-2.486186	-2.709302	-2.082432
C	-0.210355	-0.665166	-0.367847	H	-2.591370	-2.345175	-0.372365
C	-0.698613	-1.806054	-1.290397	H	-2.650527	-0.362206	-2.690085
C	-0.344412	1.867197	-0.055337	H	-3.998796	-0.765731	-1.636001
C	1.124152	2.044295	-0.434056	H	-0.709315	0.789158	-1.842783
C	1.981827	0.825570	-0.104627	H	-0.343202	-1.603578	-2.310977
C	1.322148	-0.467443	-0.659519	H	-0.240047	-2.751338	-0.978124
C	3.405872	0.950935	-0.717086	H	-0.891549	2.773285	-0.329305
C	4.290370	0.094498	0.148187	H	-0.447747	1.767995	1.029117
C	3.255600	-1.513346	0.554396	H	1.189591	2.235606	-1.515290
C	2.185356	-1.651301	-0.305572	H	1.541625	2.927621	0.065926
C	2.319040	0.667774	1.397024	H	1.364473	-0.356078	-1.757012
C	3.512223	-0.273877	1.362476	H	3.739648	2.000133	-0.656956
C	5.764854	-0.029084	-0.025814	H	3.426029	0.671385	-1.779134
C	-0.480110	-1.069138	1.093863	H	3.942593	-2.342745	0.699997
C	-3.068296	0.470737	0.647835	H	2.089095	-2.566206	-0.883181
O	-2.868317	1.305809	1.509404	H	1.496007	0.284172	1.999442

H	2.624082	1.636368	1.812762	H	0.265307	-1.801596	1.418657
H	4.052771	-0.430941	2.296446	H	-5.034696	-1.609109	2.141132
H	6.182974	-0.793429	0.636494	H	-3.628782	-0.807194	2.909272
H	6.034011	-0.286333	-1.056950	H	-5.038668	0.162884	2.407326
H	6.268737	0.921654	0.210952	H	-4.192843	1.683016	-1.547057
H	-0.447468	-0.223453	1.783117	H	-2.987873	2.696408	-0.739908
H	-1.463544	-1.532626	1.206074	H	-2.652989	2.030580	-2.351852

C-16 Radical S-21

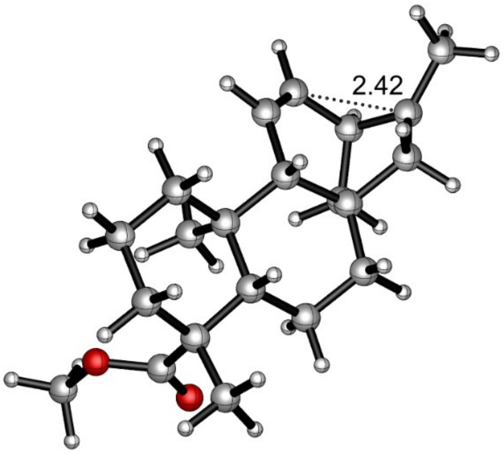

IEFPCM(CH_2Cl_2)uωB97xD/6-31+G(d,p) Electronic Energy = –969.115435

IEFPCM(CH_2Cl_2)uωB97xD/6-31+G(d,p) Free Energy (338.15 K) = –968.688146

Number of imaginary frequencies = 0

C	2.213942	1.858445	-1.460527	H	-1.427259	0.333362	-1.730866
C	2.887998	0.514949	-1.723241	C	-5.760227	0.044351	-0.221855
C	2.482805	-0.608171	-0.746767	C	0.501205	1.143774	1.038899
C	0.920661	-0.694204	-0.711010	C	3.083562	-0.442116	0.650148
C	0.202826	0.650308	-0.389769	O	2.885757	-1.207403	1.574325
C	0.693528	1.720620	-1.395113	O	3.909488	0.605489	0.763668
C	0.336301	-1.847495	0.111761	C	4.505236	0.808071	2.049364
C	-1.129383	-2.051859	-0.249254	C	3.075191	-1.929988	-1.285227
C	-2.005445	-0.819537	-0.028129	H	2.476573	2.556205	-2.263508
C	-1.343390	0.452719	-0.638519	H	2.603121	2.294752	-0.535883
C	-3.407910	-1.046890	-0.672626	H	2.601881	0.170582	-2.726382
C	-4.393726	-0.344886	0.219218	H	3.976446	0.626580	-1.728572
C	-3.134741	1.680072	0.622663	H	0.323992	1.452446	-2.395021
C	-2.133508	1.688563	-0.263727	H	0.256657	2.694195	-1.147896
C	-2.391197	-0.547250	1.443728	H	0.881692	-2.773298	-0.089135
C	-3.596995	0.385904	1.270674	H	0.445835	-1.658511	1.183214
H	0.666676	-0.914114	-1.761588	H	-1.189985	-2.332617	-1.311250

H	-1.550142	-2.891748	0.318243
H	-3.442134	-0.681399	-1.711352
H	-3.625822	-2.125221	-0.725759
H	-3.693541	2.590717	0.827412
H	-1.865913	2.623977	-0.749170
H	-2.702684	-1.480598	1.928290
H	-1.592776	-0.101443	2.038331
H	-4.153028	0.579989	2.193220
H	-6.386851	0.348994	0.623361
H	-5.731979	0.891869	-0.928876
H	-6.261194	-0.780541	-0.741521
H	0.407974	0.355836	1.788284
H	1.514245	1.545075	1.119477
H	-0.186715	1.948570	1.312443
H	5.136267	1.688466	1.942578
H	3.731218	0.981633	2.800406
H	5.102691	-0.059439	2.335851
H	4.146322	-1.811088	-1.479643
H	2.948903	-2.756265	-0.583878
H	2.587177	-2.193940	-2.228906

Ring Opening Transition State TS-20b

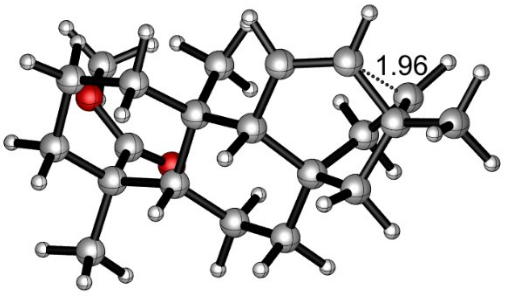

IEFPCM(CH_2Cl_2)uωB97xD/6-31+G(d,p) Electronic Energy = – 969.101572

IEFPCM(CH_2Cl_2)uωB97xD/6-31+G(d,p) Free Energy (338.15 K) = –968.674001

Number of imaginary frequencies = 1 (559.67 i cm^{-1})

C	2.205605	2.113129	-1.090983
C	2.906541	0.845688	-1.573794
C	2.499954	-0.442516	-0.827750
C	0.939346	-0.547406	-0.844423
C	0.202016	0.707679	-0.284443
C	0.686404	1.946623	-1.075005
C	0.350834	-1.850354	-0.286784
C	-1.117508	-1.984282	-0.684365
C	-1.969609	-0.825328	-0.177218
C	-1.324107	0.528075	-0.593584
C	-3.394782	-0.840245	-0.771914
C	-4.178813	0.079317	0.158113
C	-3.486687	1.404320	0.325245
C	-2.193434	1.649615	-0.084788
C	-2.278885	-0.897052	1.348794
C	-3.583864	-0.168169	1.497778
C	-5.682835	0.116433	-0.008323
C	0.477077	0.935933	1.215143
C	3.066690	-0.528564	0.590078
O	2.868218	-1.458444	1.348656
O	3.860978	0.497006	0.919960
C	4.420606	0.469663	2.237111
C	3.122943	-1.636617	-1.584873
H	2.469032	2.944110	-1.754836
H	2.575528	2.385624	-0.098157
H	2.648424	0.684432	-2.629290
H	3.992866	0.970473	-1.533043
H	0.710681	-0.555379	-1.923299
H	0.332216	1.854885	-2.111565
H	0.228940	2.854774	-0.666929
H	0.904326	-2.709604	-0.674703
H	0.454325	-1.889574	0.801827

H	-1.187199	-2.017502	-1.781458	H	-6.103148	-0.888561	0.106437
H	-1.528911	-2.931850	-0.314750	H	-6.145573	0.766137	0.742054
H	-1.365213	0.524670	-1.698225	H	0.469820	0.009460	1.791922
H	-3.817015	-1.853057	-0.724487	H	1.451139	1.404244	1.376524
H	-3.414991	-0.508377	-1.816544	H	-0.276076	1.604640	1.642773
H	-4.078129	2.206509	0.760552	H	5.025261	1.371298	2.315472
H	-1.772493	2.637176	0.075649	H	3.624460	0.477174	2.984963
H	-1.486511	-0.492324	1.980173	H	5.039246	-0.419522	2.372313
H	-2.402354	-1.952866	1.636033	H	4.195098	-1.470628	-1.733856
H	-4.080622	0.044408	2.437450	H	2.998056	-2.577410	-1.046401
H	-5.954745	0.494489	-0.999660	H	2.655045	-1.732015	-2.569938

C-13 Radical S-27

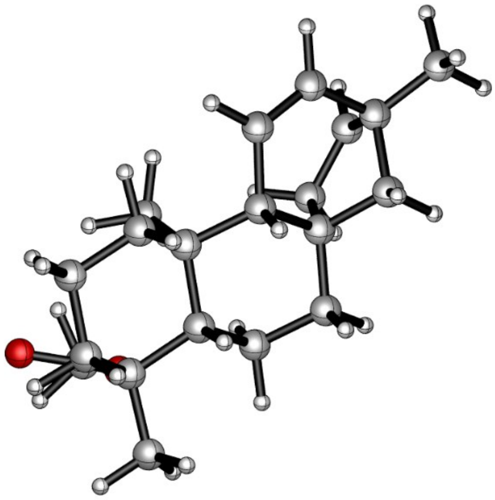

IEFPCM(CH_2Cl_2)uωB97xD/6-31+G(d,p) Electronic Energy = –969.111821

IEFPCM(CH_2Cl_2)uωB97xD/6-31+G(d,p) Free Energy (338.15 K) = –968.682930

Number of imaginary frequencies = 0

C	-2.161633	2.536059	-0.426897	C	3.300763	-0.278957	1.158612
C	-2.929998	1.803484	0.669046	C	4.068834	-0.117974	-0.163743
C	-2.585016	0.305541	0.775649	C	3.510346	1.100668	-0.892337
C	-1.034532	0.175032	0.928701	C	2.241495	1.498449	-0.741159
C	-0.215616	0.854679	-0.214062	C	-3.179465	-0.439970	-0.419539
C	-0.651050	2.338835	-0.296017	C	2.122608	-1.562917	-0.487659
C	-0.494940	-1.219277	1.280766	C	3.526144	-1.286555	-0.940248
C	0.954705	-1.127546	1.755538	H	-0.836183	0.792177	1.820947
C	1.862240	-0.549748	0.673918	O	-3.957455	0.033363	-1.225830
C	1.287995	0.823497	0.221549	H	1.298904	1.436433	1.139781

C	-0.441221	0.197866	-1.589843	H	4.165235	1.606298	-1.600645
C	-3.301882	-0.258545	2.023230	H	1.889464	2.358862	-1.304156
C	5.584794	-0.063263	-0.024785	H	2.027801	-2.588234	-0.096502
O	-2.806101	-1.723885	-0.462735	H	1.405022	-1.491372	-1.307781
C	-3.314296	-2.502348	-1.550010	H	3.924301	-1.598346	-1.902207
H	-2.388604	3.606614	-0.375102	H	-0.482576	-0.891395	-1.535956
H	-2.511948	2.197442	-1.407016	H	-1.377822	0.530074	-2.044386
H	-2.690496	2.255830	1.640611	H	0.365190	0.472362	-2.277719
H	-4.007392	1.920275	0.518803	H	-4.363784	0.006645	1.998005
H	-0.306265	2.852502	0.612614	H	-3.225542	-1.344982	2.094604
H	-0.156616	2.829527	-1.141363	H	-2.864494	0.180131	2.925963
H	-1.103665	-1.667473	2.070714	H	5.955694	-0.957253	0.486811
H	-0.562607	-1.893232	0.421708	H	6.066245	-0.008537	-1.007580
H	1.004230	-0.487014	2.648027	H	5.891708	0.816533	0.551450
H	1.321996	-2.116823	2.055403	H	-2.894989	-3.497939	-1.416448
H	3.370938	0.603976	1.804388	H	-4.404899	-2.539538	-1.518635
H	3.688347	-1.144342	1.712638	H	-2.990402	-2.077079	-2.502695

C-15 Radical S-28

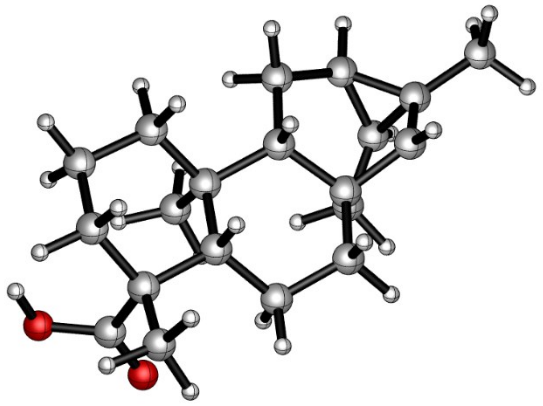

IEFPCM(TFE)uωB97xD/6-31+G(d,p) Electronic Energy = –929.814615

IEFPCM(TFE)uωB97xD/6-31+G(d,p) Free Energy (273.15 K) = –929.395439

Number of imaginary frequencies = 0

C	3.152646	1.063160	-1.132366	C	-3.052664	-0.799746	-0.981985
C	2.763042	-0.312352	-0.546592	C	-4.026659	-0.066842	-0.169204
C	1.208216	-0.452937	-0.652796	C	-3.387284	1.155645	0.513780
C	0.404348	0.720300	-0.012753	C	-2.034491	1.674439	0.058107
C	0.624876	-1.814253	-0.249460	C	-2.226338	-0.993633	1.223321
C	-0.812571	-1.947599	-0.745865	C	-3.484742	-0.142825	1.257686
C	-1.739243	-0.841283	-0.242042	H	1.035469	-0.363515	-1.737980
C	-1.086534	0.561103	-0.466198	H	-1.042123	0.664115	-1.560083

C	0.901817	2.057694	-0.610554	H	-1.488161	-0.684290	1.964855
C	2.413707	2.253344	-0.519248	H	-2.479358	-2.042915	1.418870
C	0.587477	0.760870	1.517057	H	-4.170955	-0.224593	2.096032
C	3.286310	-0.555544	0.878088	H	0.614016	2.092544	-1.670425
O	2.985968	-1.520943	1.546288	H	0.392960	2.894775	-0.119345
O	4.189155	0.302779	1.391376	H	2.712201	2.413515	0.523628
C	3.458544	-1.396670	-1.402727	H	2.698299	3.165221	-1.053731
C	-5.497394	-0.063567	-0.503461	H	0.514628	-0.224503	1.977981
H	4.241688	1.208034	-1.105910	H	1.566199	1.164120	1.789917
H	2.909067	1.023061	-2.201136	H	-0.154341	1.408133	1.991576
H	1.221285	-2.623073	-0.680194	H	4.391514	1.018032	0.776008
H	0.668676	-1.951213	0.833841	H	4.533382	-1.198952	-1.469749
H	-0.807824	-1.917002	-1.844841	H	3.323386	-2.396405	-0.987308
H	-1.223636	-2.923472	-0.461360	H	3.046917	-1.380862	-2.416614
H	-3.120037	-0.905385	-2.062740	H	-5.675875	0.391177	-1.484288
H	-4.097586	1.925676	0.808342	H	-5.896416	-1.083475	-0.525199
H	-1.582595	2.204264	0.903524	H	-6.066070	0.506025	0.238288
H	-2.184083	2.430465	-0.720002				

Ring Opening Transition State TS-28a

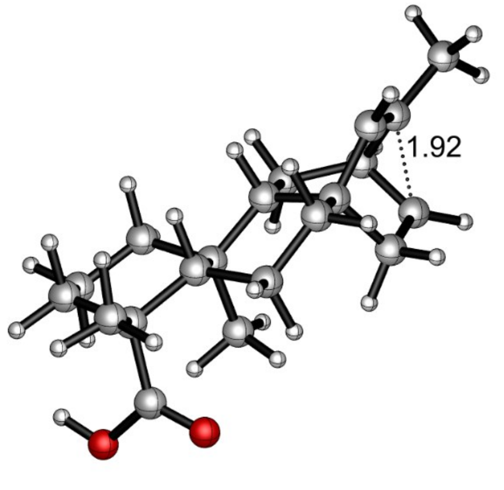

IEFPCM(TFE)uωB97xD/6-31+G(d,p) Electronic Energy = –929.798272

IEFPCM(TFE)uωB97xD/6-31+G(d,p) Free Energy (273.15 K) = –929.381302

Number of imaginary frequencies = 1 (598.27 i cm^{-1})

C	3.122345	1.078721	-1.158757	C	-1.744996	-0.855424	-0.212192
C	2.749417	-0.299780	-0.568788	C	-1.102079	0.547702	-0.437988
C	1.194377	-0.450063	-0.654749	C	-3.080014	-0.857002	-0.920543
C	0.391799	0.716874	-0.002064	C	-3.995227	0.013159	-0.342209
C	0.623950	-1.815997	-0.247516	C	-3.396578	1.069145	0.545596
C	-0.819023	-1.956359	-0.726368	C	-2.054393	1.646282	0.100719

C	-2.176550	-1.022913	1.280325	H	-1.617805	2.172372	0.955858
C	-3.347897	-0.099249	1.462698	H	-2.230695	2.403841	-0.669647
H	1.006752	-0.359173	-1.737298	H	-1.365654	-0.800176	1.979410
H	-1.073247	0.652021	-1.531127	H	-2.475049	-2.064361	1.453417
C	0.871185	2.058009	-0.606684	H	-4.106507	-0.253664	2.221482
C	2.382843	2.264733	-0.537732	H	0.567540	2.091098	-1.662104
C	0.597683	0.761317	1.525263	H	0.363080	2.891027	-0.107530
C	3.292120	-0.540845	0.848904	H	2.695281	2.430368	0.500197
O	3.012087	-1.512922	1.516344	H	2.653042	3.177267	-1.078545
O	4.188574	0.327676	1.355942	H	0.567020	-0.226065	1.986366
C	3.440705	-1.378677	-1.435000	H	1.565888	1.197763	1.784298
C	-5.466716	0.038363	-0.652178	H	-0.159278	1.380795	2.012974
H	4.210644	1.230908	-1.144762	H	4.374217	1.048250	0.741572
H	2.866425	1.036787	-2.224543	H	4.513231	-1.173705	-1.516226
H	1.219473	-2.620134	-0.688088	H	3.317765	-2.379549	-1.018529
H	0.684030	-1.955826	0.834762	H	3.015479	-1.364932	-2.443289
H	-0.828735	-1.924108	-1.825143	H	-5.699719	0.846344	-1.355839
H	-1.220367	-2.935821	-0.438384	H	-5.797527	-0.907056	-1.091762
H	-3.379504	-1.674308	-1.571945	H	-6.054823	0.217585	0.253253
H	-4.113969	1.836428	0.838457				

C-13 Radical S-29

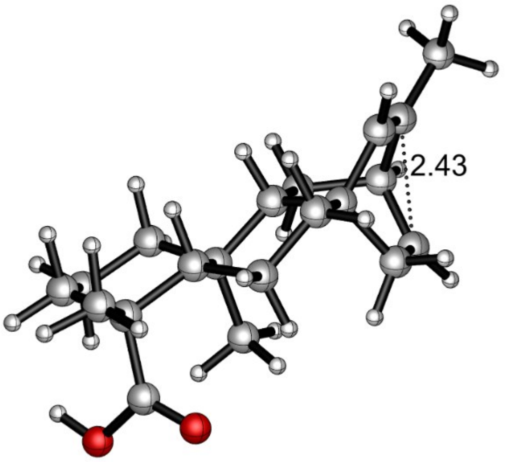

IEFPCM(TFE)uωB97xD/6-31+G(d,p) Electronic Energy = –929.817344

IEFPCM(TFE)uωB97xD/6-31+G(d,p) Free Energy (273.15 K) = –929.400139

Number of imaginary frequencies = 0

C	3.048953	1.143976	-1.175756	C	0.629165	-1.828694	-0.311999
C	2.713343	-0.253320	-0.607665	C	-0.826280	-1.990150	-0.750602
C	1.160960	-0.436015	-0.681062	C	-1.754452	-0.927102	-0.164364
C	0.343313	0.697461	0.012947	C	-1.149978	0.498262	-0.398713

C	-3.107215	-0.909412	-0.852184	H	-3.333538	-1.612348	-1.652418
C	-3.974094	0.009714	-0.412053	H	-4.125275	1.613657	1.060621
C	-3.403712	0.872512	0.707113	H	-2.413295	2.272118	-0.640374
C	-2.130379	1.568656	0.150128	H	-1.681017	2.160613	0.952851
C	-2.063482	-1.158035	1.341646	H	-2.551639	-2.141381	1.444373
C	-2.961969	-0.057342	1.801346	H	-1.152881	-1.217958	1.947115
H	0.958564	-0.316904	-1.758252	H	-3.341146	0.006950	2.815440
H	-1.138664	0.600433	-1.492072	H	0.459842	2.101665	-1.627028
C	0.781061	2.059210	-0.577122	H	0.261807	2.871950	-0.056870
C	2.288121	2.301500	-0.527166	H	2.610352	2.459879	0.508952
C	0.565605	0.720101	1.538992	H	2.528274	3.228553	-1.057355
C	3.274515	-0.506831	0.800838	H	0.622648	-0.278174	1.972878
O	3.031679	-1.502340	1.448234	H	1.496857	1.229177	1.801437
O	4.144992	0.379480	1.321547	H	-0.239665	1.255837	2.048715
C	3.420880	-1.299840	-1.499668	H	4.301543	1.120018	0.723115
C	-5.367731	0.237824	-0.909080	H	4.486409	-1.065177	-1.591026
H	4.133614	1.320580	-1.167792	H	3.329909	-2.309798	-1.097277
H	2.784455	1.114996	-2.239843	H	2.982283	-1.281718	-2.502162
H	1.228470	-2.600435	-0.802594	H	-5.627797	-0.461043	-1.709382
H	0.728667	-2.009708	0.760975	H	-6.094081	0.119724	-0.096125
H	-0.873530	-1.924648	-1.846925	H	-5.481755	1.259777	-1.290507
H	-1.192526	-2.988588	-0.480165				

Ring Opening Transition State TS-28b

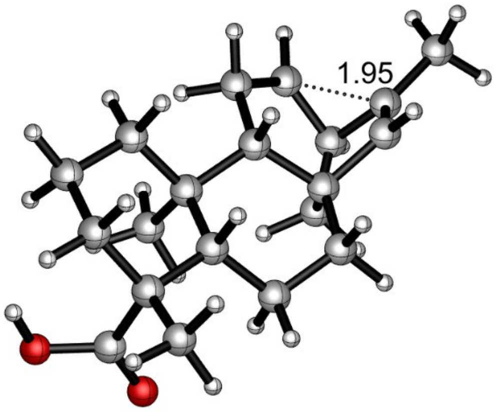

IEFPCM(TFE)uωB97xD/6-31+G(d,p) Electronic Energy = –929.796933

IEFPCM(TFE)uωB97xD/6-31+G(d,p) Free Energy (273.15 K) = –929.38029

Number of imaginary frequencies = 1 (607.46 i cm^{-1})

C	3.169854	1.000618	-1.166723	C	0.605529	-1.812487	-0.190537
C	2.760684	-0.349540	-0.537642	C	-0.831012	-1.941560	-0.686792
C	1.203586	-0.472726	-0.638875	C	-1.748355	-0.808395	-0.227347
C	0.413481	0.730109	-0.037203	C	-1.084181	0.579430	-0.480906

C	-3.069680	-0.807087	-0.976030
C	-4.071288	-0.285987	-0.182244
C	-3.301513	1.304238	0.652881
C	-1.999700	1.733834	0.034131
C	-2.244055	-0.904410	1.236486
C	-3.513189	-0.060165	1.200340
H	1.030988	-0.417031	-1.726371
H	-1.045431	0.667554	-1.575942
C	0.931973	2.039406	-0.679468
C	2.445958	2.218381	-0.592117
C	0.598709	0.815278	1.490869
C	3.281327	-0.554087	0.894196
O	2.965132	-1.490809	1.595029
O	4.199686	0.305246	1.377461
C	3.439999	-1.470774	-1.358766
C	-5.523825	-0.137720	-0.530047
H	4.260731	1.132485	-1.143184
H	2.926933	0.929894	-2.234058
H	1.193056	-2.642651	-0.591933
H	0.645354	-1.913103	0.896874
H	-0.821007	-1.949905	-1.786314
H	-1.259441	-2.900696	-0.371478
H	-3.131800	-0.939096	-2.053245
H	-4.075266	2.052492	0.792429
H	-1.461116	2.334600	0.779876
H	-2.206996	2.430725	-0.785459
H	-1.524632	-0.552001	1.976604
H	-2.503067	-1.943310	1.473494
H	-4.197733	-0.150875	2.044606
H	0.646637	2.040834	-1.740582
H	0.436207	2.902008	-0.220527
H	2.745870	2.407196	0.445538
H	2.742204	3.109516	-1.154594
H	0.509759	-0.154490	1.981359
H	1.583810	1.210702	1.751936
H	-0.132290	1.488523	1.946025
H	4.415504	0.993719	0.736641
H	4.517036	-1.289197	-1.435884
H	3.293576	-2.454258	-0.909688
H	3.025208	-1.483887	-2.371407
H	-5.662291	0.043499	-1.599937
H	-6.068081	-1.053081	-0.268492
H	-5.988068	0.686192	0.018566

C-12 Radical S-31

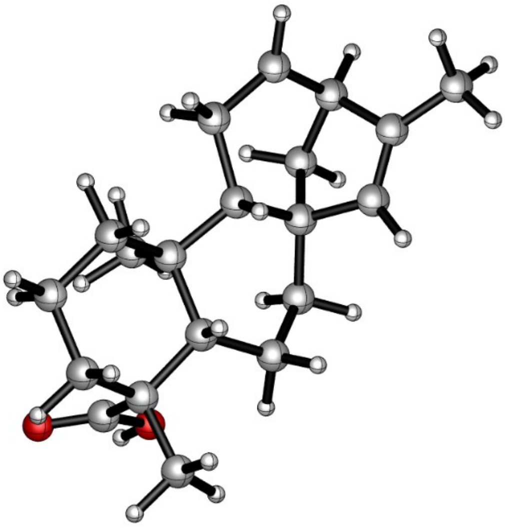

IEFPCM(TFE)uωB97xD/6-31+G(d,p) Electronic Energy = –929.810443

IEFPCM(TFE)uωB97xD/6-31+G(d,p) Free Energy (273.15 K) = –929.39436

Number of imaginary frequencies = 0

C	2.488432	2.227227	-0.564615
C	3.148242	1.029685	-1.244683
C	2.703104	-0.333781	-0.678859

C	1.144002	-0.383004	-0.700863	H	4.237616	1.112401	-1.188547
C	0.451073	0.786944	0.067896	H	0.591951	2.178667	-1.583840
C	0.962933	2.109555	-0.551033	H	0.540357	2.961779	-0.007175
C	0.504664	-1.782674	-0.467195	H	0.155364	-2.161954	-1.434520
C	-0.665756	-1.798993	0.515472	H	1.247067	-2.497924	-0.112241
C	-1.705009	-0.701922	0.263240	H	-1.167568	-2.772905	0.460218
C	-1.067889	0.642790	-0.242780	H	-0.287148	-1.722127	1.538655
C	-2.765883	-1.110435	-0.752300	H	-2.530131	-1.676186	-1.651199
C	-3.960629	-0.596507	-0.444462	H	-4.114002	2.323142	-0.014935
C	-3.380468	1.617997	0.365660	H	-1.515397	2.261352	1.141985
C	-1.913924	1.880207	0.187476	H	-1.763480	2.693020	-0.529405
C	3.338756	-0.551586	0.691137	H	-2.902923	-1.378591	1.945800
C	-2.602798	-0.428915	1.488628	H	-2.114292	0.184513	2.253118
C	-3.806836	0.255451	0.825322	H	-4.716203	0.280333	1.436576
H	0.932563	-0.143504	-1.752735	H	0.519810	-0.105719	2.093695
O	2.835838	-1.600488	1.361521	H	1.782506	1.074390	1.788840
O	4.261362	0.096835	1.147322	H	0.143576	1.615446	2.058077
H	-1.122457	0.605770	-1.340286	H	4.368476	-1.316724	-1.691969
C	0.738361	0.829741	1.582357	H	3.089257	-2.443843	-1.211405
C	3.285404	-1.439181	-1.590855	H	2.839267	-1.355061	-2.586690
C	-5.243969	-0.666349	-1.205575	H	-5.140085	-1.262323	-2.116524
H	2.775932	3.143749	-1.091432	H	-6.038000	-1.105919	-0.590494
H	2.873889	2.329342	0.455463	H	-5.580040	0.338524	-1.488078
H	2.881319	1.032517	-2.309464	H	3.324320	-1.680433	2.193962

NMR Spectra

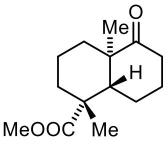

10

^{1}H NMR, CDCl$_3$, 400 MHz

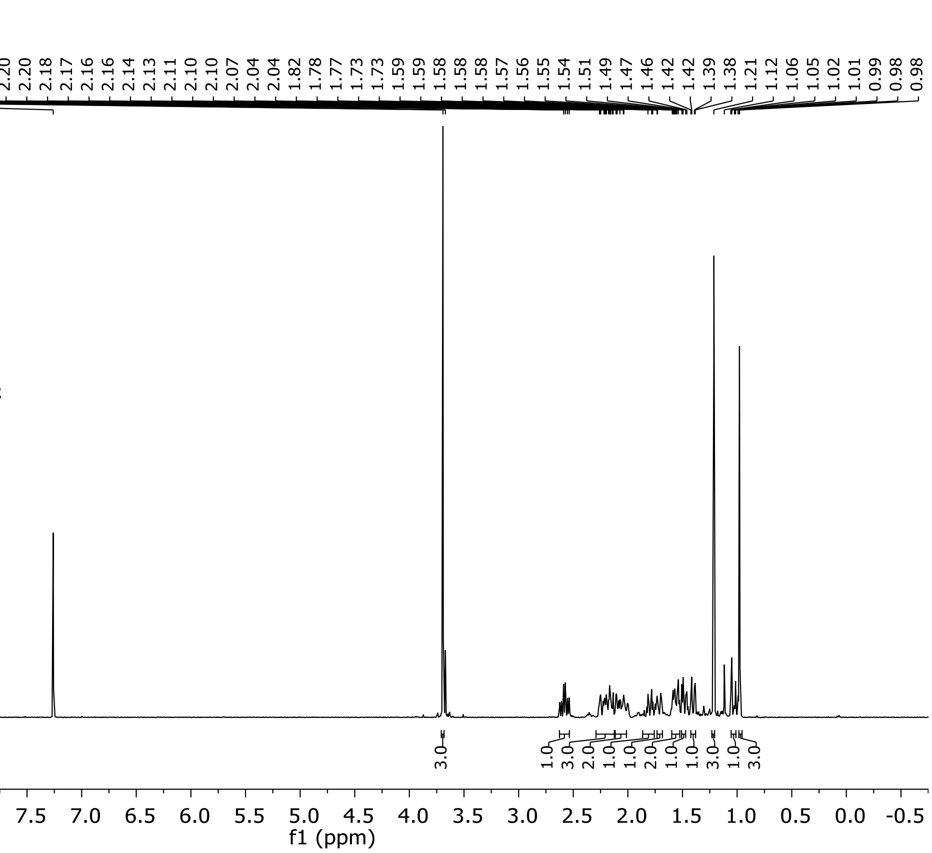

^{13}C NMR, CDCl$_3$, 101 MHz

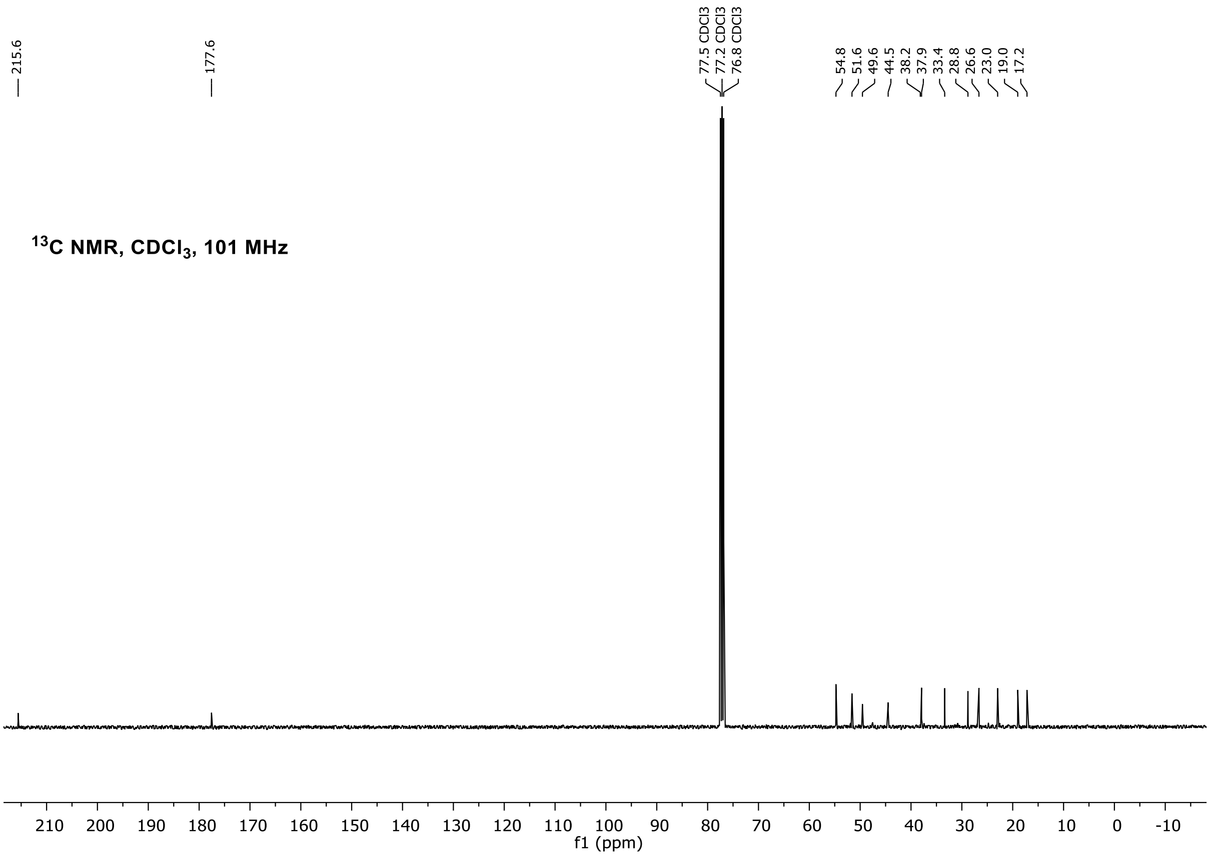

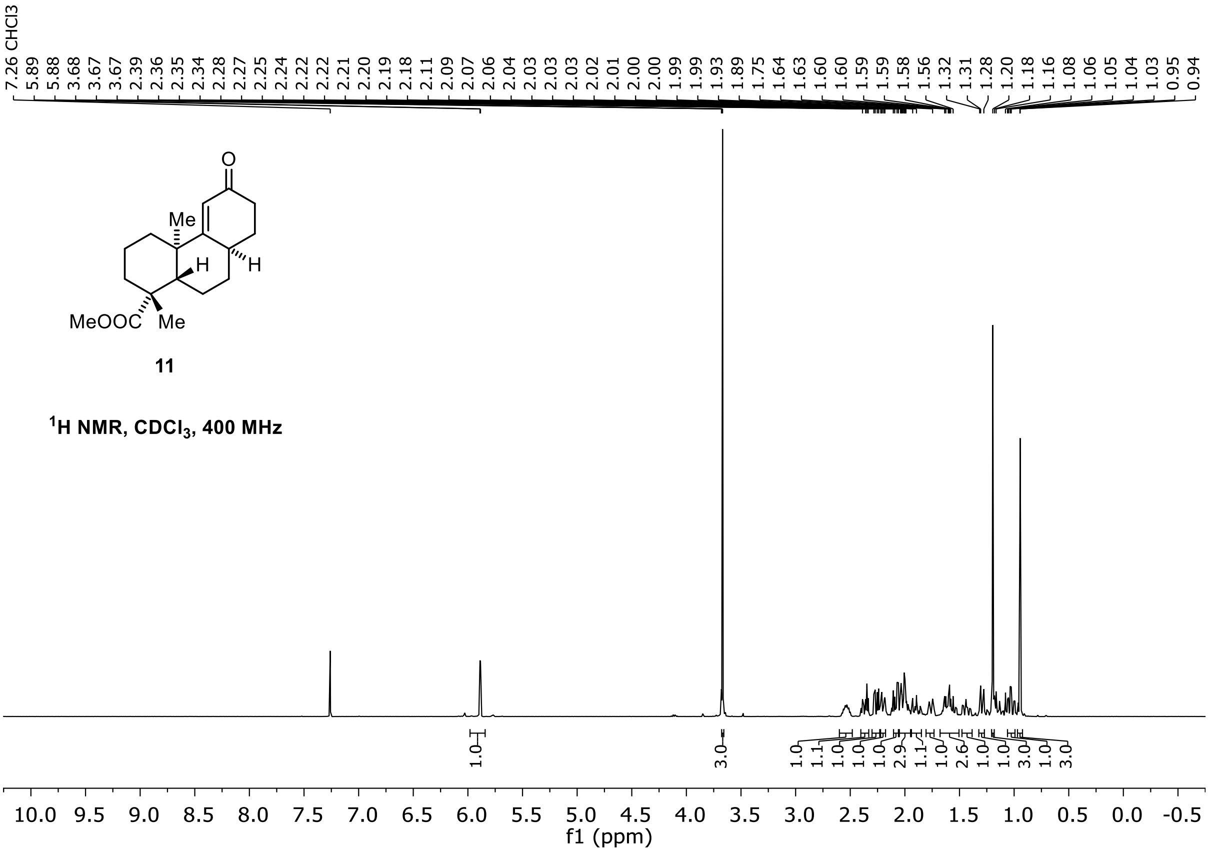

11

^{1}H NMR, $CDCl_3$, 400 MHz

^{13}C NMR, $CDCl_3$, 101 MHz

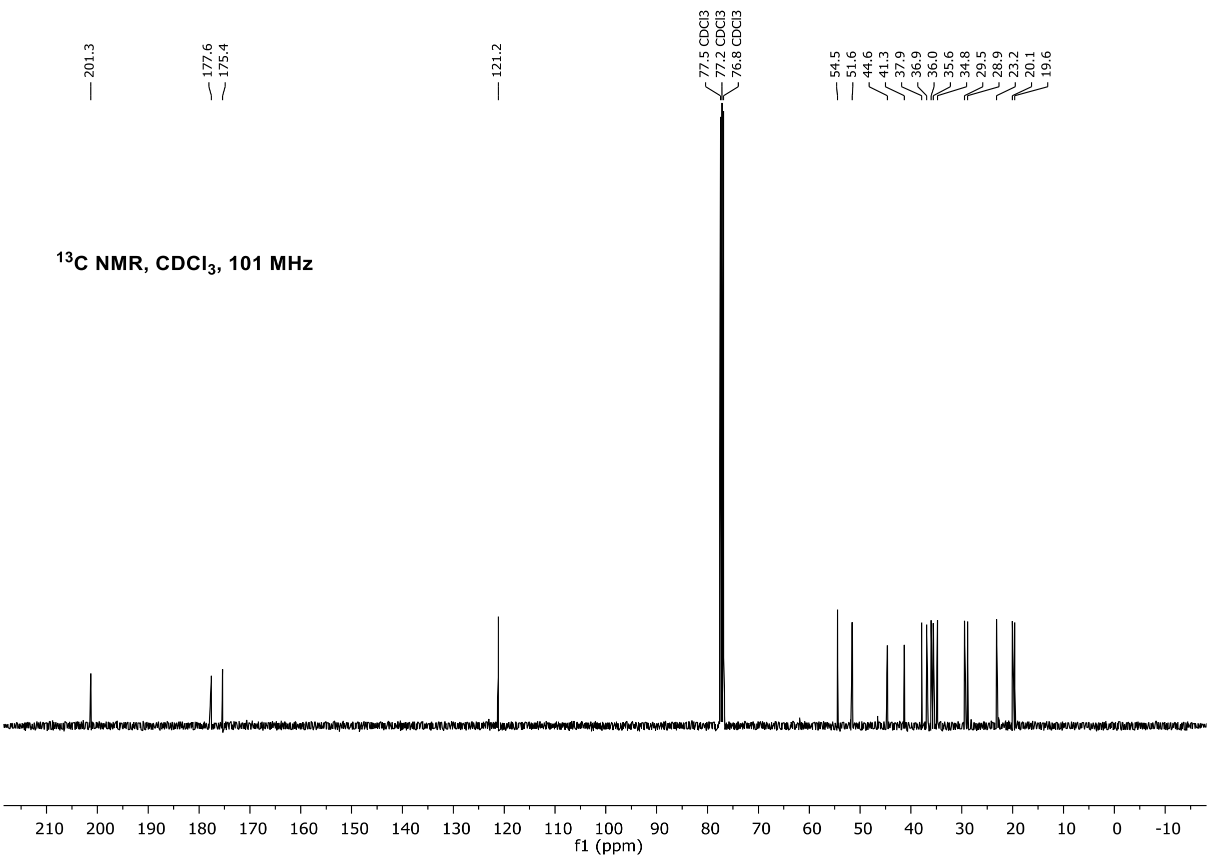

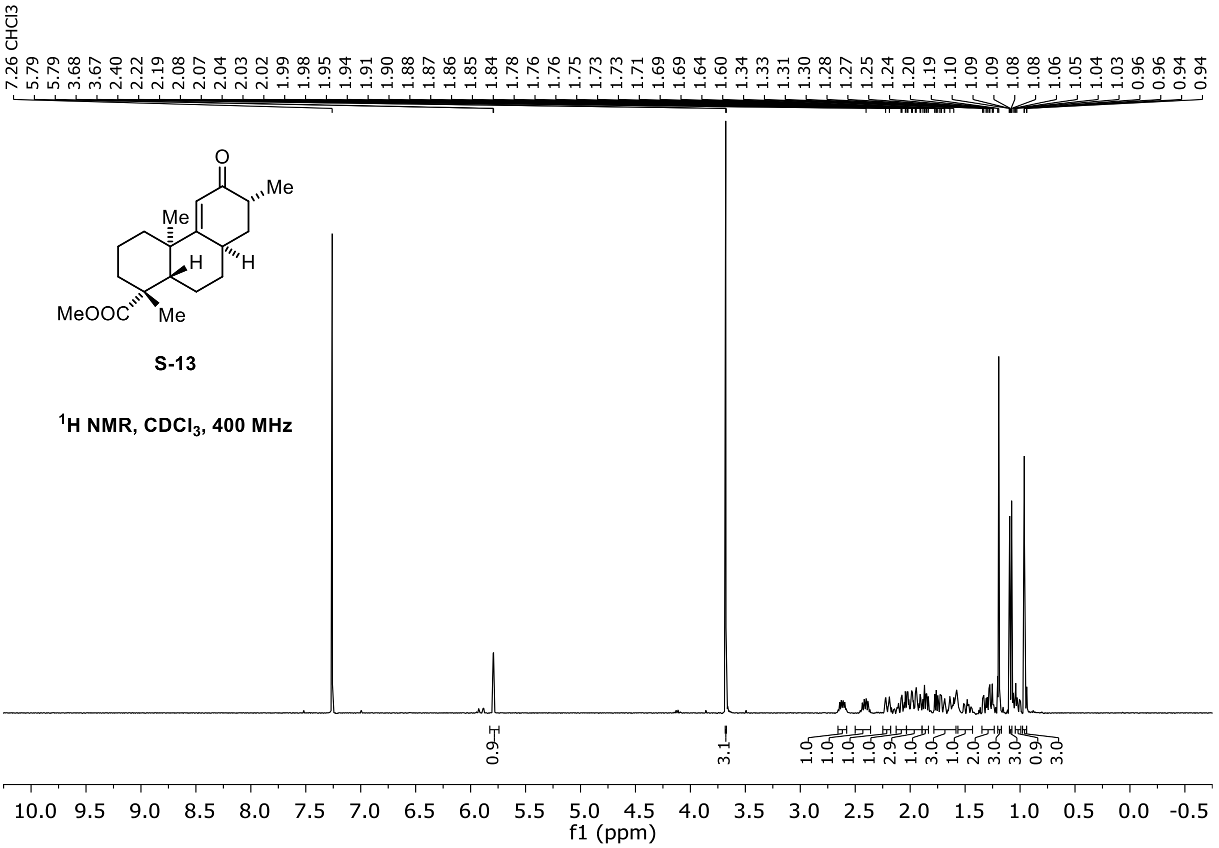
7.26 CHCl3
5.79
5.79
3.68
3.67
2.40
2.22
2.19
2.08
2.07
2.04
2.03
2.02
1.99
1.98
1.95
1.94
1.91
1.90
1.88
1.87
1.86
1.85
1.84
1.78
1.76
1.76
1.75
1.73
1.73
1.71
1.69
1.64
1.60
1.34
1.33
1.31
1.30
1.28
1.27
1.25
1.24
1.20
1.19
1.10
1.09
1.09
1.08
1.08
1.06
1.05
1.04
1.03
0.96
0.96
0.94
0.94
O
Me
Me
H
H
MeOOC
Me
S-13
^{1}H NMR, CDCl$_3$, 400 MHz
0.9
3.1
1.0
1.0
1.0
1.0
2.9
1.0
3.0
1.0
2.0
3.0
3.0
0.9
3.0
10.0 9.5 9.0 8.5 8.0 7.5 7.0 6.5 6.0 5.5 5.0 4.5 4.0 3.5 3.0 2.5 2.0 1.5 1.0 0.5 0.0 -0.5
f1 (ppm)

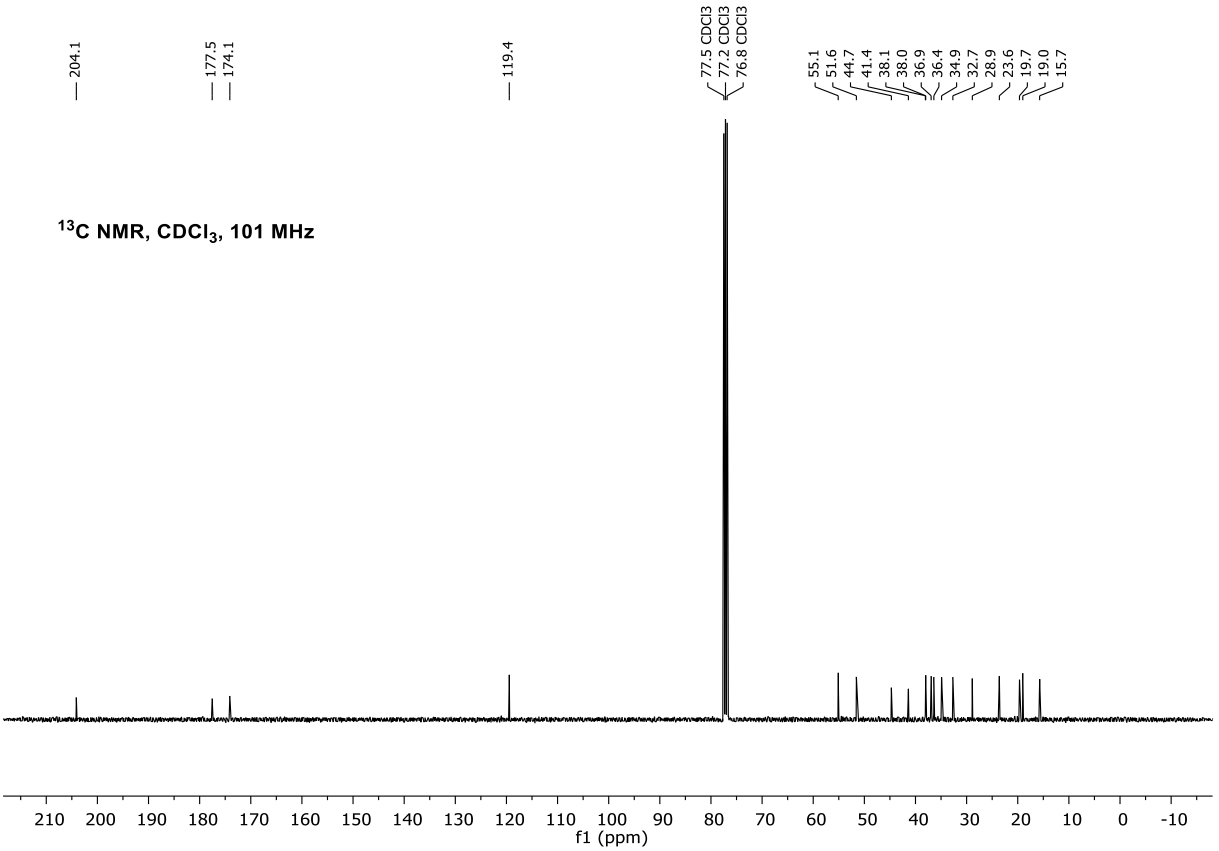
204.1
177.5
174.1
119.4
77.5 CDCl3
77.2 CDCl3
76.8 CDCl3
55.1
51.6
44.7
41.4
38.1
38.0
36.9
36.4
34.9
32.7
28.9
23.6
19.7
19.0
15.7
^{13}C NMR, CDCl$_3$, 101 MHz
210 200 190 180 170 160 150 140 130 120 110 100 90 80 70 60 50 40 30 20 10 0 -10
f1 (ppm)

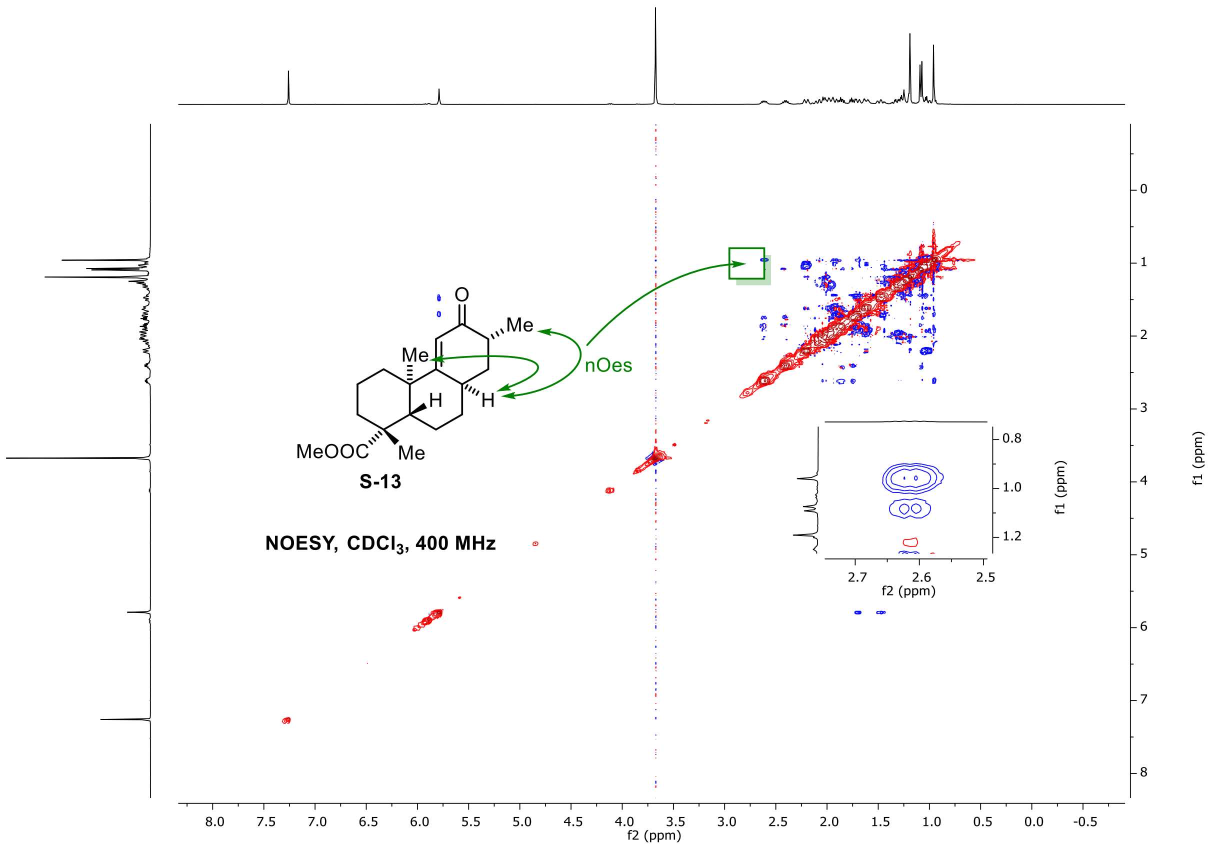

nOes
MeOOC
Me
S-13
NOESY, CDCl3, 400 MHz
f2 (ppm)
f1 (ppm)

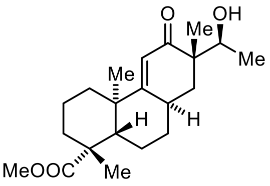
O
OH
Me
Me
Me
H
H
MeOOC
Me

S-14

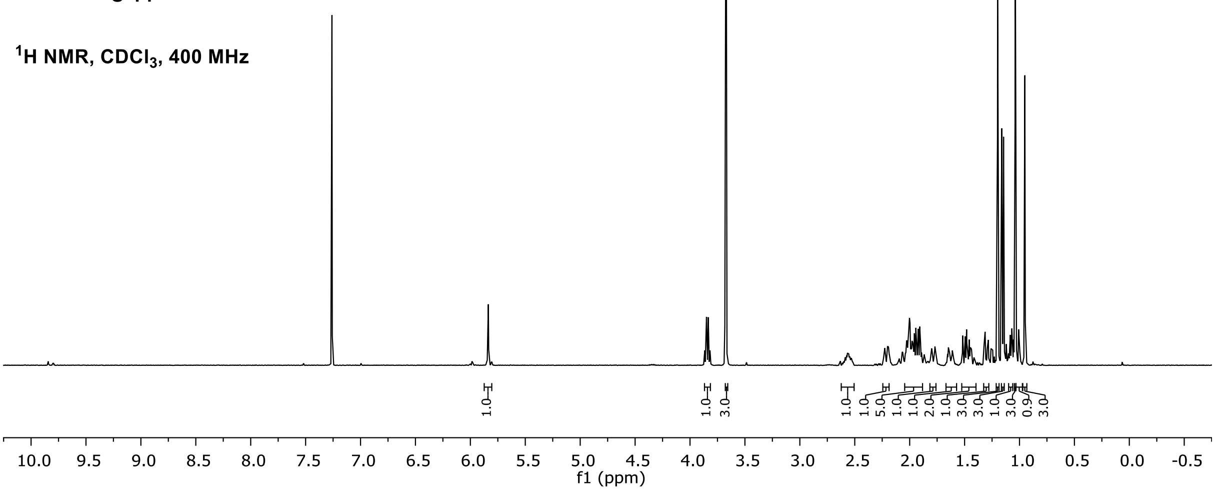
7.26 CHCl3
5.84
5.84
3.87
3.85
3.83
3.82
3.68
3.67
2.23
2.23
2.20
2.20
2.19
2.07
2.06
2.06
2.04
2.03
2.03
2.02
2.01
2.01
2.00
2.00
2.00
1.99
1.98
1.97
1.96
1.96
1.94
1.94
1.93
1.92
1.91
1.90
1.89
1.80
1.77
1.77
1.75
1.65
1.64
1.61
1.52
1.49
1.48
1.46
1.45
1.44
1.32
1.31
1.29
1.28
1.26
1.24
1.20
1.16
1.15
1.13
1.12
1.09
1.08
1.07
1.07
1.06
1.05
1.05
1.04
1.03
1.01
1.00
0.95
0.95
1H NMR, CDCl3, 400 MHz
1.0
1.0
3.0
1.0
1.0
5.0
1.0
1.0
2.0
1.0
3.0
3.0
1.0
3.0
0.9
3.0
10.0 9.5 9.0 8.5 8.0 7.5 7.0 6.5 6.0 5.5 5.0 4.5 4.0 3.5 3.0 2.5 2.0 1.5 1.0 0.5 0.0 -0.5
f1 (ppm)

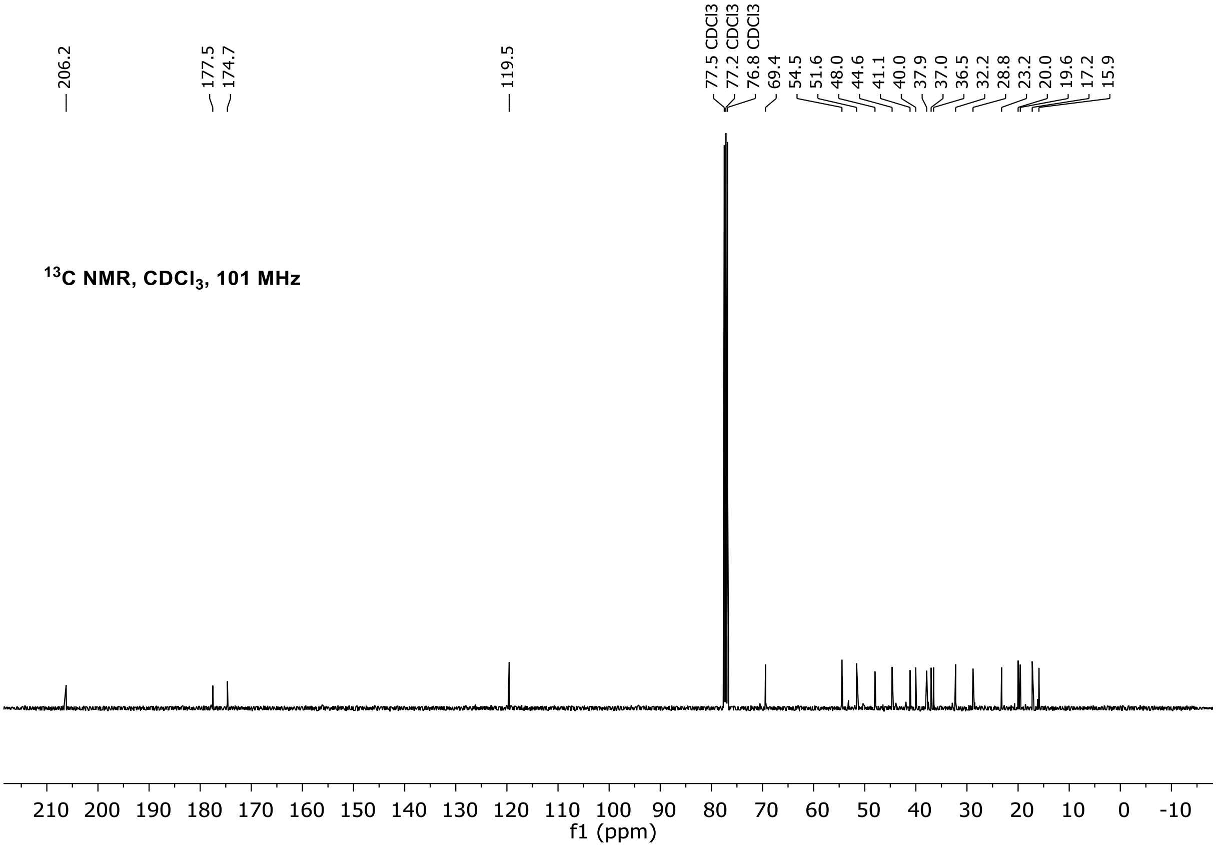
206.2
177.5
174.7
119.5
77.5 CDCl3
77.2 CDCl3
76.8 CDCl3
69.4
54.5
51.6
48.0
44.6
41.1
40.0
37.9
37.0
36.5
32.2
28.8
23.2
20.0
19.6
17.2
15.9
13C NMR, CDCl3, 101 MHz
210 200 190 180 170 160 150 140 130 120 110 100 90 80 70 60 50 40 30 20 10 0 -10
f1 (ppm)

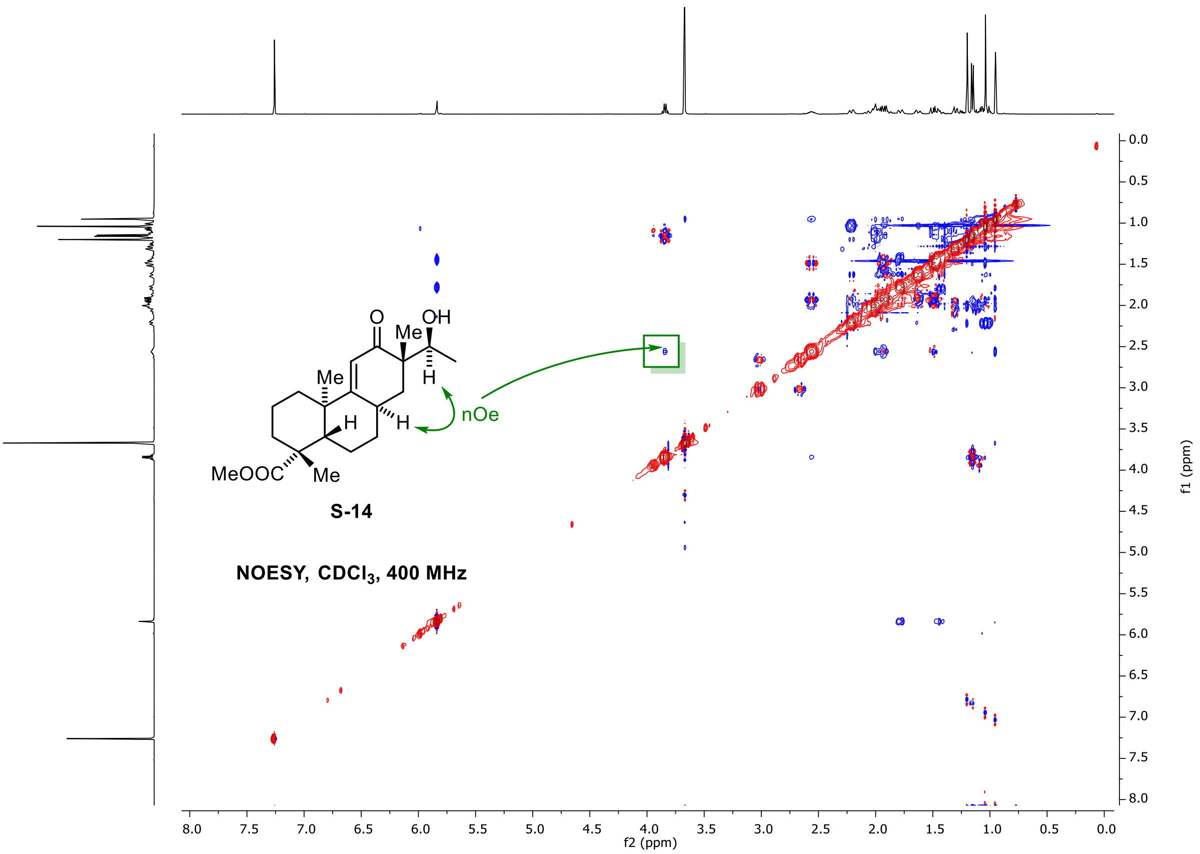

O
OH
Me
H
Me
H
H
nOe
MeOOC
Me
S-14
NOESY, CDCl3, 400 MHz
f2 (ppm)
f1 (ppm)

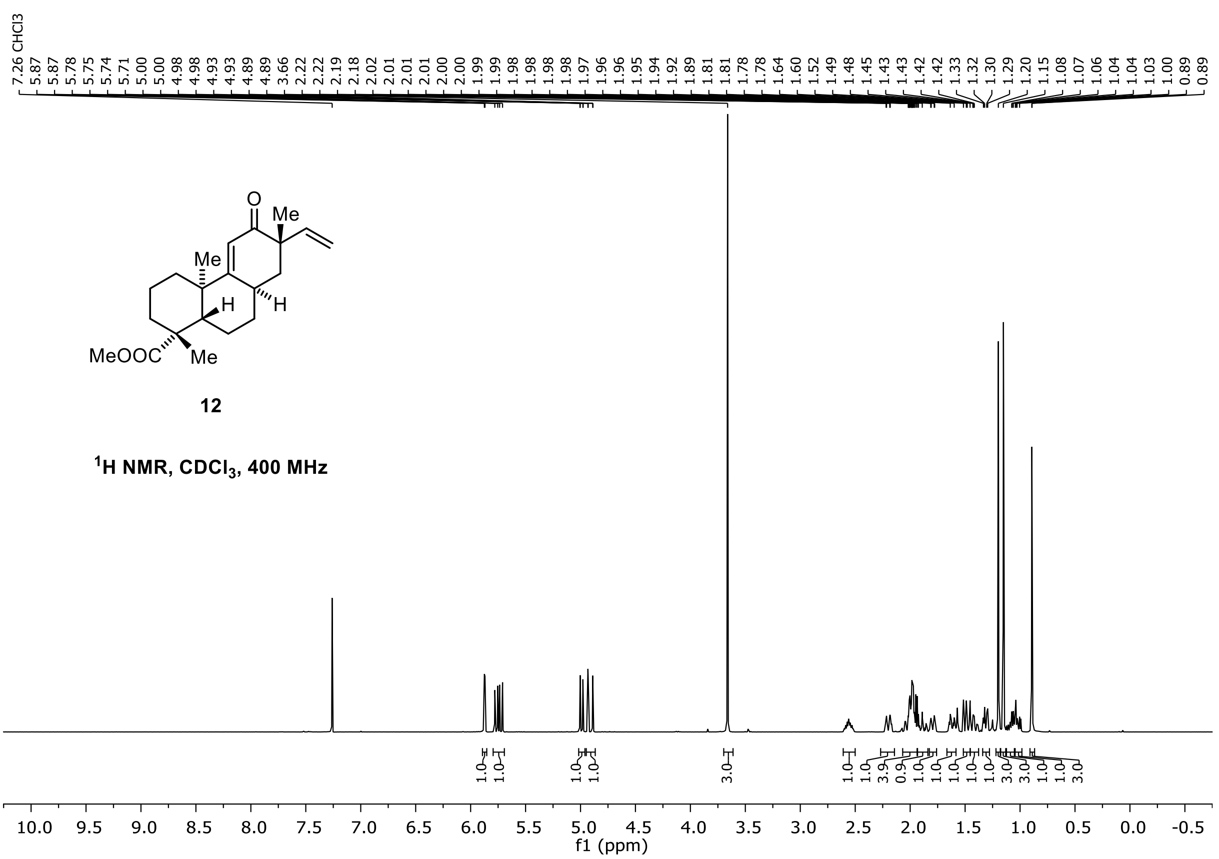

12

^{1}H NMR, $CDCl_3$, 400 MHz

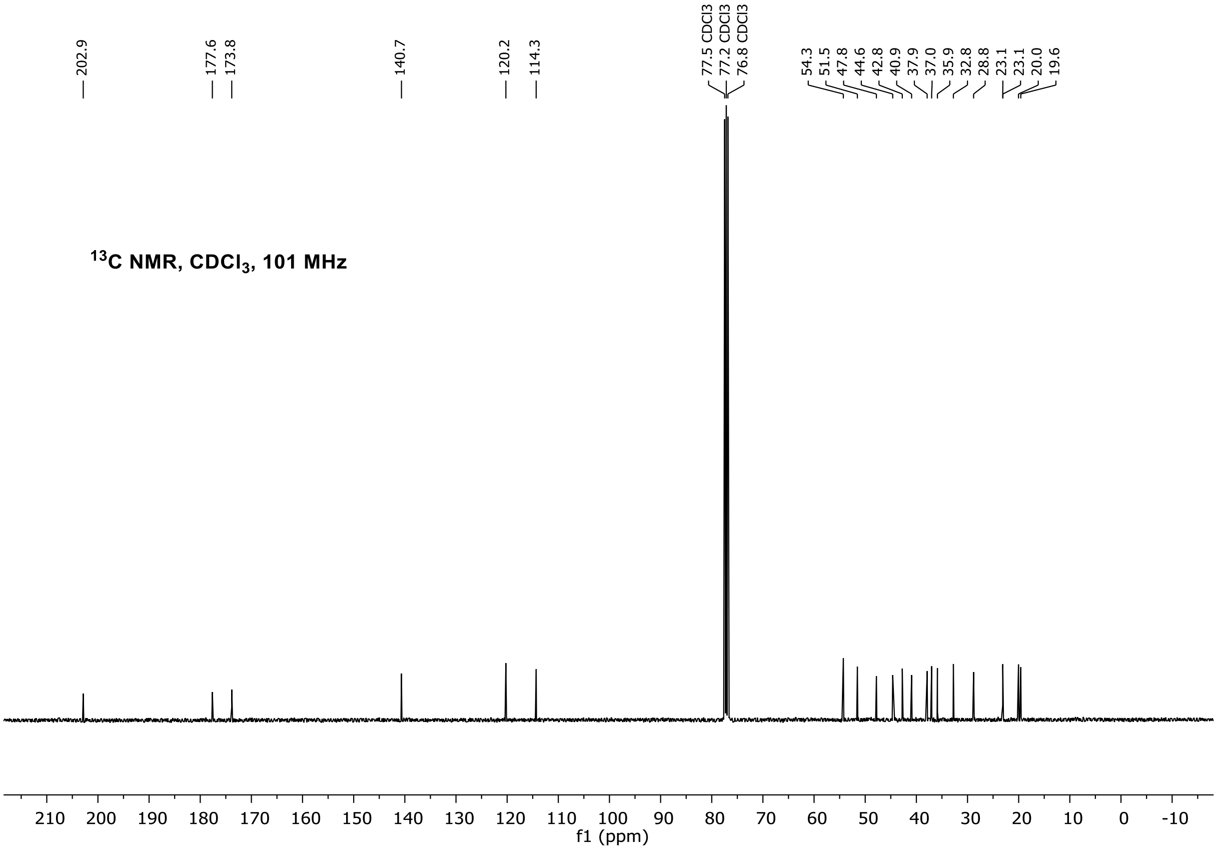

^{13}C NMR, $CDCl_3$, 101 MHz

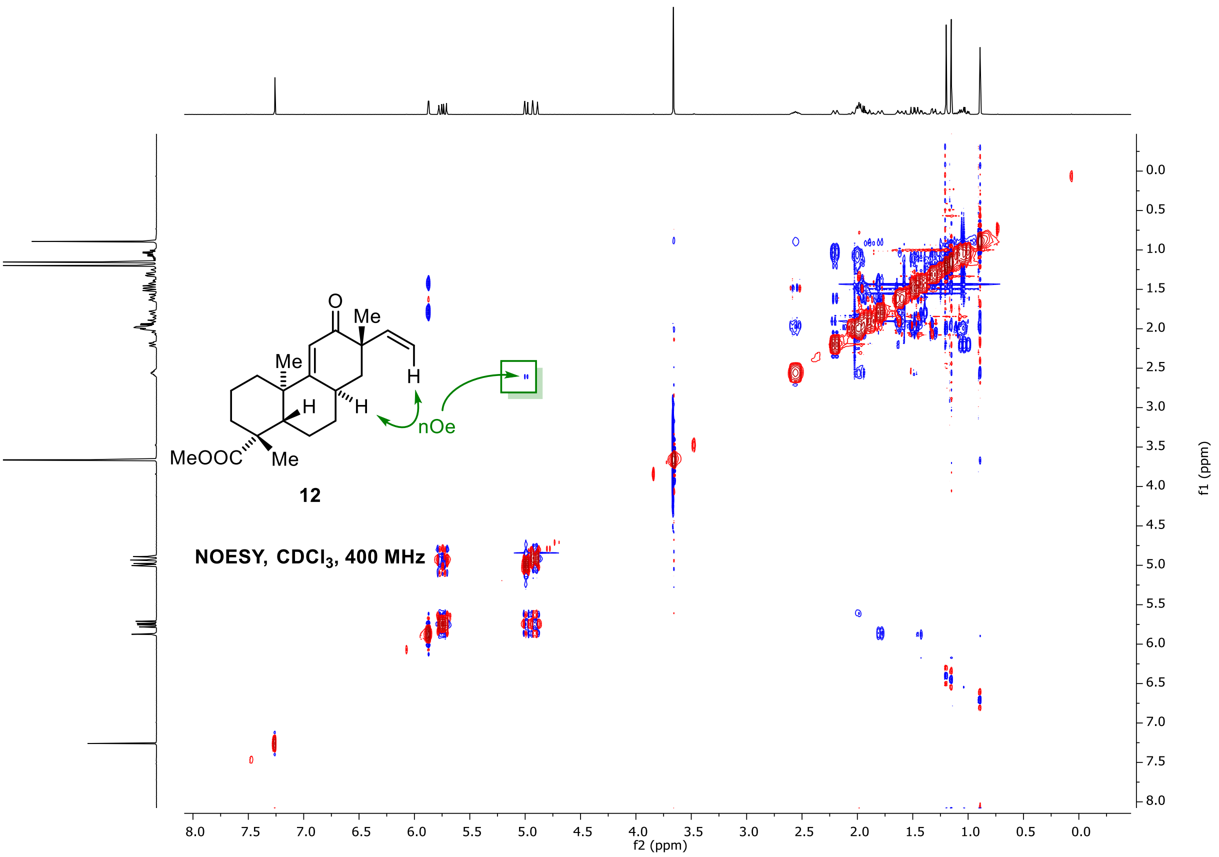
O
Me
Me
H
H
H
nOe
MeOOC
Me
12
NOESY, CDCl3, 400 MHz
f2 (ppm)
f1 (ppm)

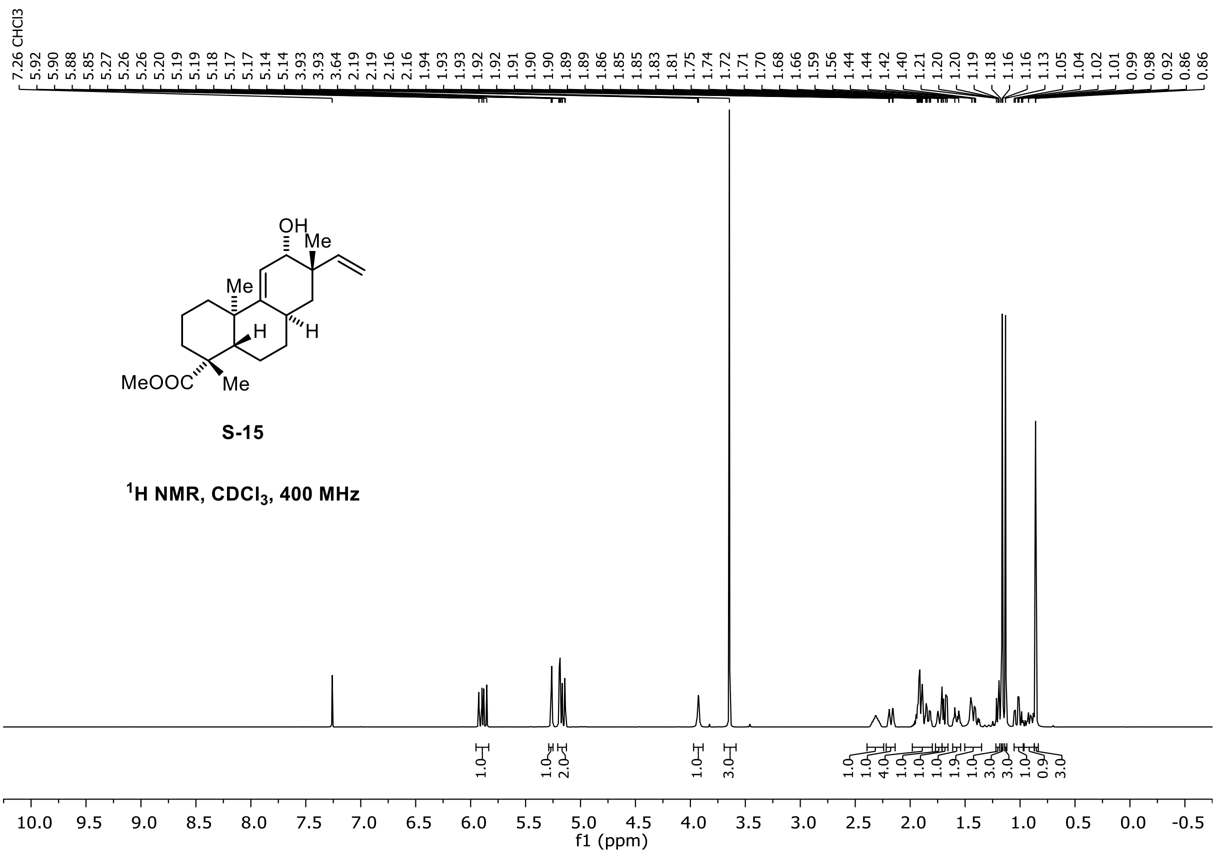

S-15

^{1}H NMR, $CDCl_3$, 400 MHz

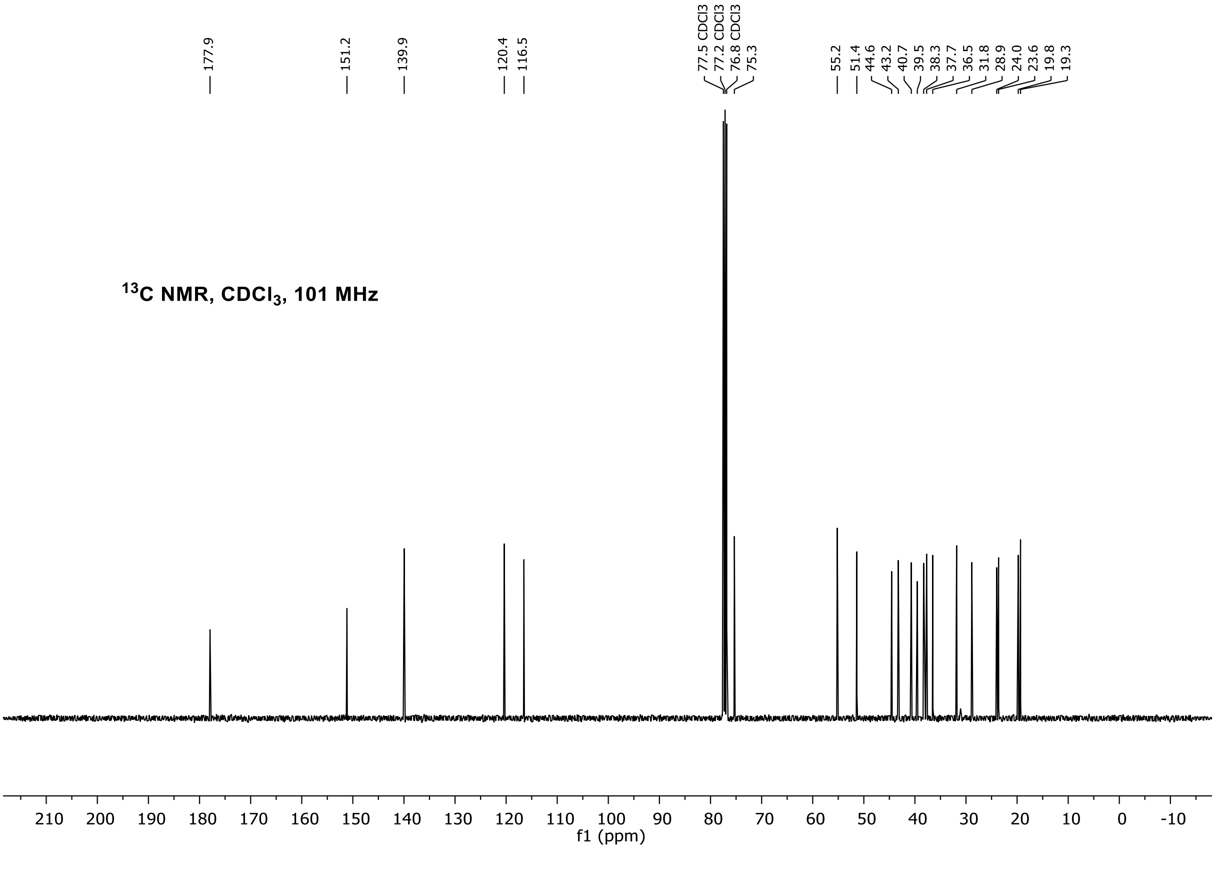

^{13}C NMR, $CDCl_3$, 101 MHz

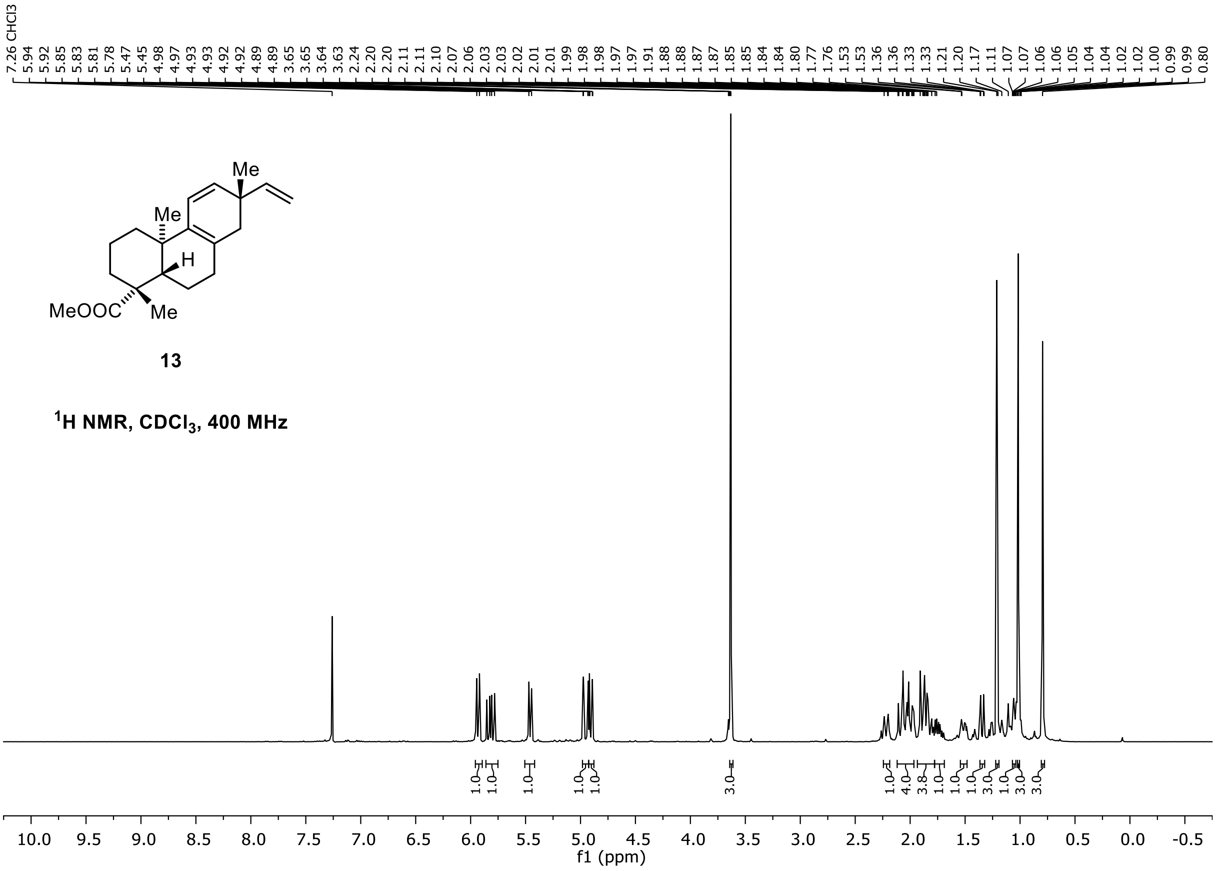

7.26 CHCl3
5.94 5.92 5.85 5.83 5.81 5.78 5.47 5.45 4.98 4.97 4.93 4.93 4.92 4.92 4.89 3.65 3.65 3.64 3.63 2.24 2.20 2.20 2.11 2.11 2.10 2.07 2.06 2.03 2.03 2.02 2.01 2.01 1.99 1.98 1.98 1.97 1.97 1.91 1.88 1.87 1.87 1.85 1.85 1.84 1.84 1.80 1.77 1.76 1.53 1.53 1.36 1.36 1.33 1.33 1.21 1.20 1.17 1.11 1.07 1.07 1.06 1.06 1.05 1.04 1.04 1.02 1.02 1.00 0.99 0.99 0.80
Me
Me
H
MeOOC
Me
13
¹H NMR, CDCl₃, 400 MHz
1.0 1.0 1.0 1.0 1.0 1.0 3.0 1.0 4.0 3.8 1.0 1.0 1.0 3.0 1.0 3.0 3.0
10.0 9.5 9.0 8.5 8.0 7.5 7.0 6.5 6.0 5.5 5.0 4.5 4.0 3.5 3.0 2.5 2.0 1.5 1.0 0.5 0.0 -0.5
f1 (ppm)

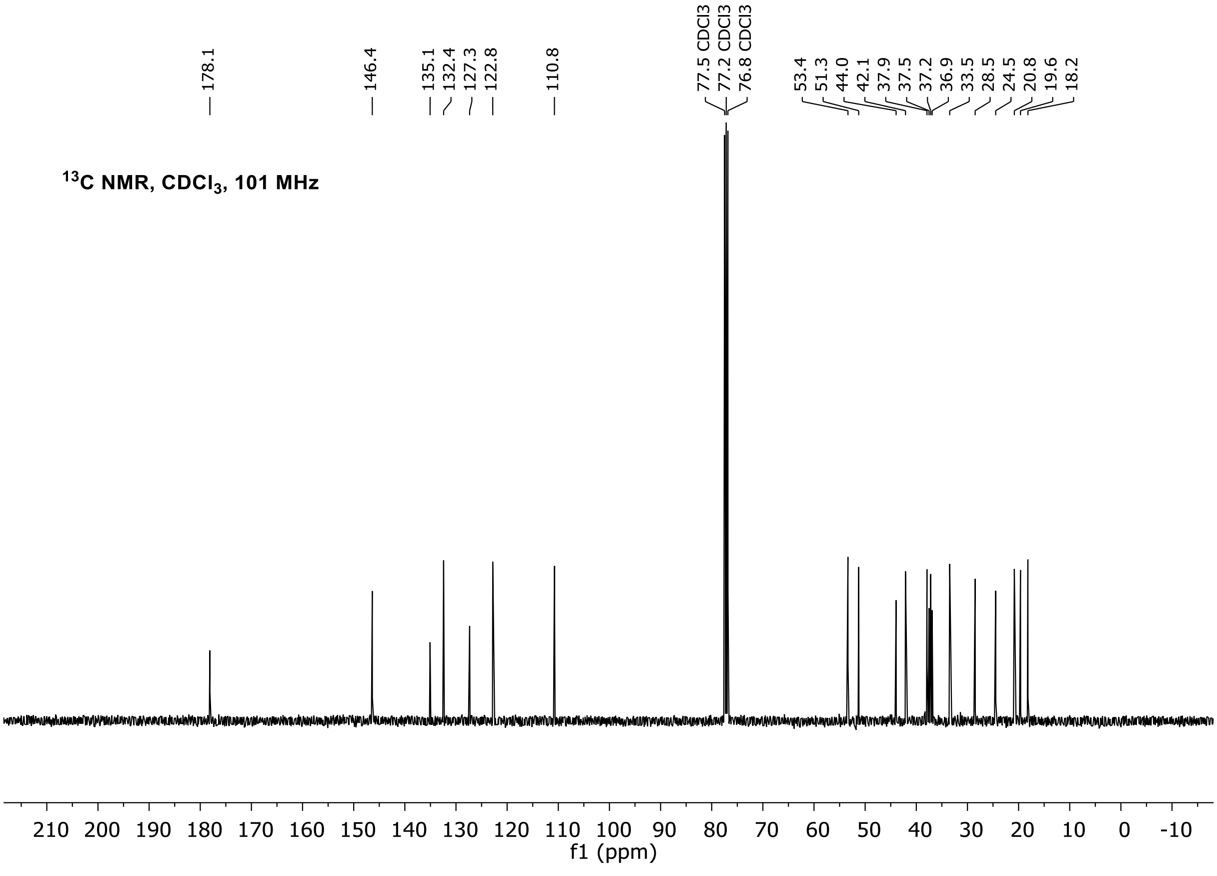

178.1
146.4
135.1 132.4 127.3 122.8
110.8
77.5 CDCl3 77.2 CDCl3 76.8 CDCl3
53.4 51.3 44.0 42.1 37.9 37.5 37.2 36.9 33.5 28.5 24.5 20.8 19.6 18.2
¹³C NMR, CDCl₃, 101 MHz
210 200 190 180 170 160 150 140 130 120 110 100 90 80 70 60 50 40 30 20 10 0 -10
f1 (ppm)

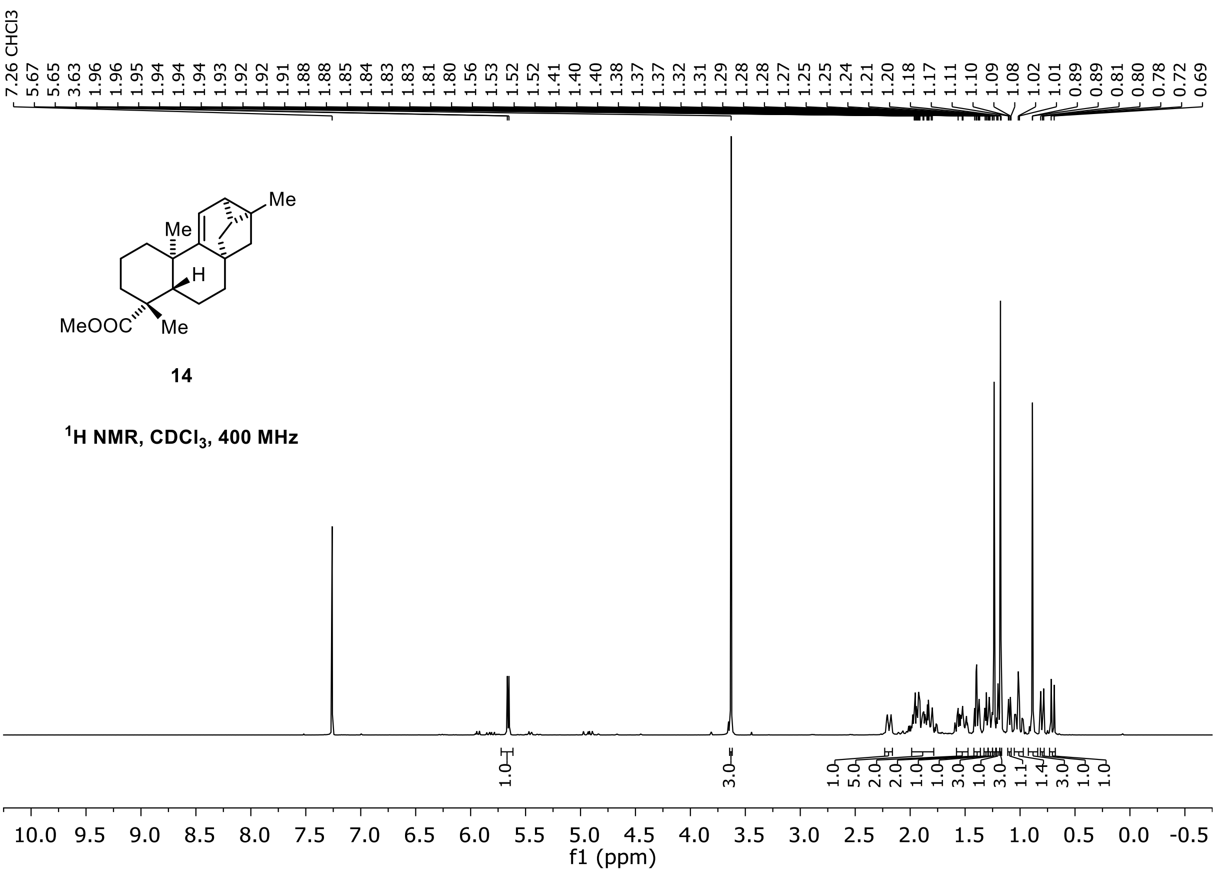

14

^{1}H NMR, $CDCl_3$, 400 MHz

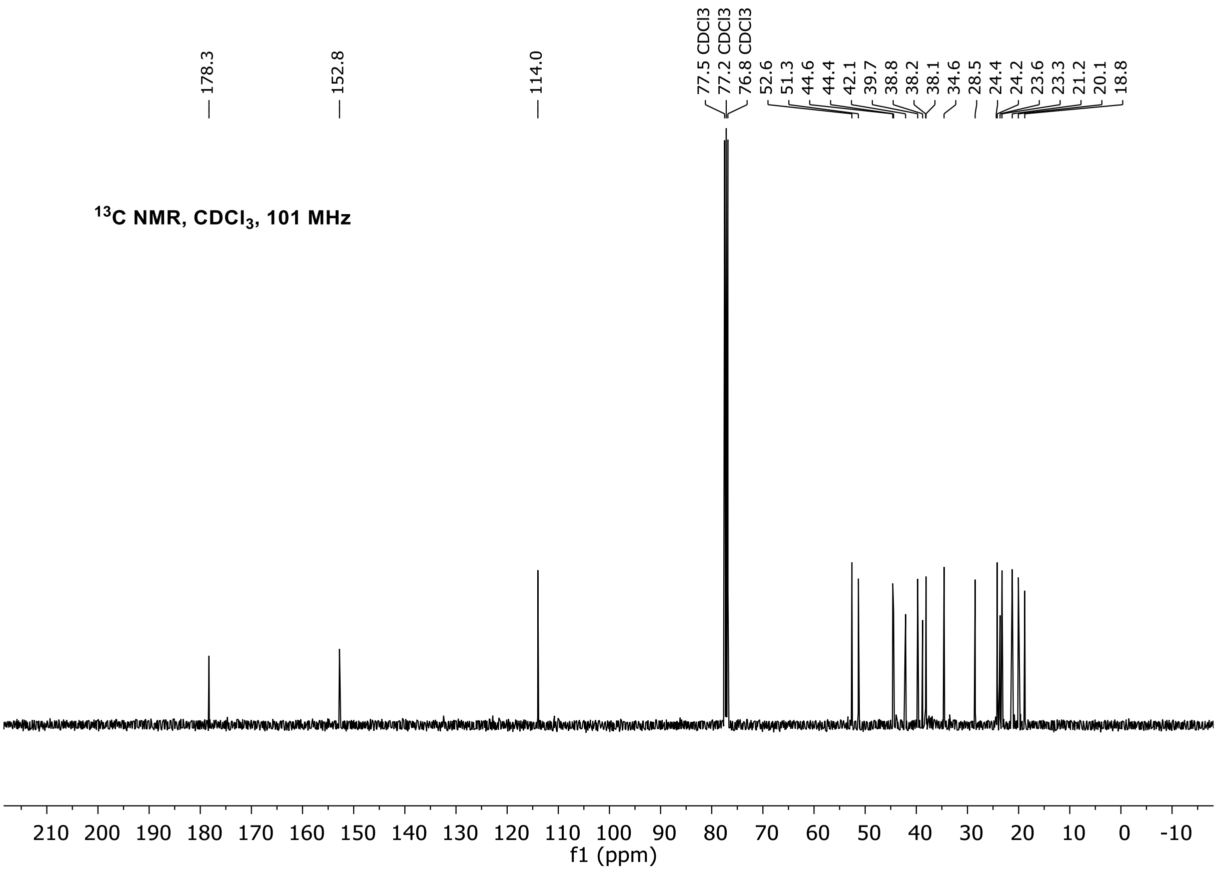

^{13}C NMR, $CDCl_3$, 101 MHz

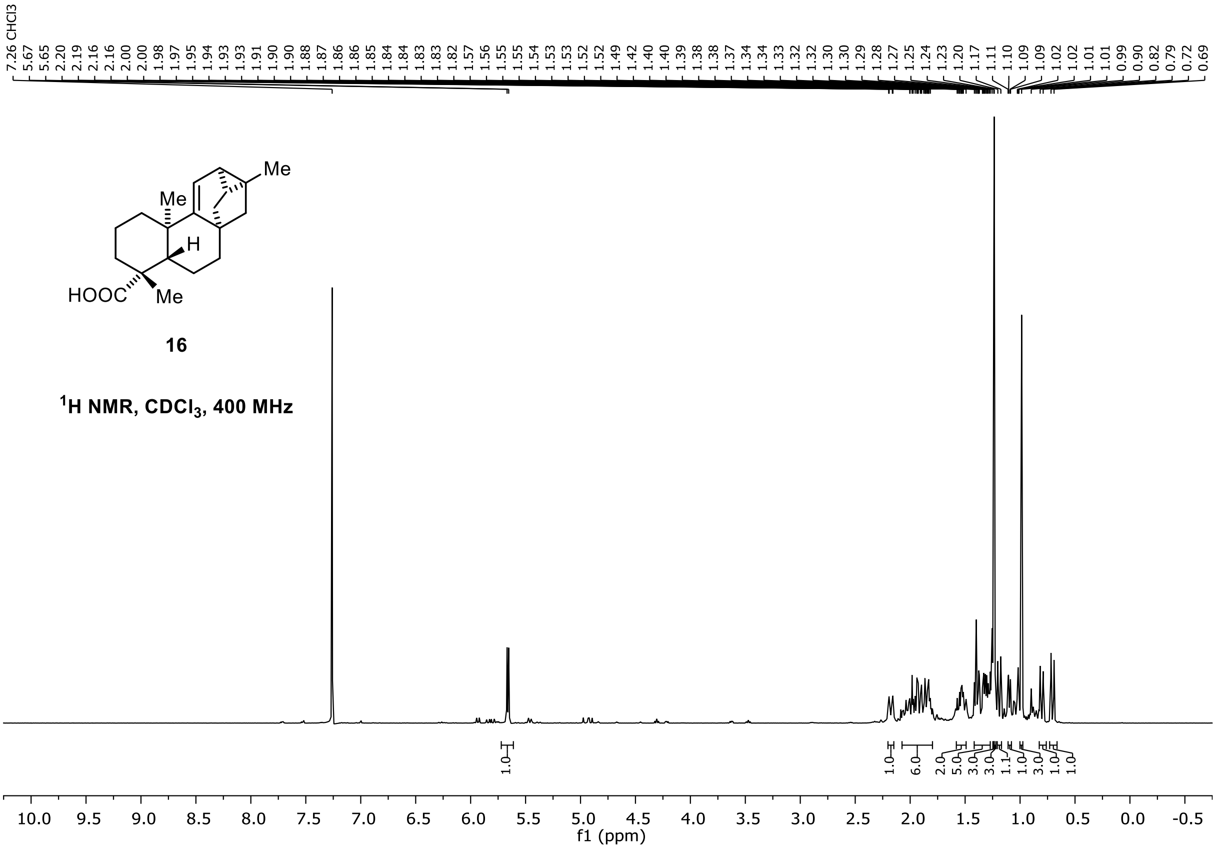
16
^{1}H NMR, CDCl$_3$, 400 MHz
f1 (ppm)

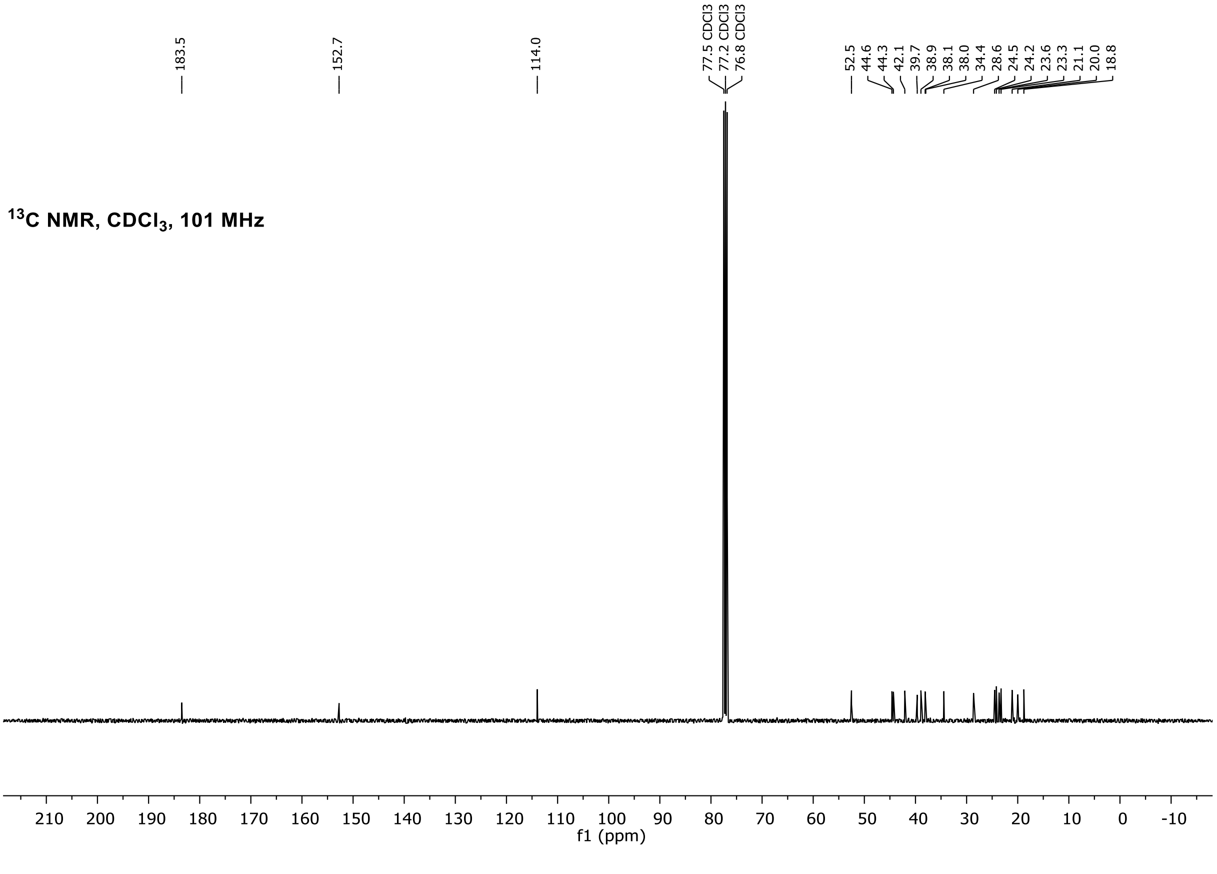
^{13}C NMR, CDCl$_3$, 101 MHz
183.5
152.7
114.0
77.5 CDCl3
77.2 CDCl3
76.8 CDCl3
52.5
44.6
44.3
42.1
39.7
38.9
38.1
38.0
34.4
28.6
24.5
24.2
23.6
23.3
21.1
20.0
18.8
f1 (ppm)

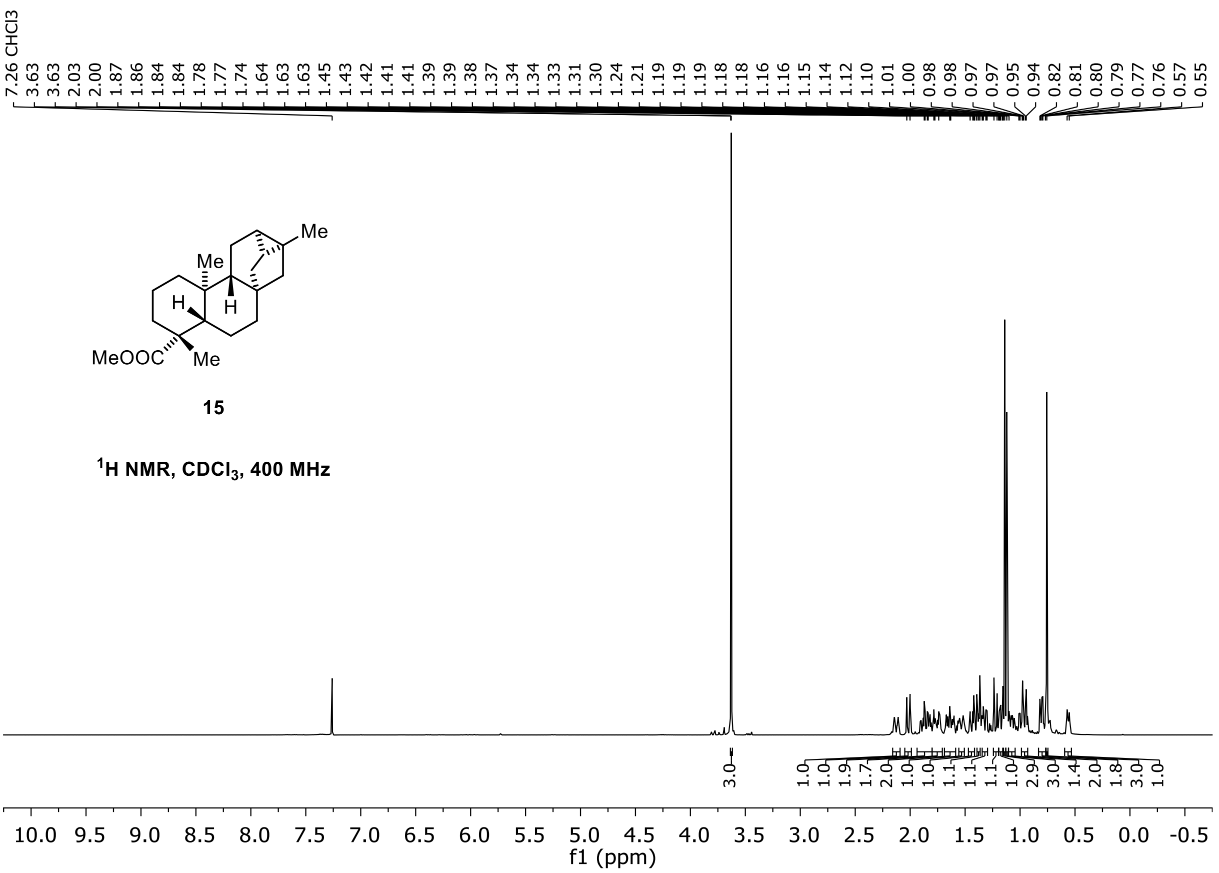
7.26 CHCl3
3.63 3.63 2.03 2.00 1.87 1.86 1.84 1.84 1.78 1.77 1.74 1.64 1.63 1.63 1.45 1.43 1.42 1.41 1.39 1.39 1.38 1.37 1.34 1.34 1.33 1.31 1.30 1.24 1.21 1.19 1.19 1.19 1.18 1.18 1.18 1.16 1.16 1.15 1.14 1.12 1.10 1.01 1.00 0.98 0.98 0.97 0.97 0.95 0.94 0.82 0.81 0.80 0.79 0.77 0.76 0.57 0.55
Me
Me
H
H
MeOOC
Me
15
^{1}H NMR, CDCl$_3$, 400 MHz
3.0
1.0 1.0 1.9 1.7 2.0 1.0 1.0 1.1 1.1 1.1 1.0 2.9 3.0 1.4 2.0 1.8 3.0 1.0
10.0 9.5 9.0 8.5 8.0 7.5 7.0 6.5 6.0 5.5 5.0 4.5 4.0 3.5 3.0 2.5 2.0 1.5 1.0 0.5 0.0 -0.5
f1 (ppm)

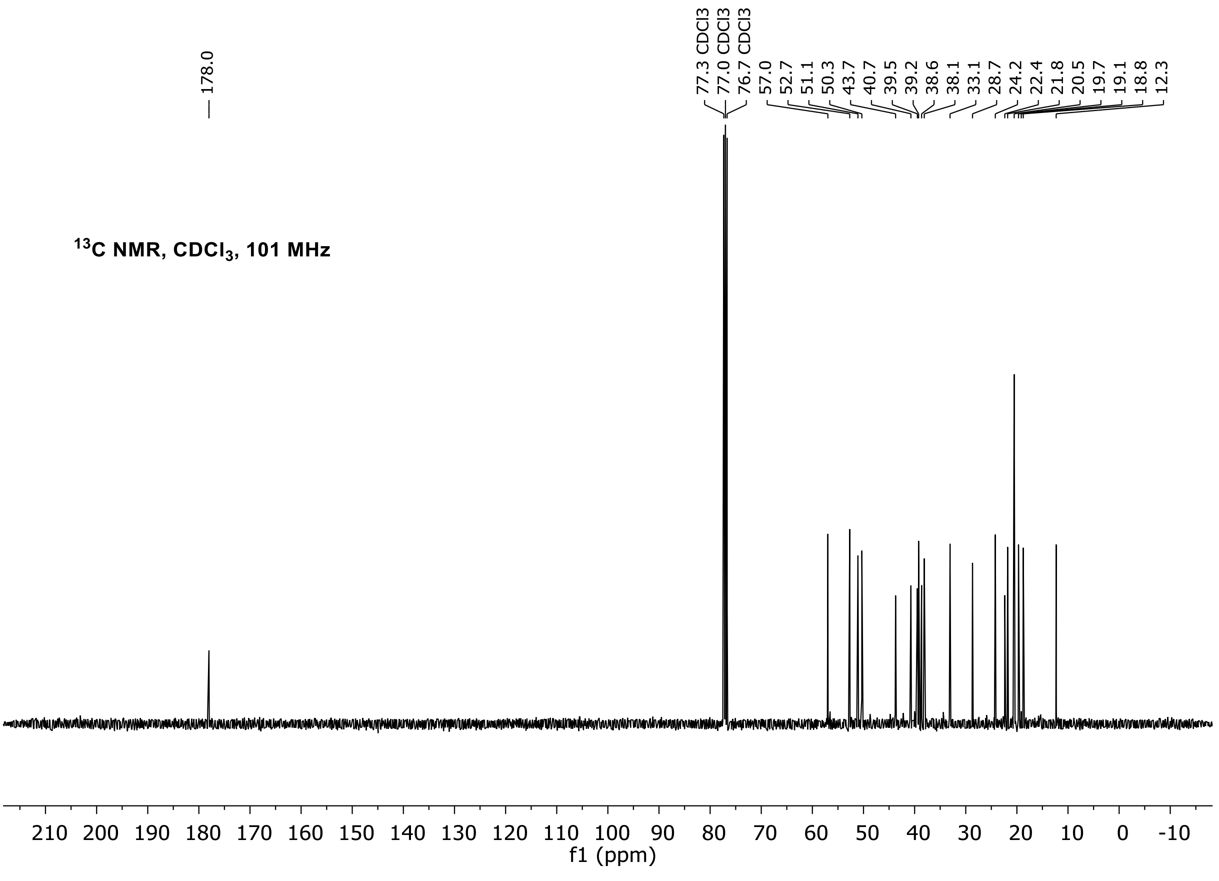
178.0
77.3 CDCl3
77.0 CDCl3
76.7 CDCl3
57.0 52.7 51.1 50.3 43.7 40.7 39.5 39.2 38.6 38.1 33.1 28.7 24.2 22.4 21.8 20.5 19.7 19.1 18.8 12.3
^{13}C NMR, CDCl$_3$, 101 MHz
210 200 190 180 170 160 150 140 130 120 110 100 90 80 70 60 50 40 30 20 10 0 -10
f1 (ppm)

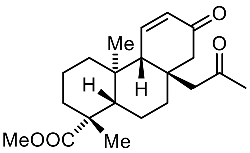

20

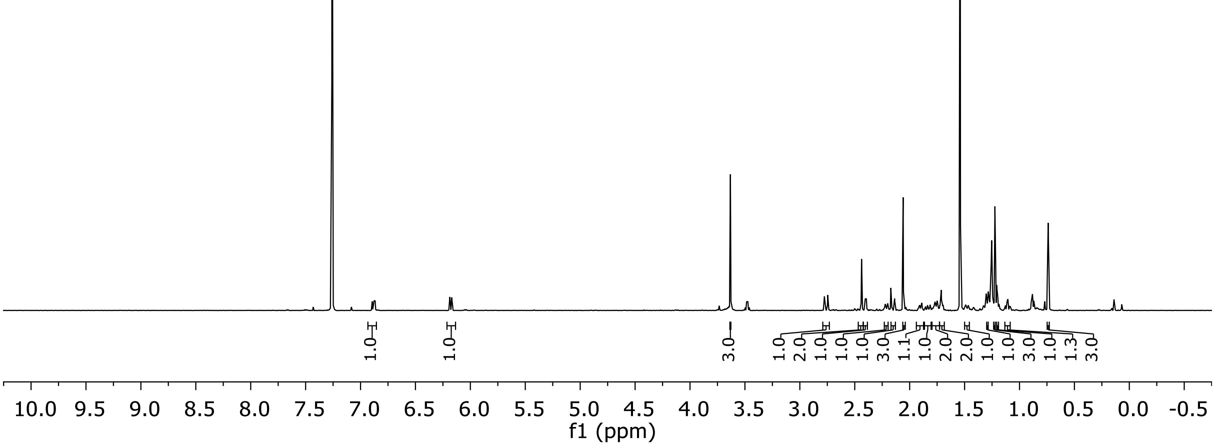

^{1}H NMR, $CDCl_3$, 600 MHz

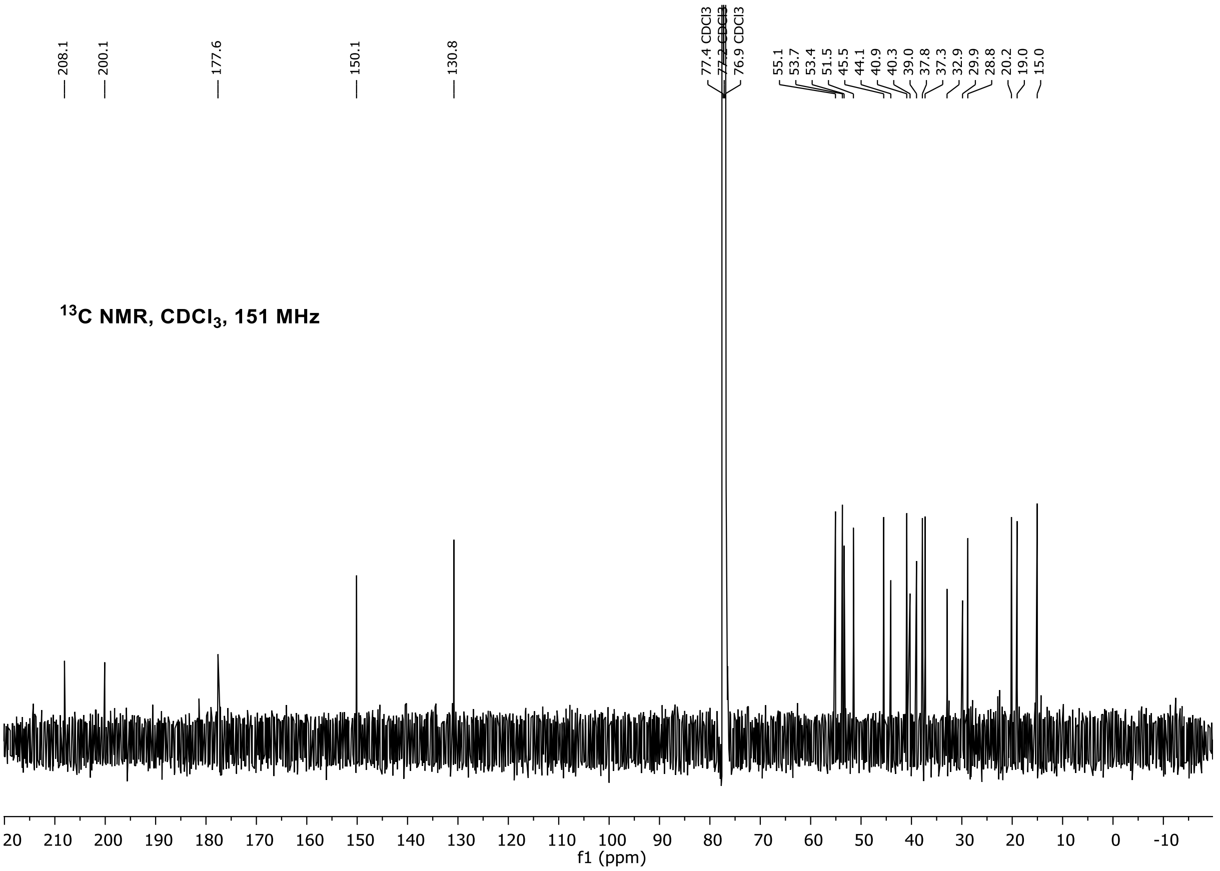

^{13}C NMR, $CDCl_3$, 151 MHz

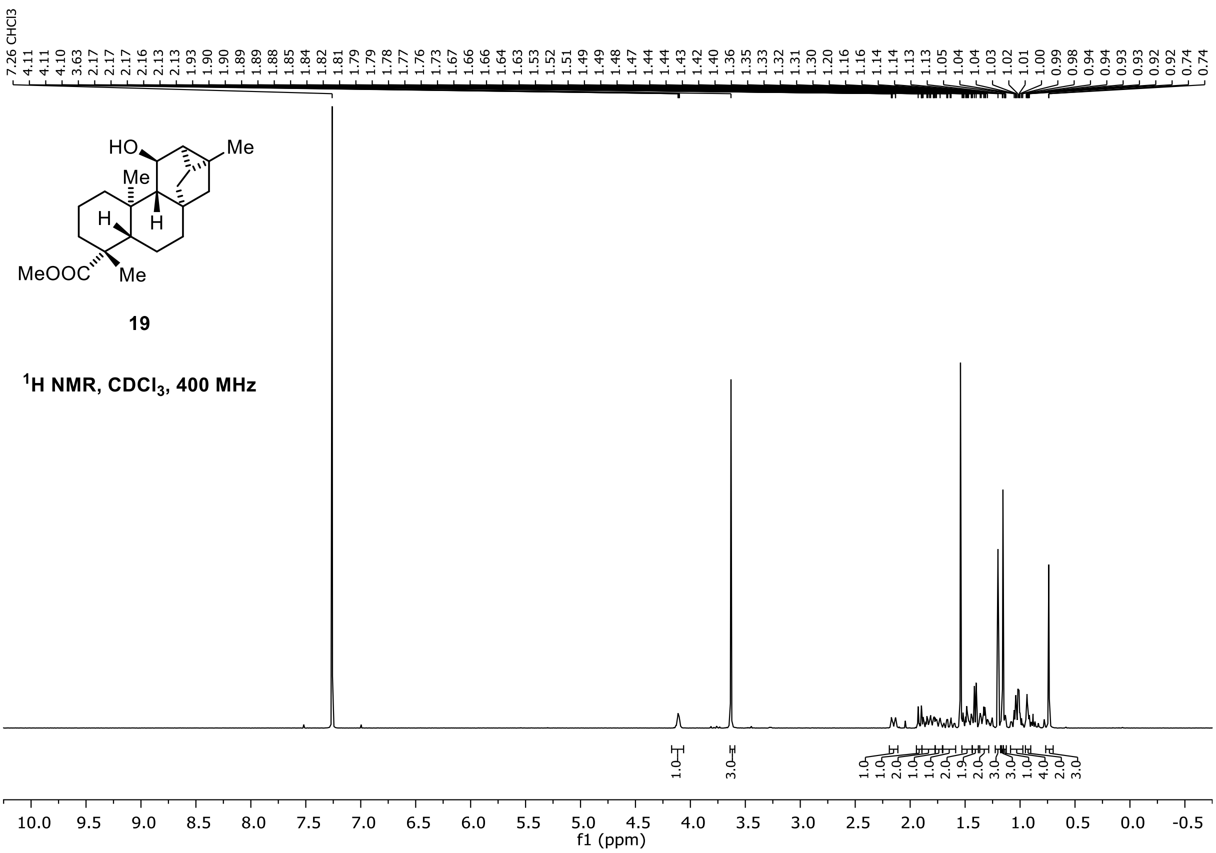

19

^{1}H NMR, CDCl$_3$, 400 MHz

^{13}C NMR, CDCl$_3$, 101 MHz

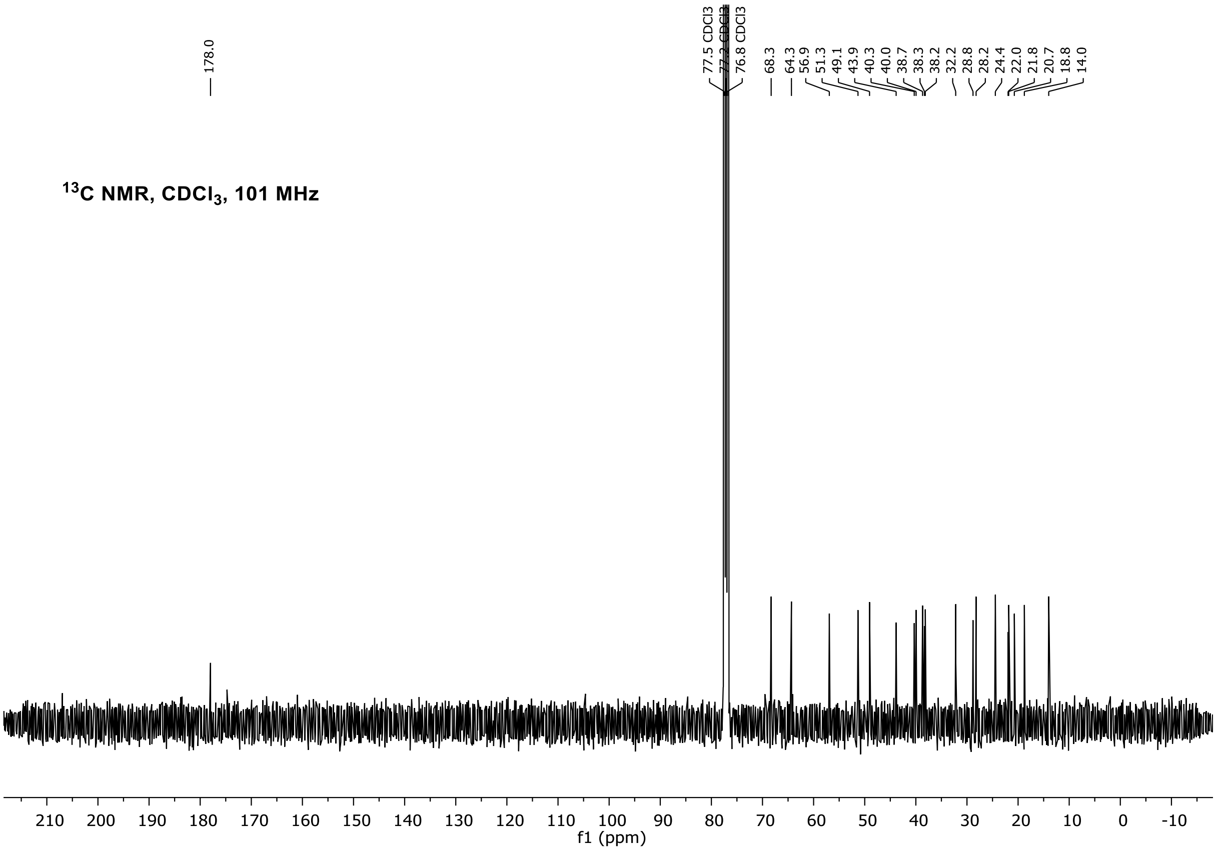

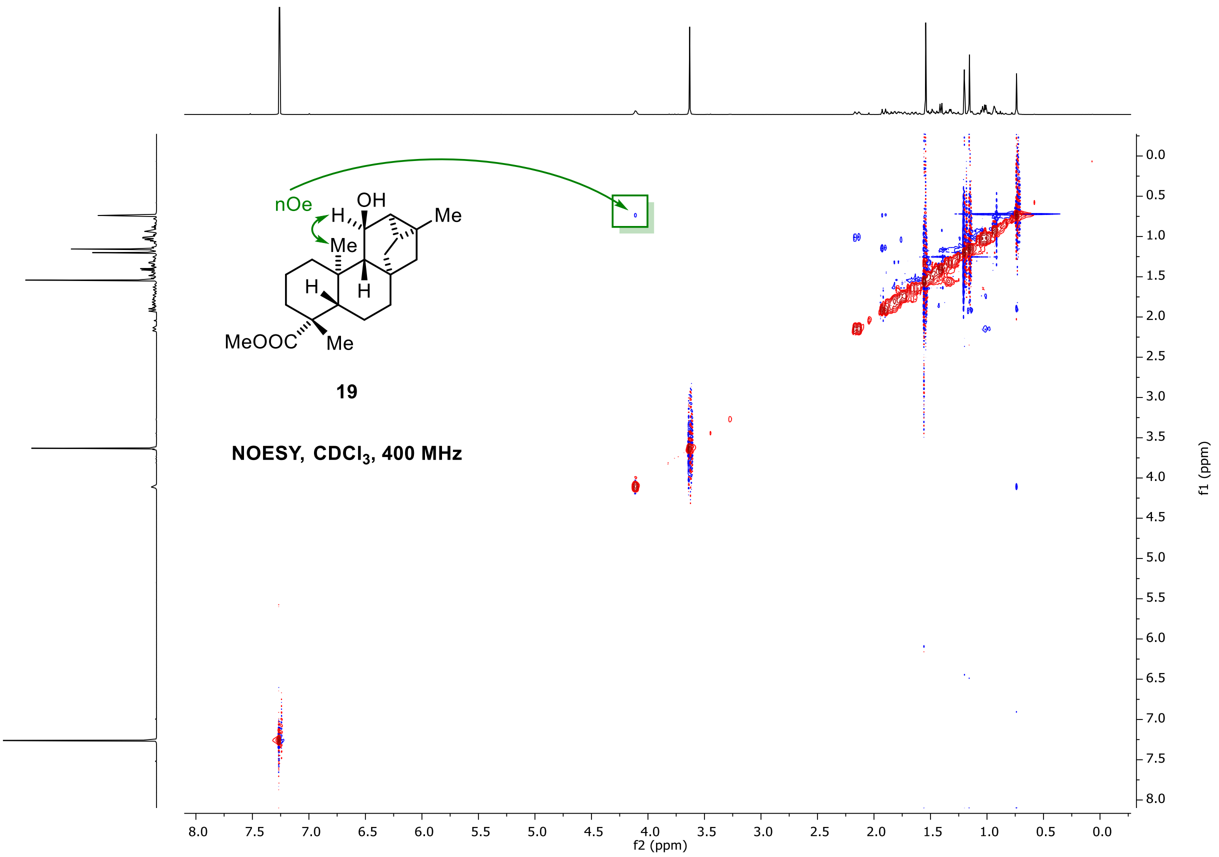
nOe
OH
H
Me
Me
H
H
MeOOC
Me
19
NOESY, CDCl3, 400 MHz
f1 (ppm)
f2 (ppm)

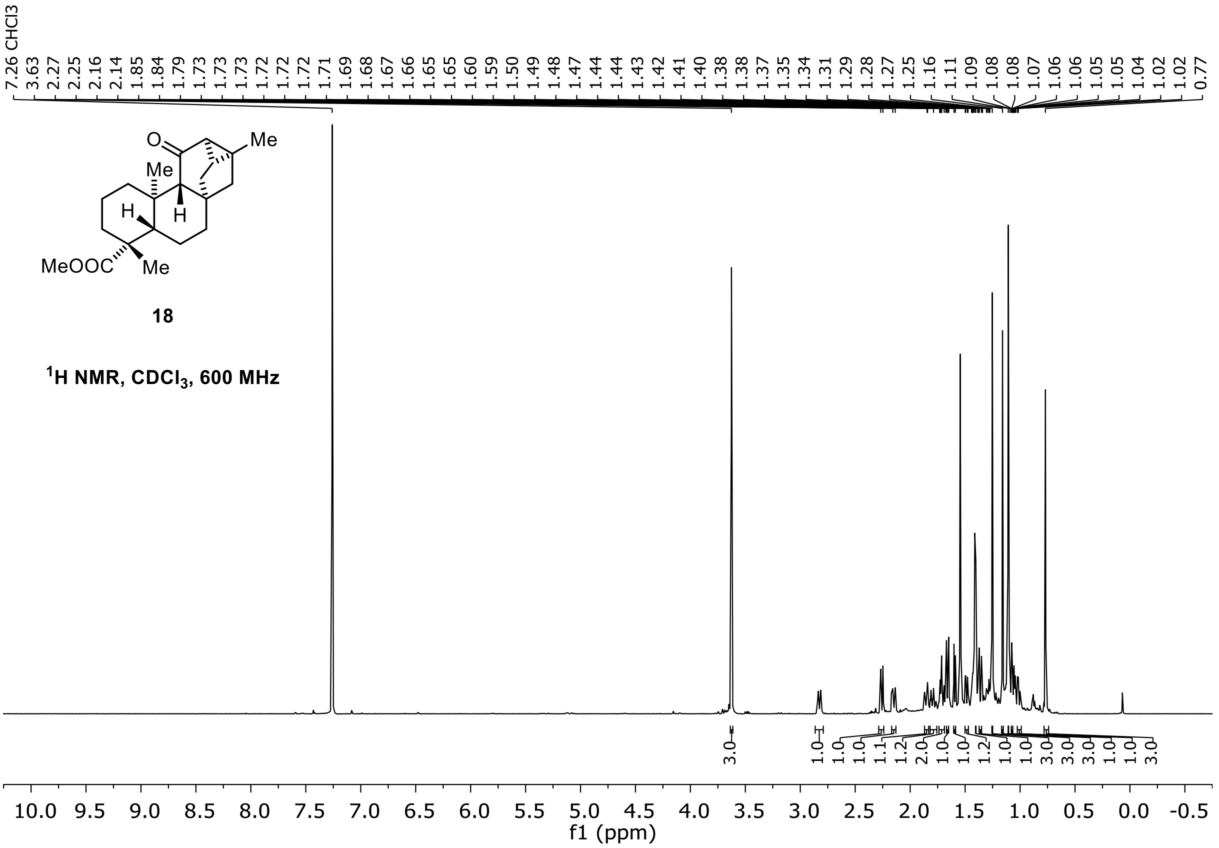

18

^{1}H NMR, $CDCl_3$, 600 MHz

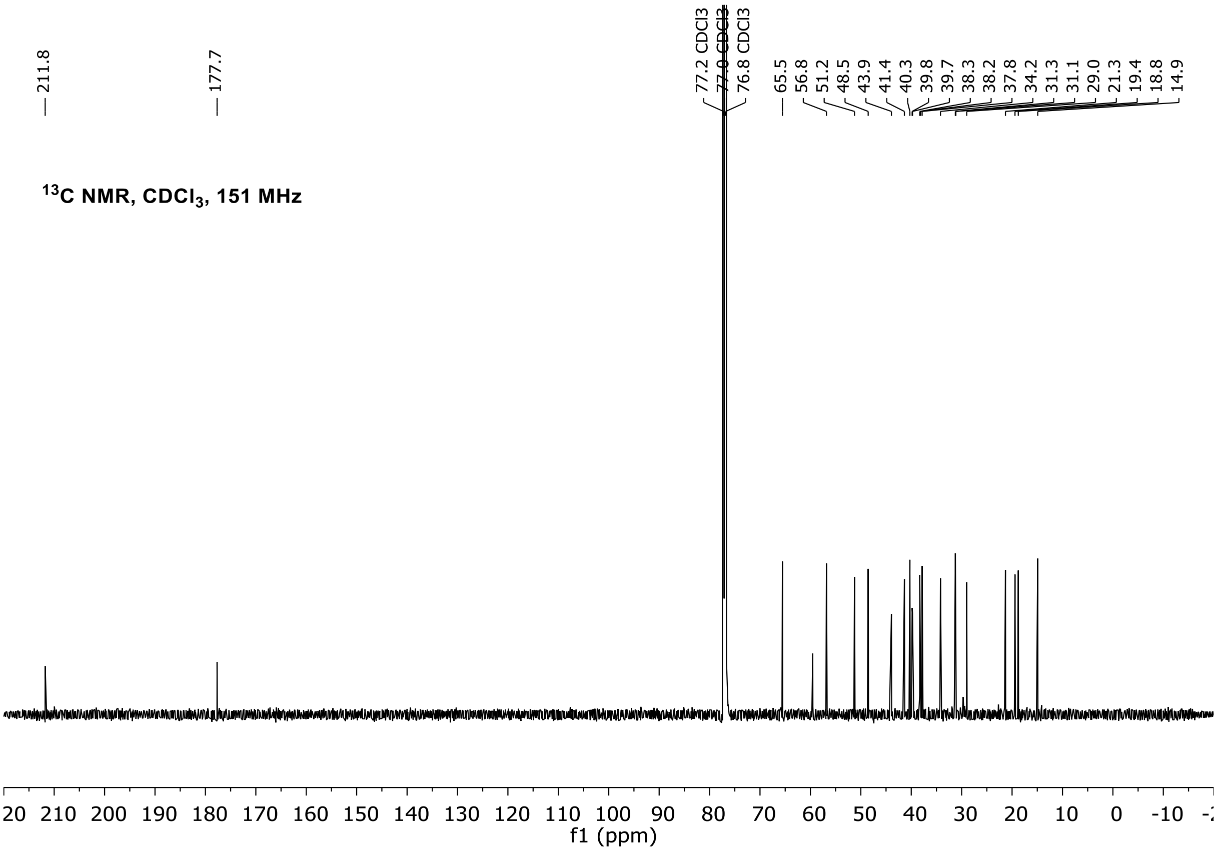

^{13}C NMR, $CDCl_3$, 151 MHz

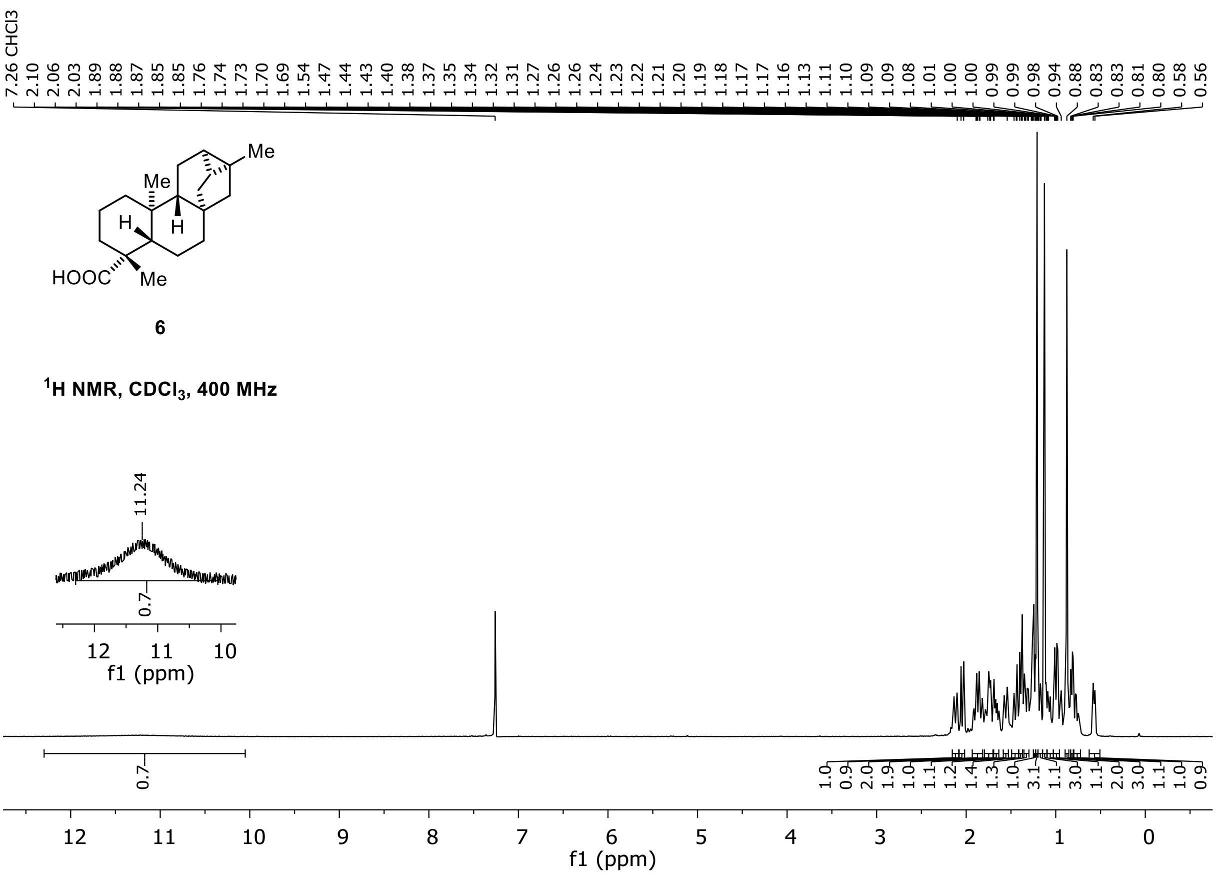

6

^{1}H NMR, CDCl$_3$, 400 MHz

^{13}C NMR, CDCl$_3$, 101 MHz

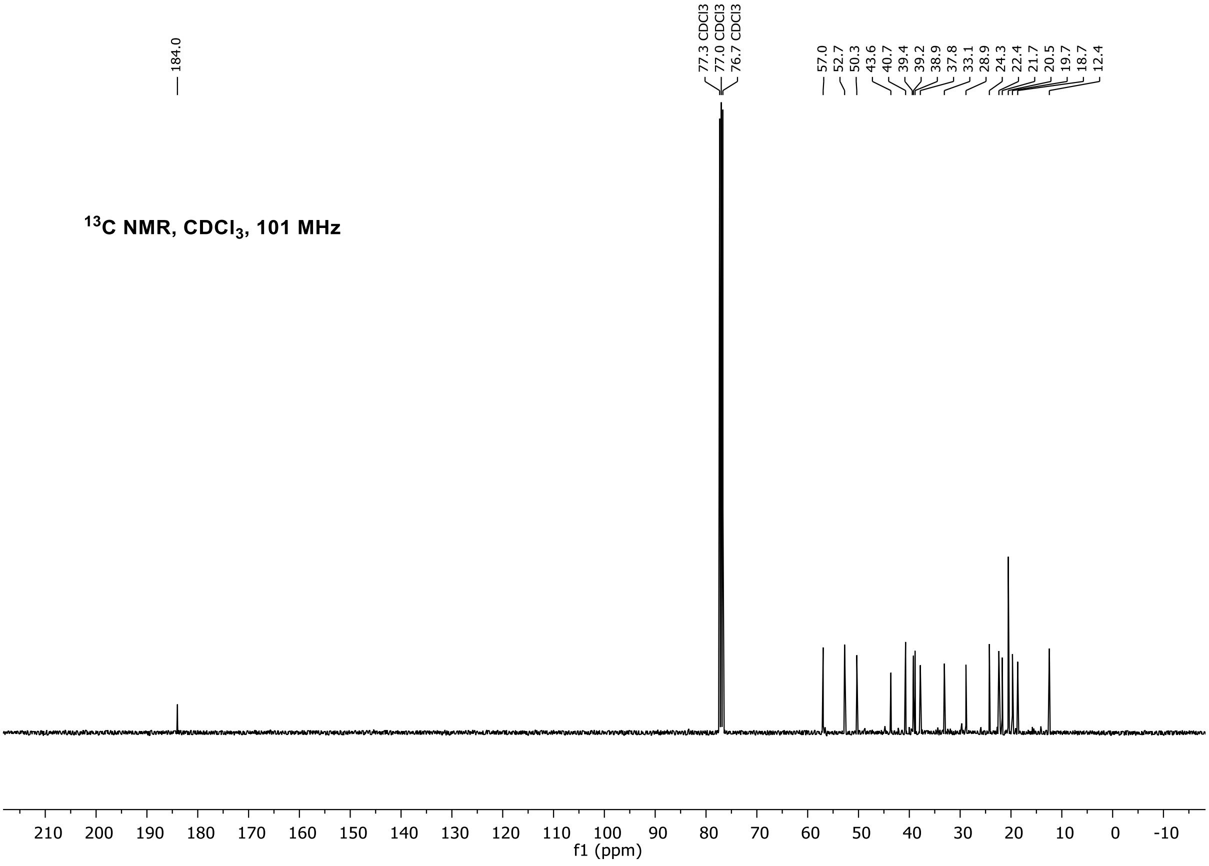

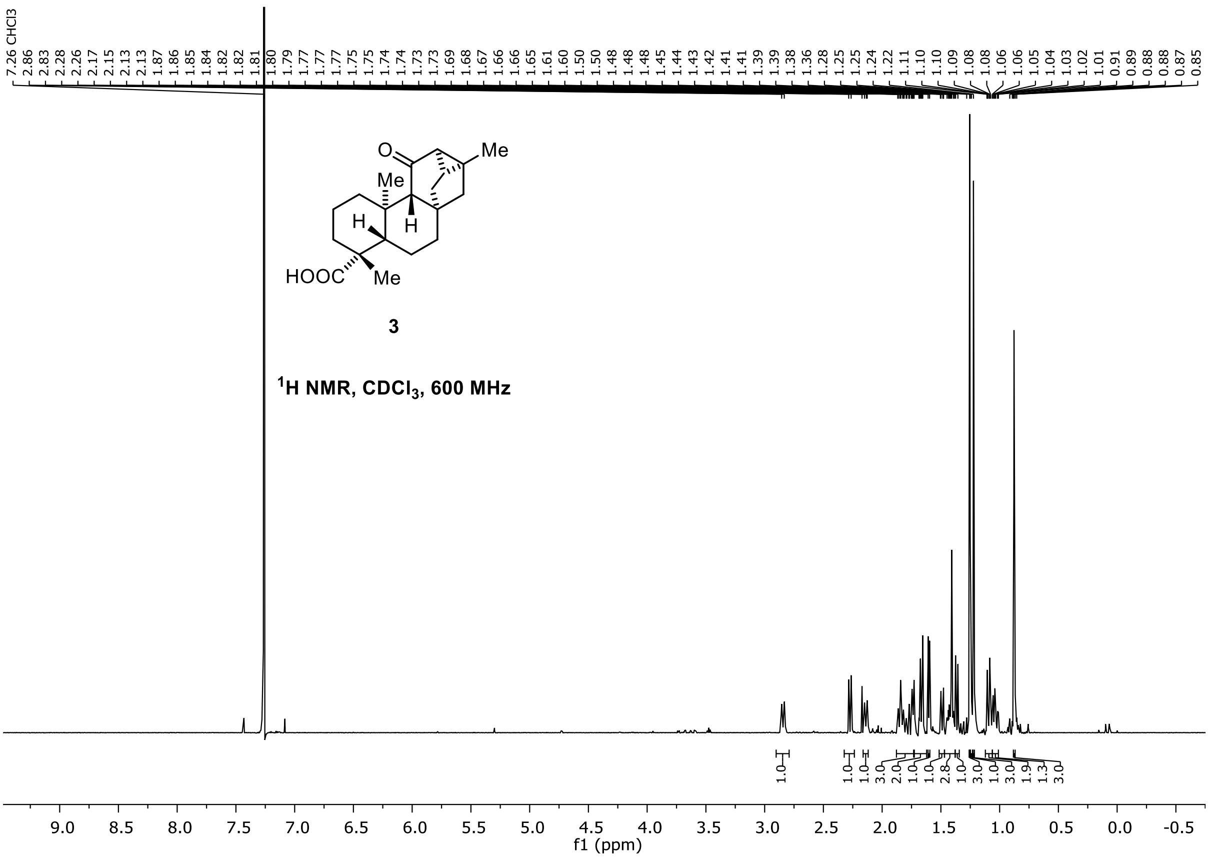
7.26 CHCl3
2.86 2.83 2.28 2.26 2.17 2.15 2.13 2.13 1.87 1.86 1.85 1.84 1.82 1.82 1.81 1.80 1.79 1.77 1.77 1.77 1.75 1.75 1.74 1.74 1.73 1.73 1.69 1.68 1.67 1.66 1.66 1.65 1.61 1.60 1.50 1.50 1.48 1.48 1.45 1.44 1.43 1.42 1.41 1.41 1.39 1.39 1.38 1.36 1.28 1.25 1.25 1.24 1.22 1.11 1.10 1.10 1.09 1.08 1.08 1.06 1.06 1.05 1.04 1.03 1.02 1.01 0.91 0.89 0.88 0.87 0.85
O
Me
Me
H
H
HOOC
Me
3
^{1}H NMR, $CDCl_3$, 600 MHz
1.0 1.0 1.0 3.0 2.0 1.0 1.0 2.8 1.0 3.0 1.0 3.0 1.9 1.3 3.0
9.0 8.5 8.0 7.5 7.0 6.5 6.0 5.5 5.0 4.5 4.0 3.5 3.0 2.5 2.0 1.5 1.0 0.5 0.0 -0.5
f1 (ppm)

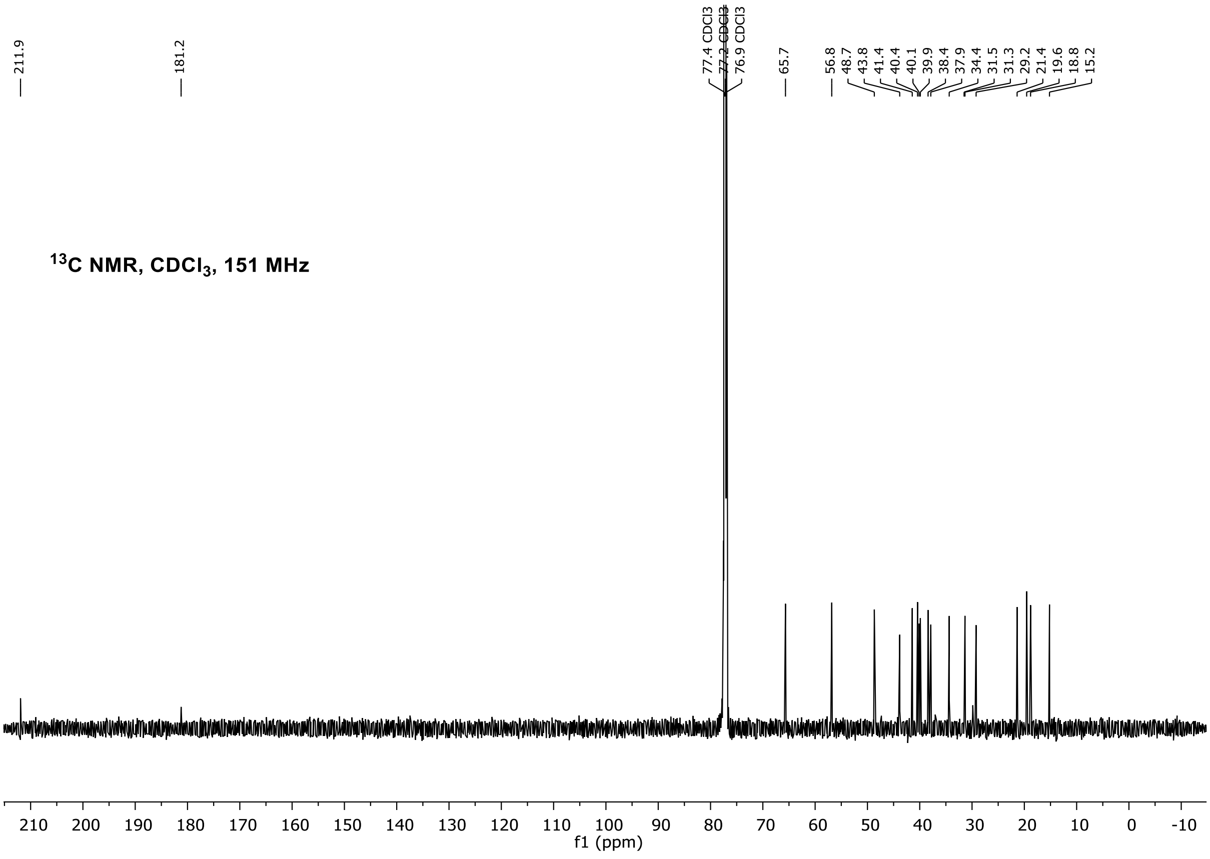
211.9
181.2
77.4 CDCl3
77.2 CDCl3
76.9 CDCl3
65.7
56.8 48.7 43.8 41.4 40.4 40.1 39.9 38.4 37.9 34.4 31.5 31.3 29.2 21.4 19.6 18.8 15.2
^{13}C NMR, $CDCl_3$, 151 MHz
210 200 190 180 170 160 150 140 130 120 110 100 90 80 70 60 50 40 30 20 10 0 -10
f1 (ppm)

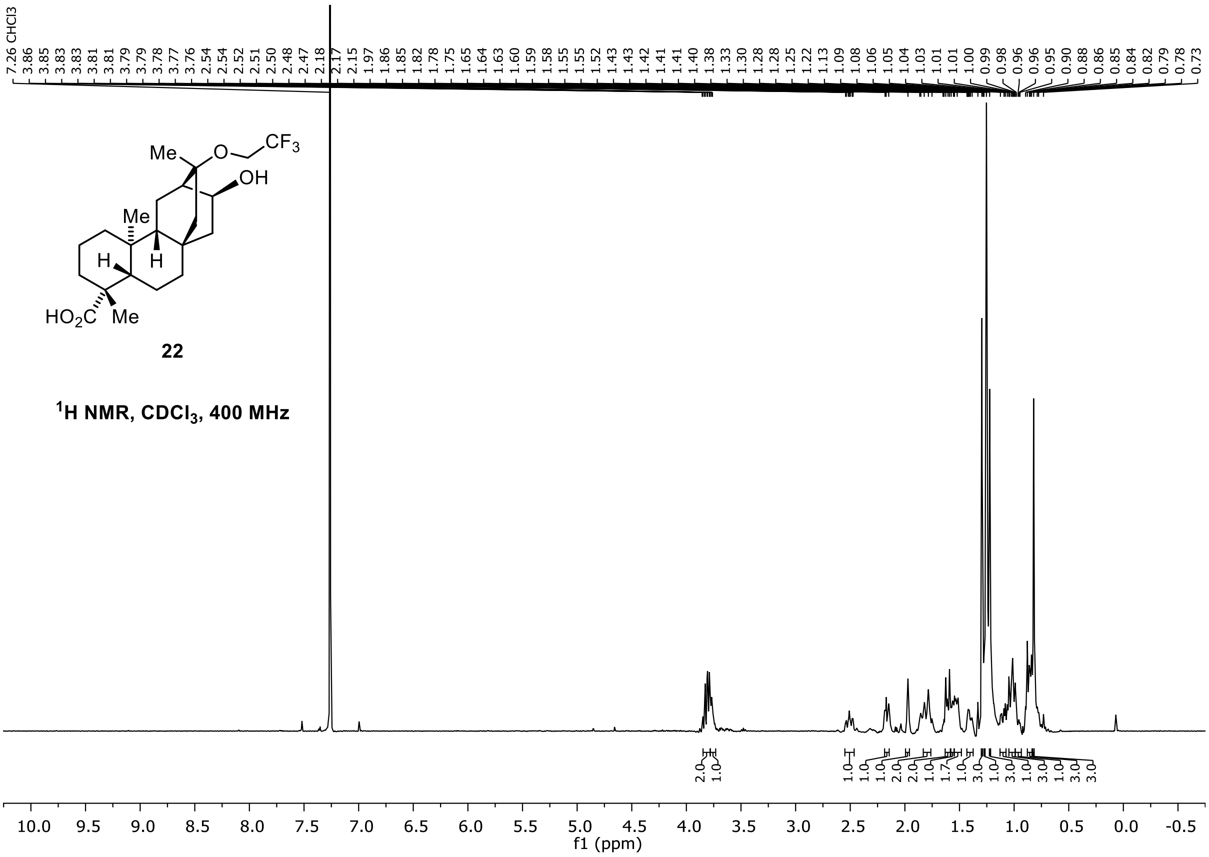

22

^{1}H NMR, $CDCl_3$, 400 MHz

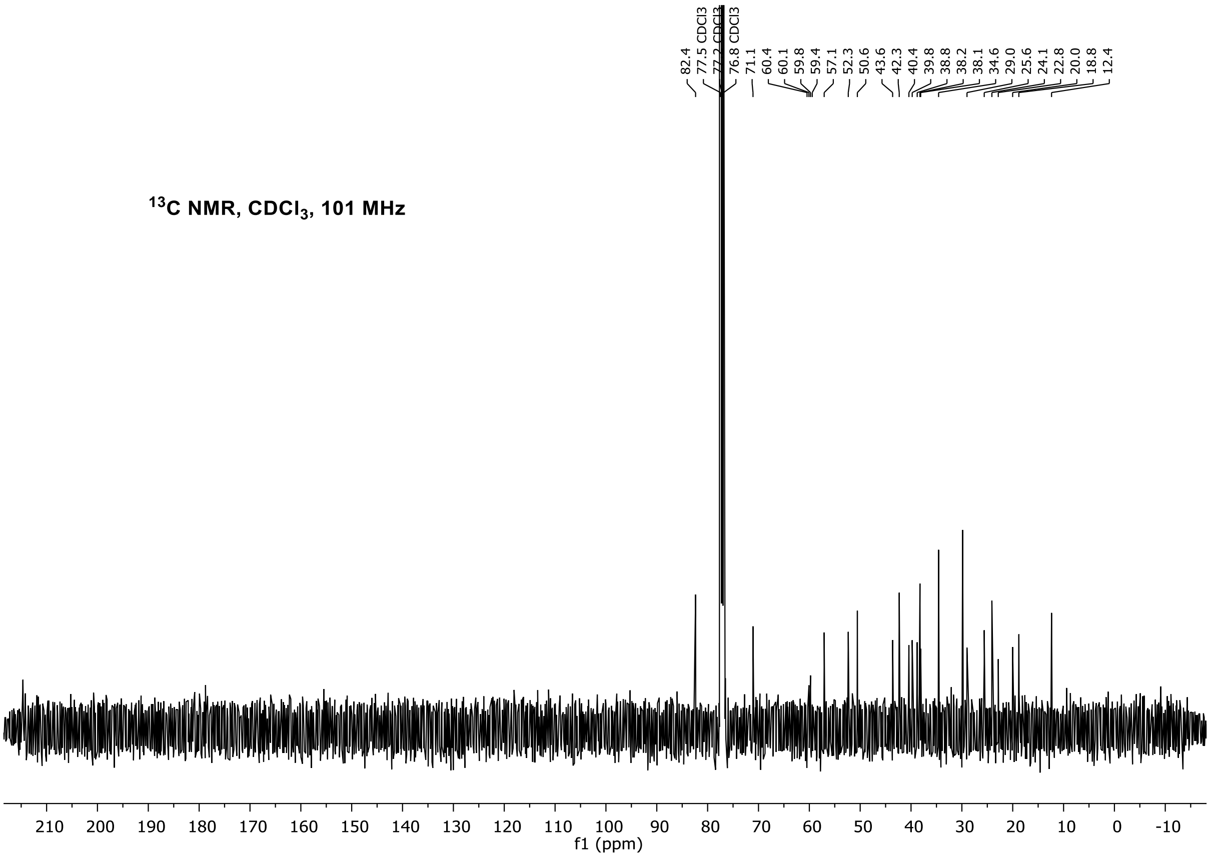

^{13}C NMR, $CDCl_3$, 101 MHz

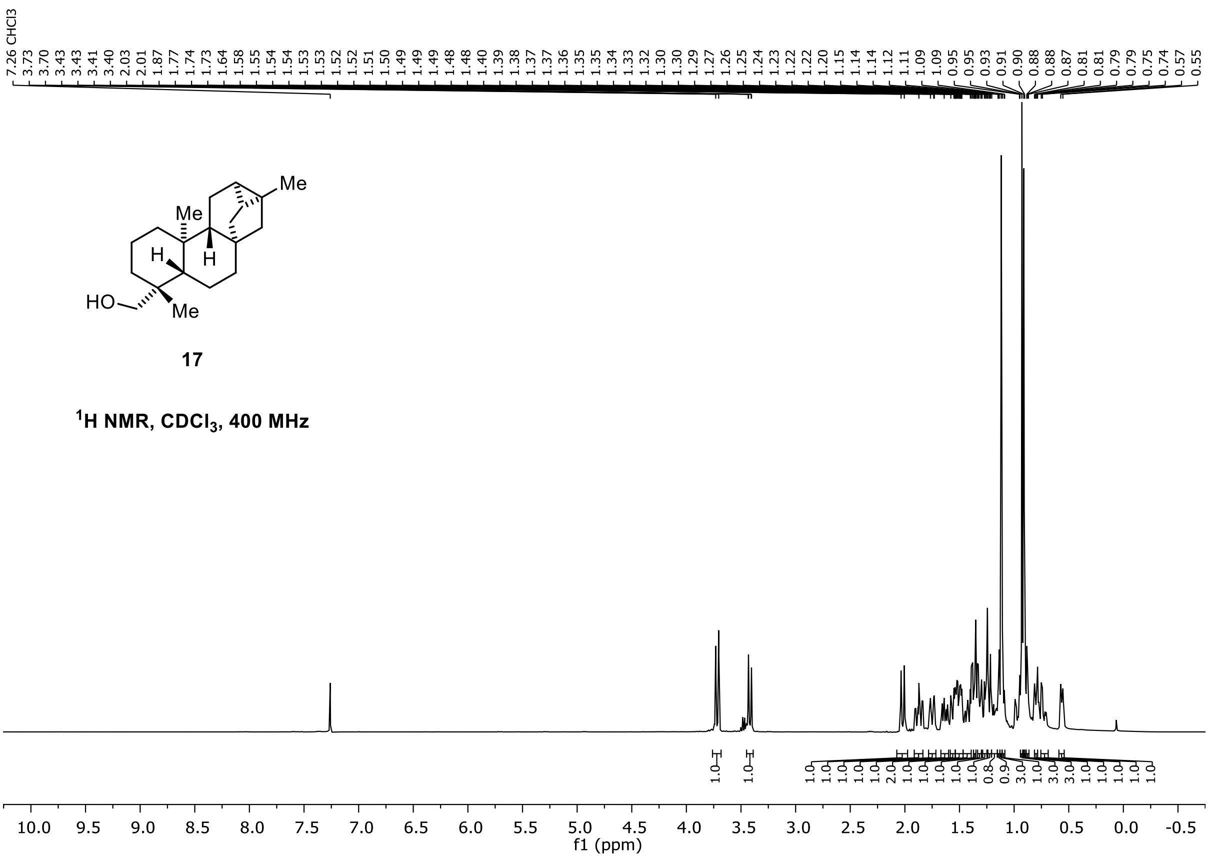

17

^{1}H NMR, CDCl$_3$, 400 MHz

^{13}C NMR, CDCl$_3$, 101 MHz

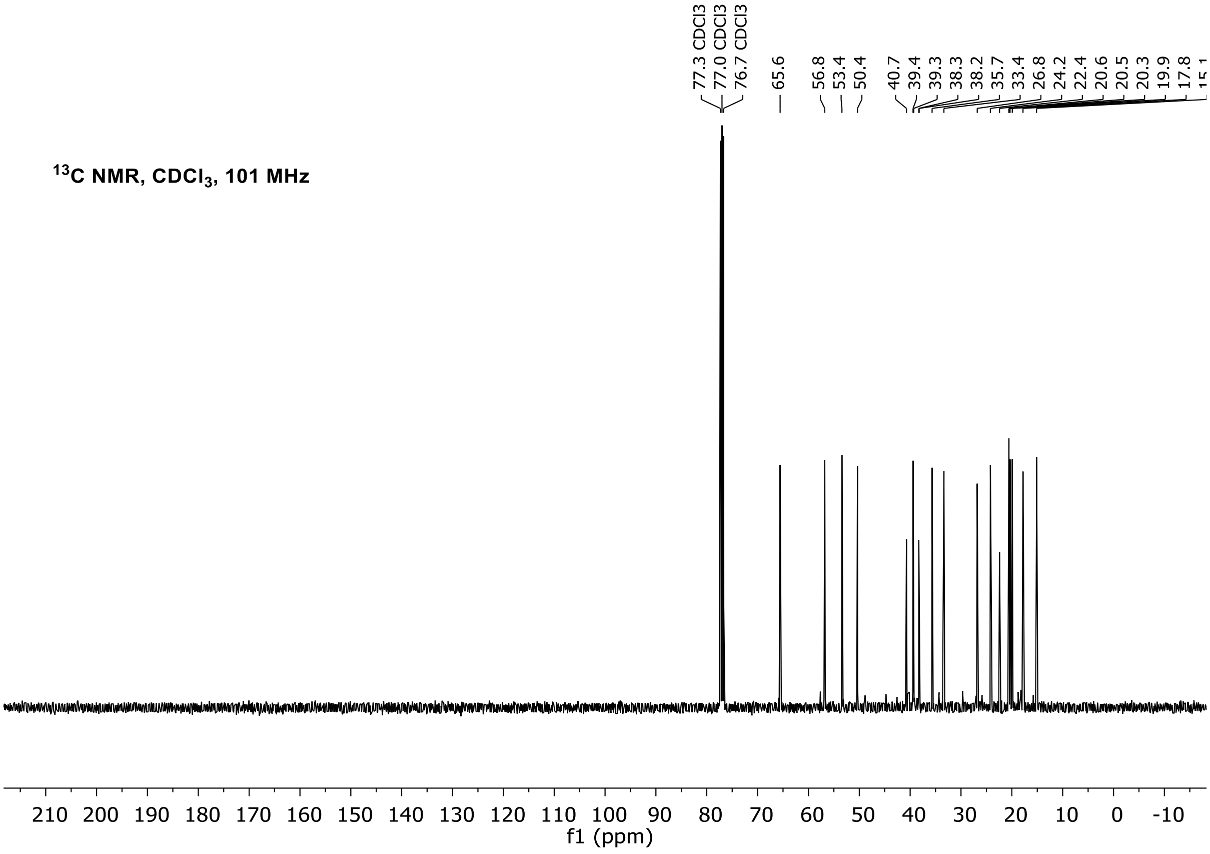

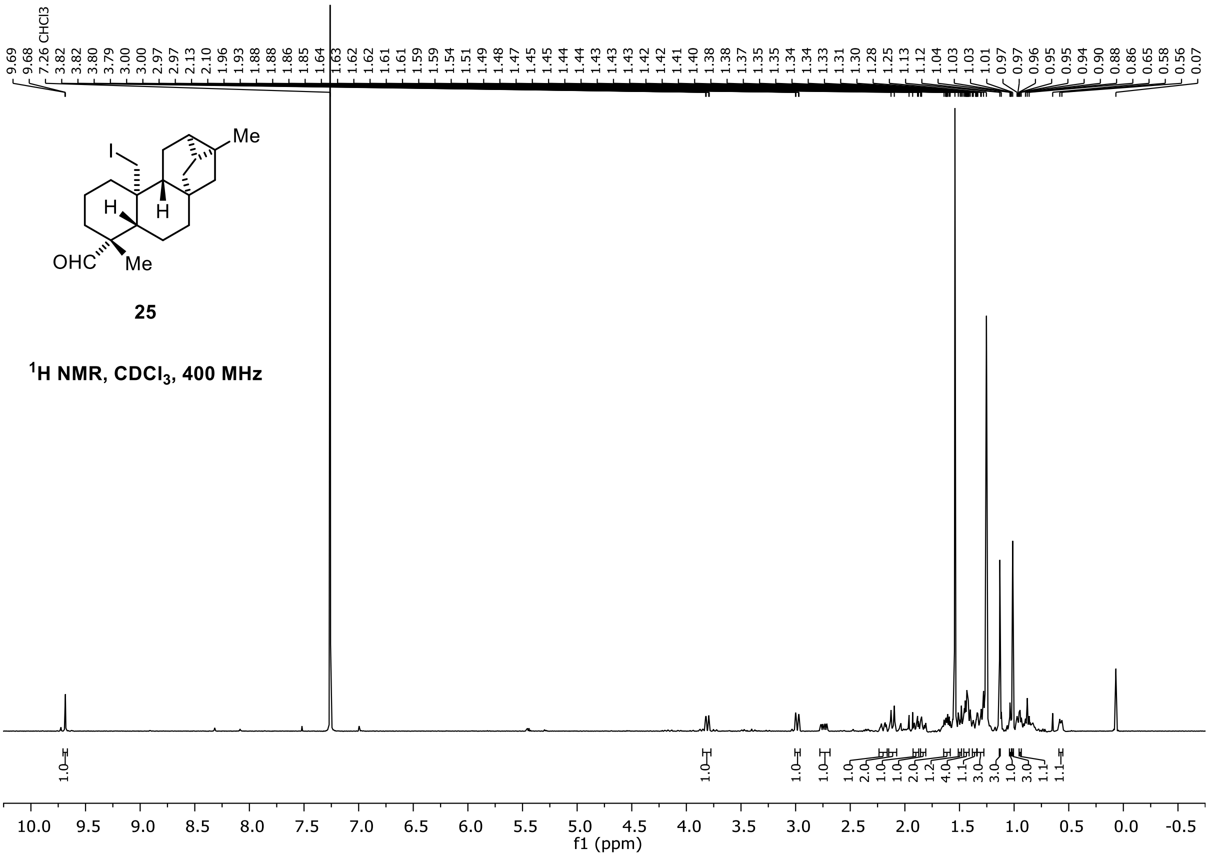

^{13}C NMR, CDCl$_3$, 101 MHz

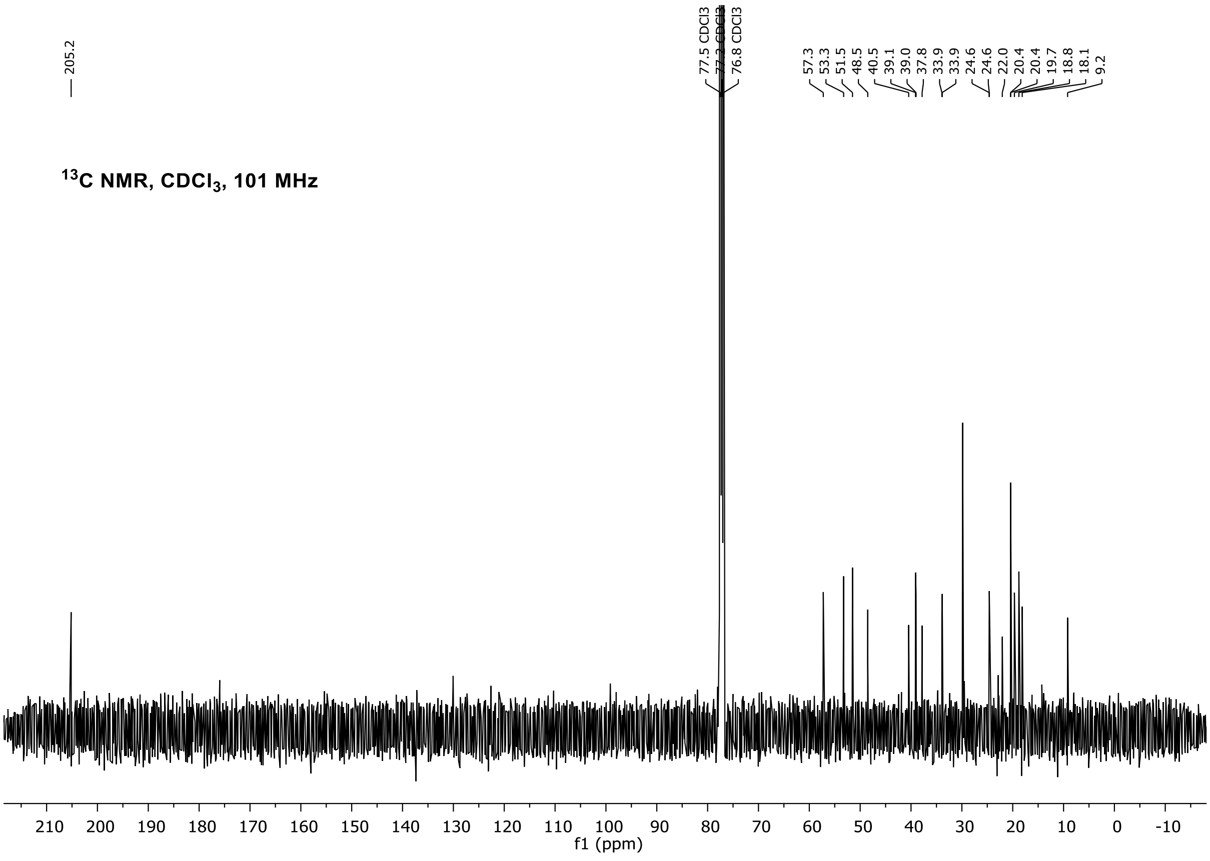

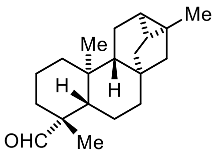

S-17

^{1}H NMR, $CDCl_3$, 400 MHz

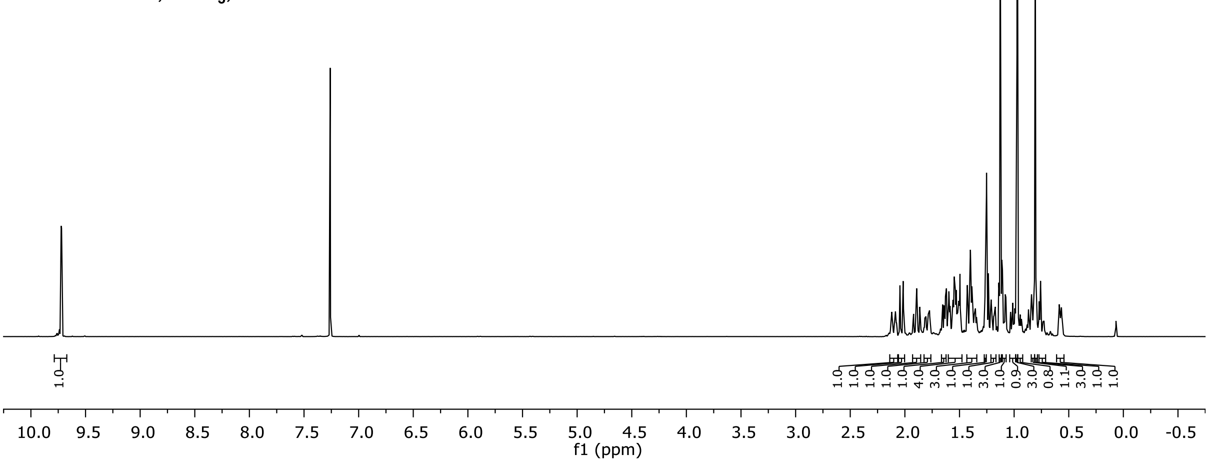

^{13}C NMR, $CDCl_3$, 101 MHz

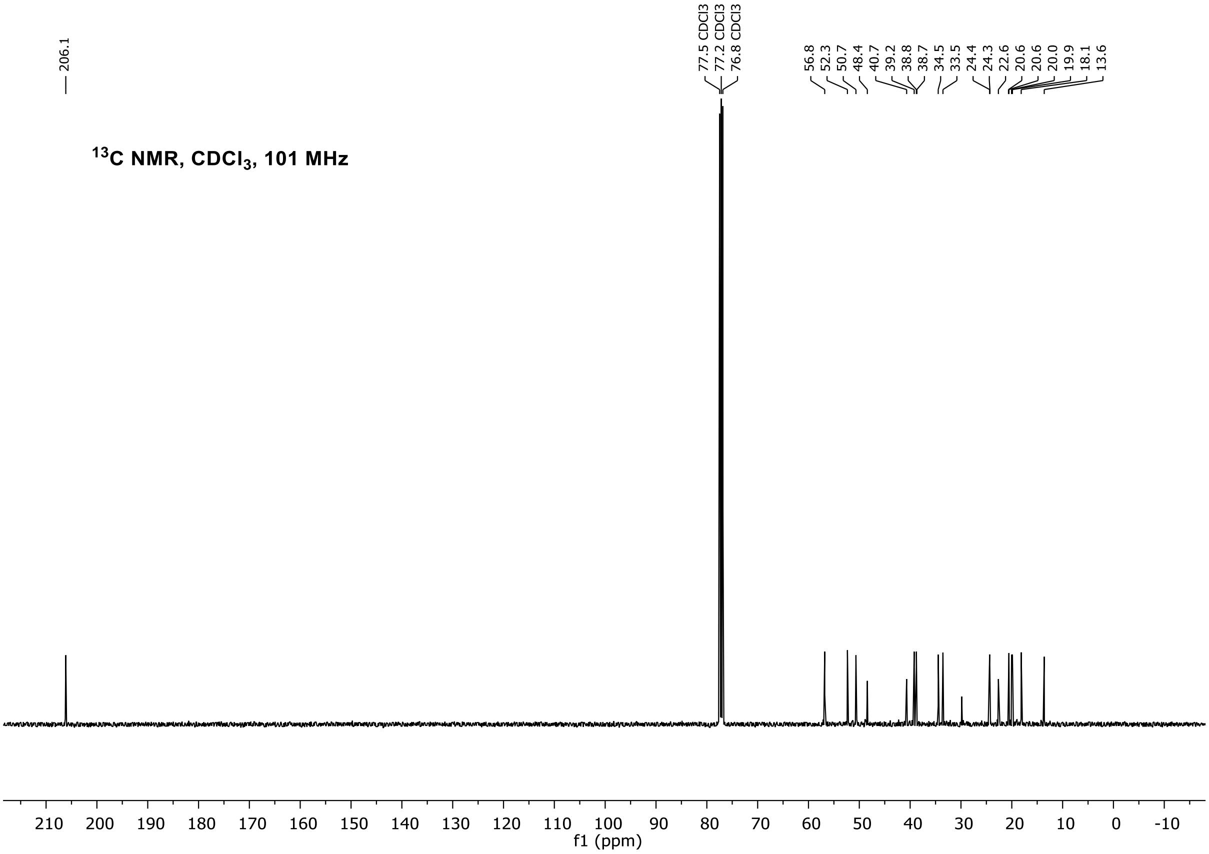

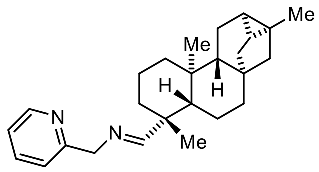

26

^{1}H NMR, CDCl$_3$, 400 MHz

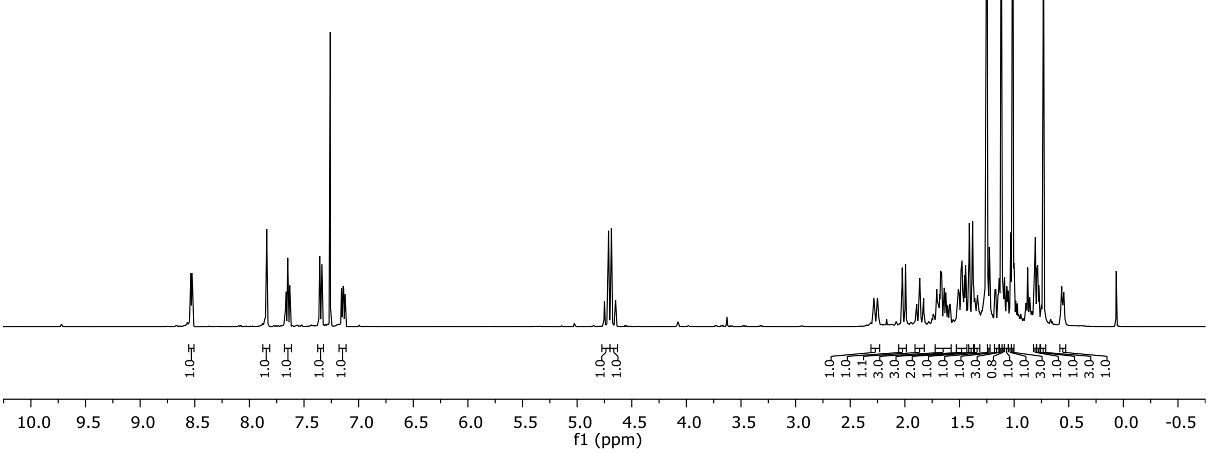

^{13}C NMR, CDCl$_3$, 101 MHz

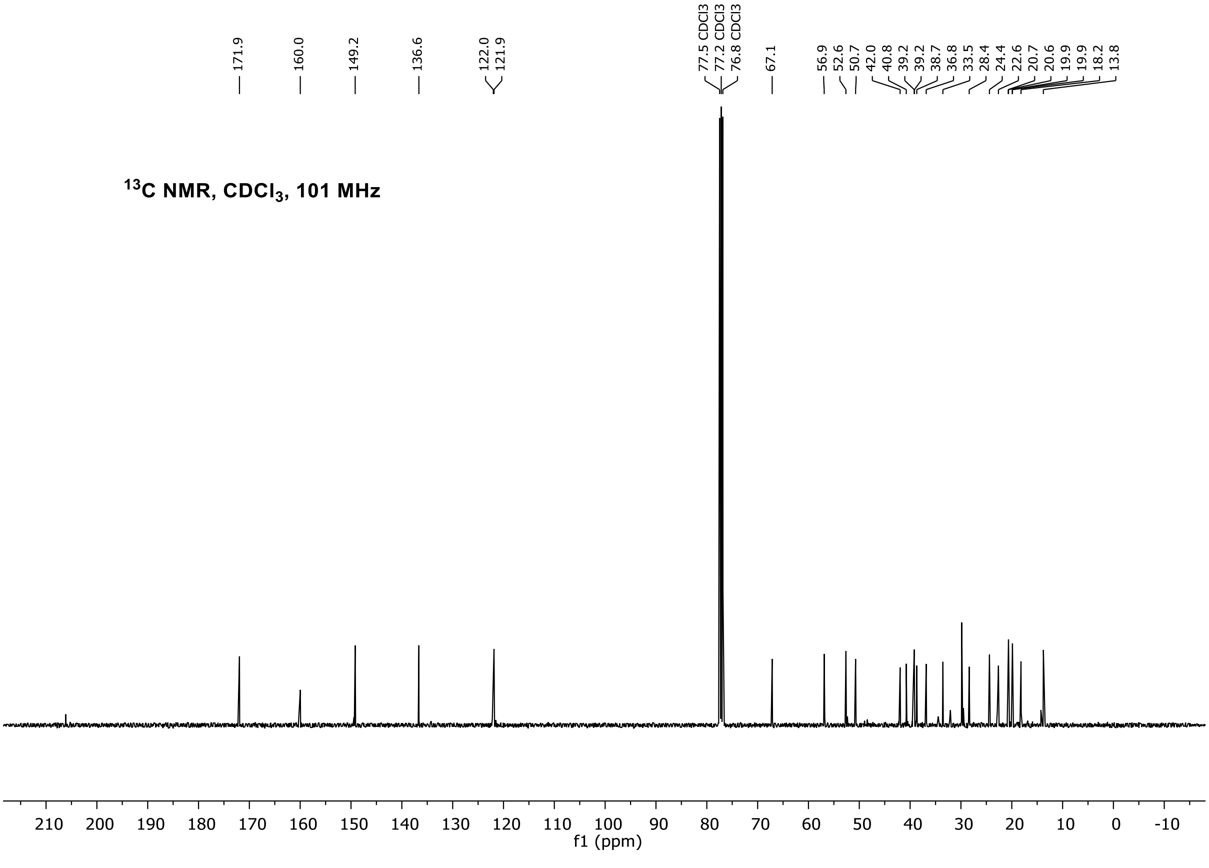

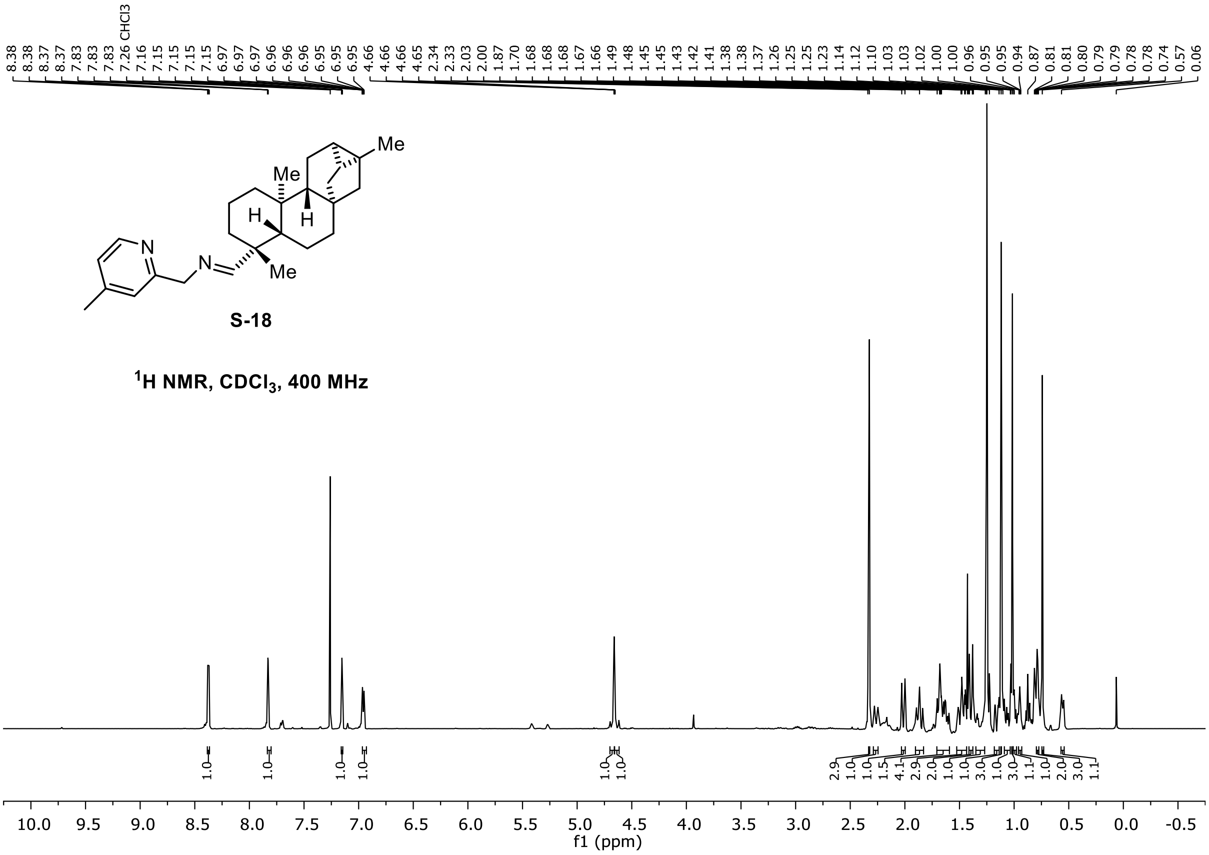

S-18

^{1}H NMR, $CDCl_3$, 400 MHz

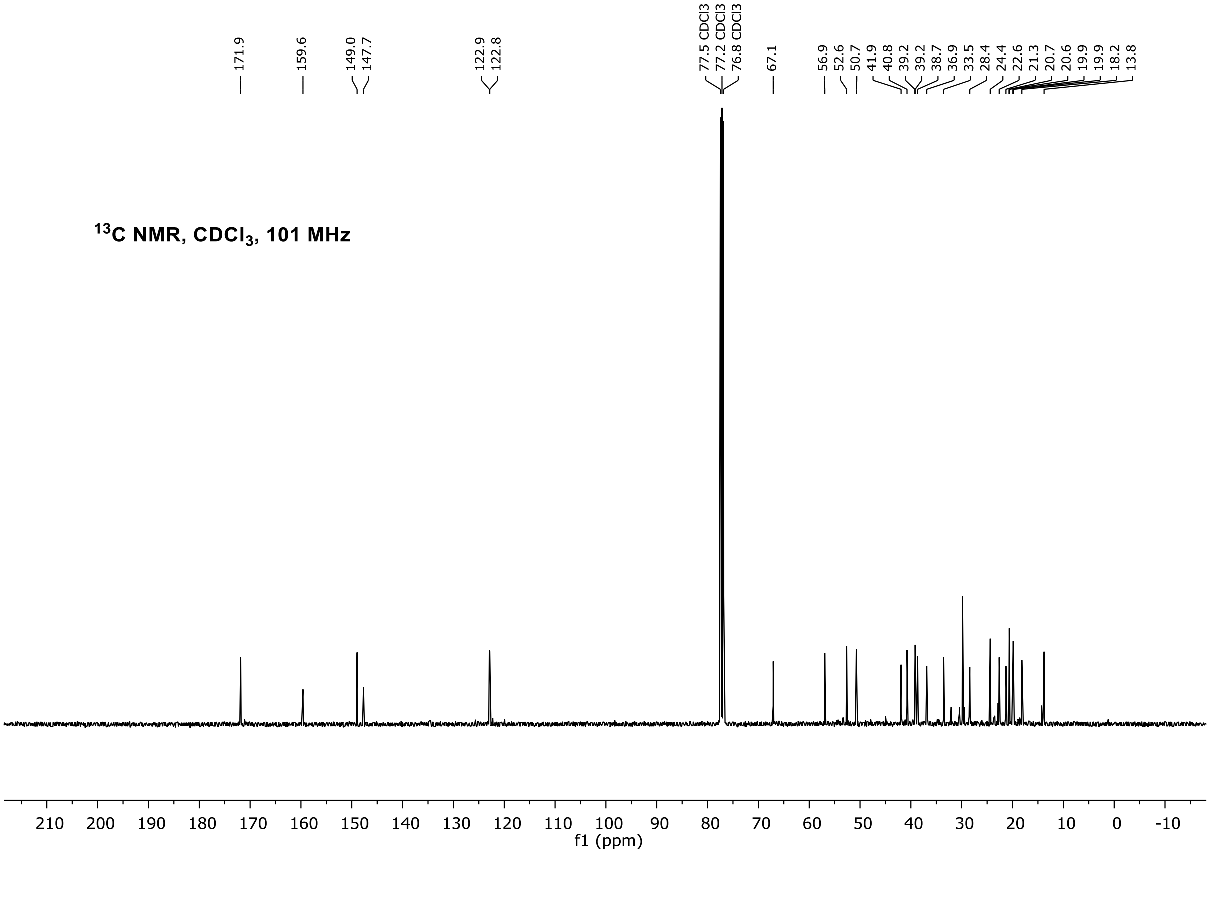

^{13}C NMR, $CDCl_3$, 101 MHz

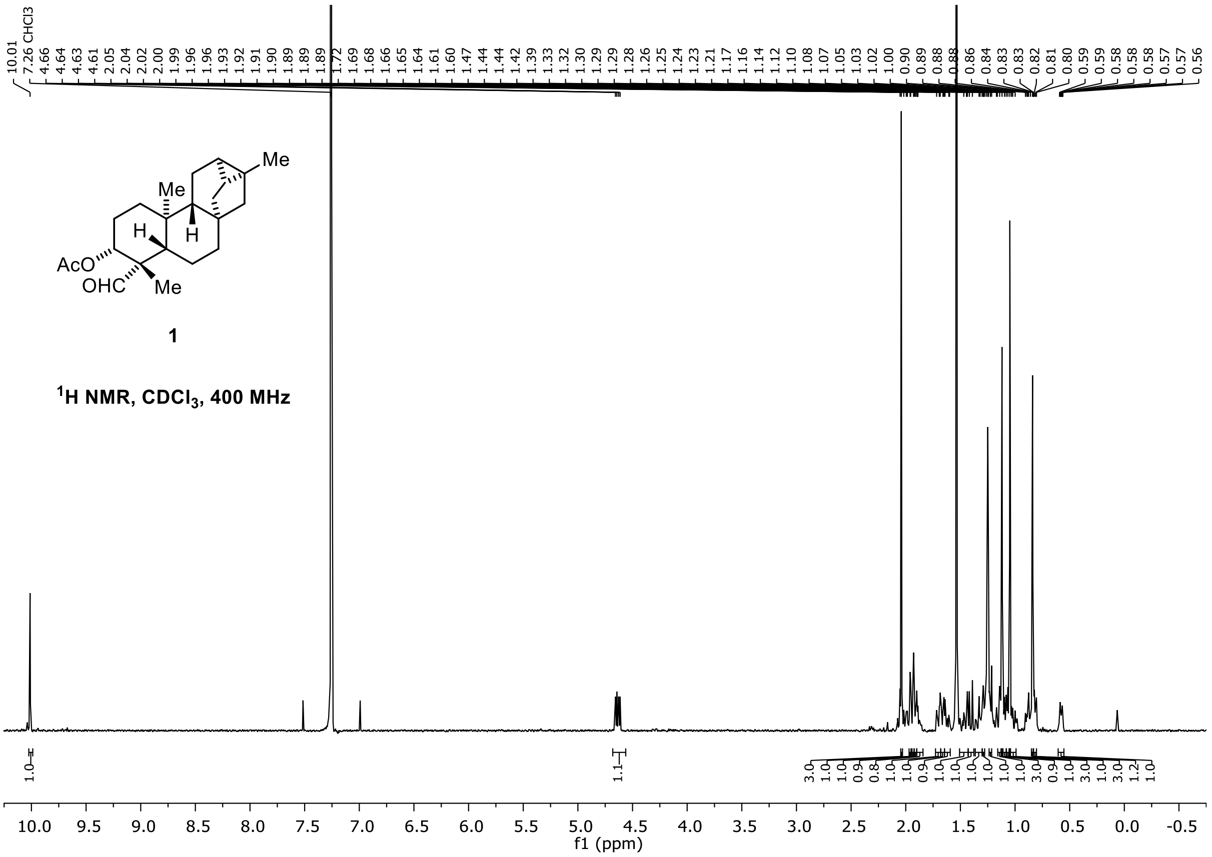

1

^{1}H NMR, CDCl$_3$, 400 MHz

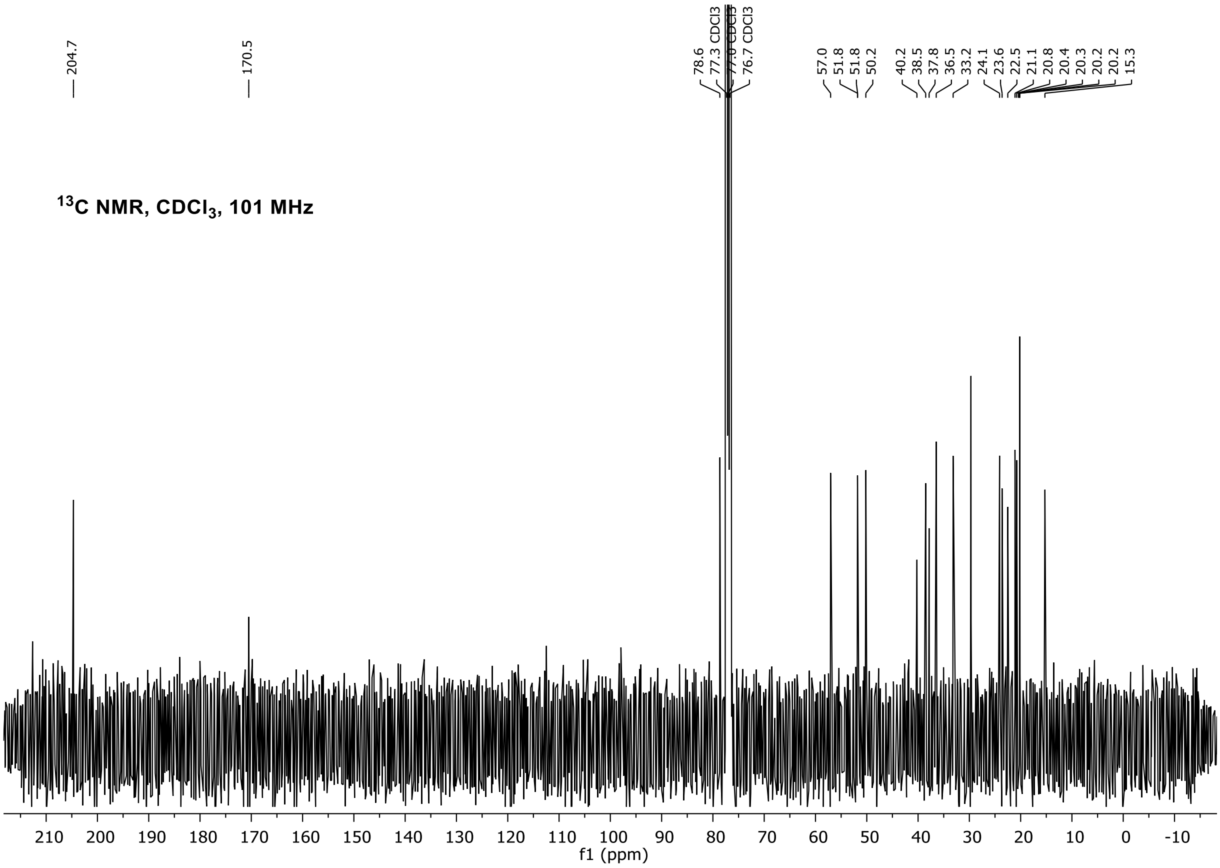

^{13}C NMR, CDCl$_3$, 101 MHz

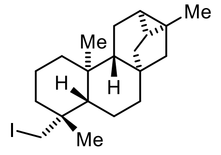

S-19

^{1}H NMR, $CDCl_3$, 400 MHz

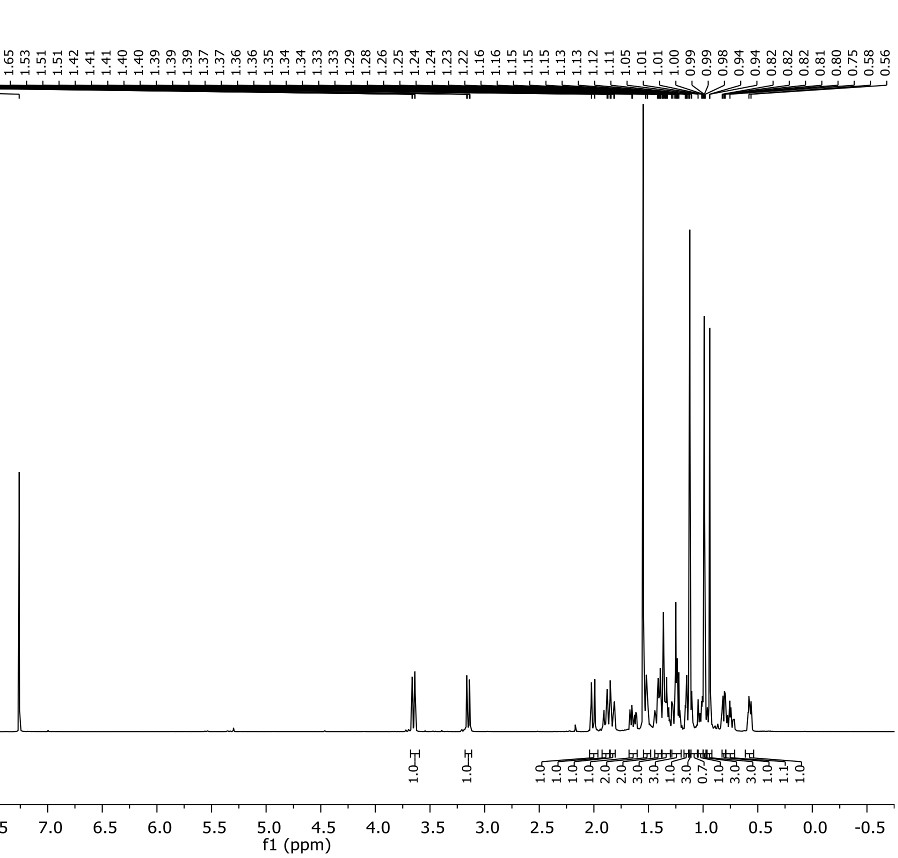

^{13}C NMR, $CDCl_3$, 101 MHz

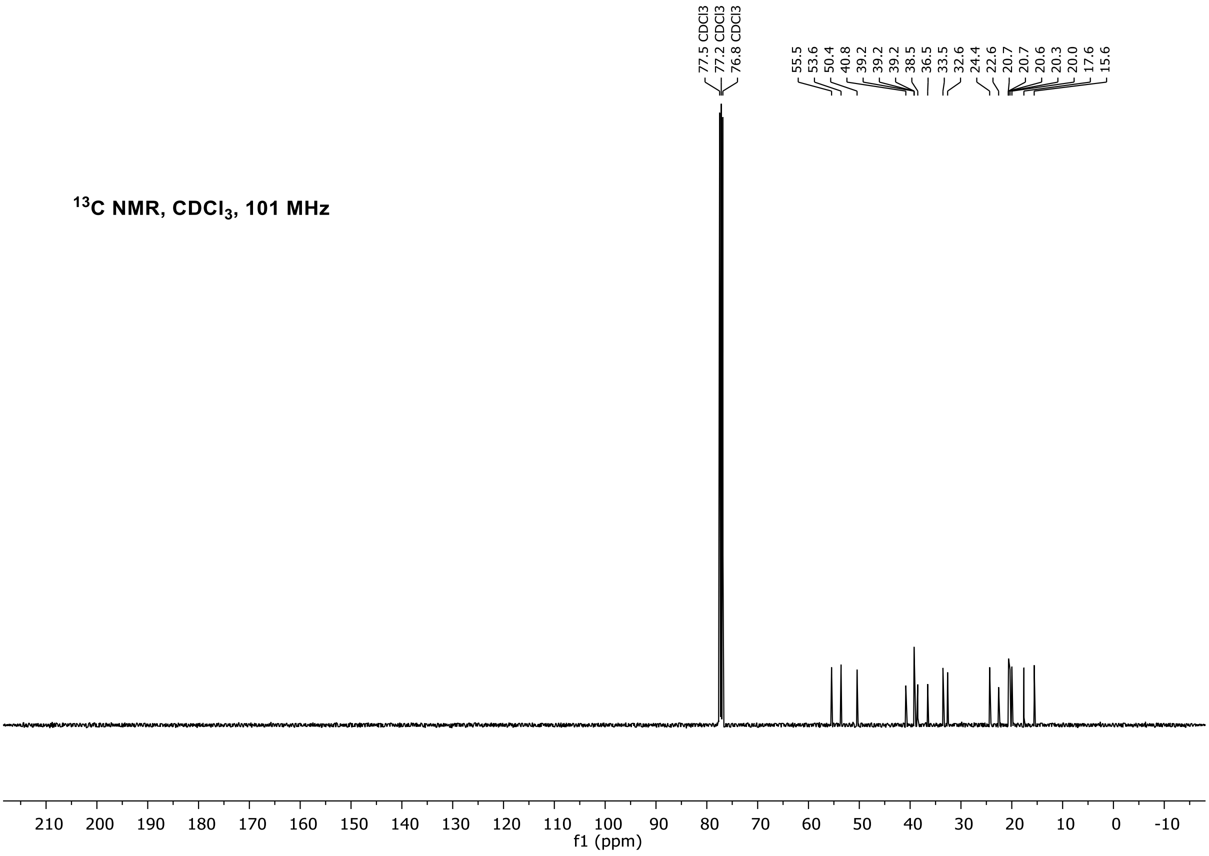

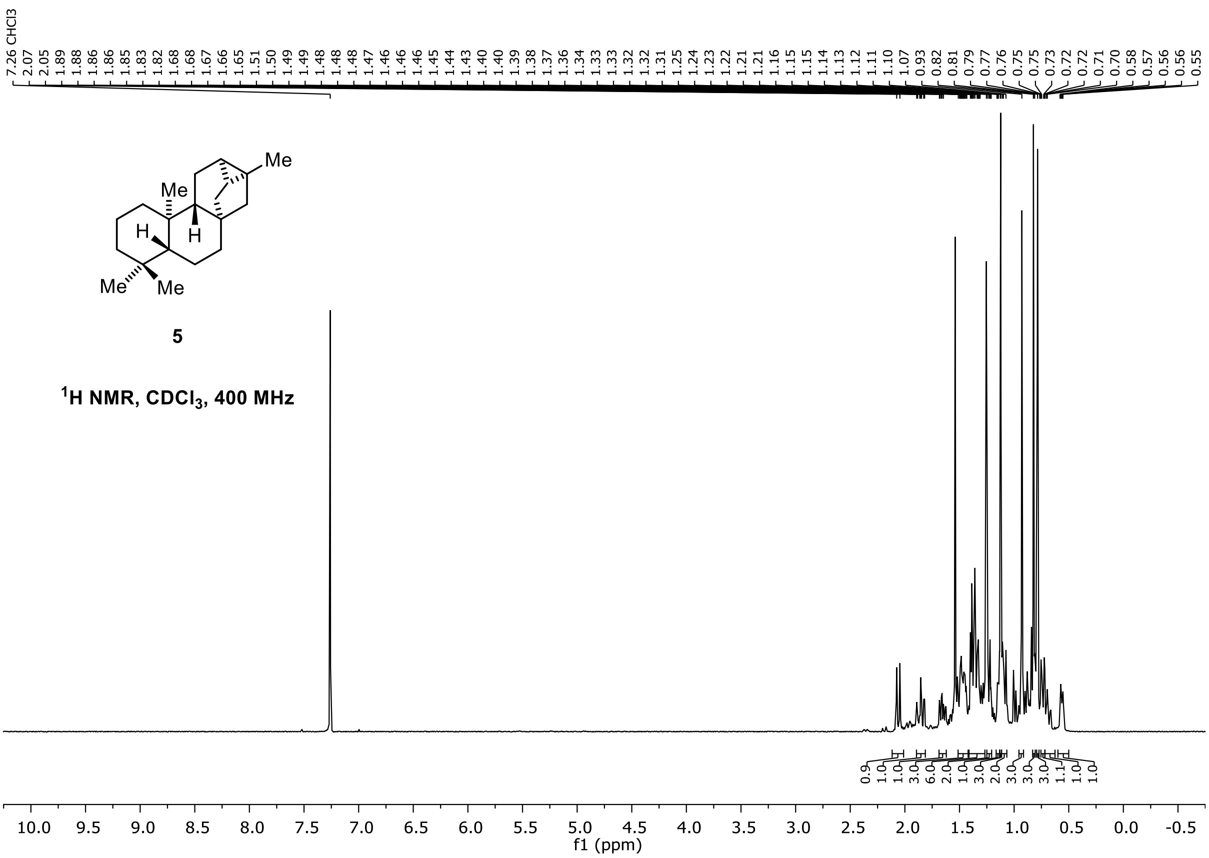
5
1H NMR, CDCl3, 400 MHz
f1 (ppm)

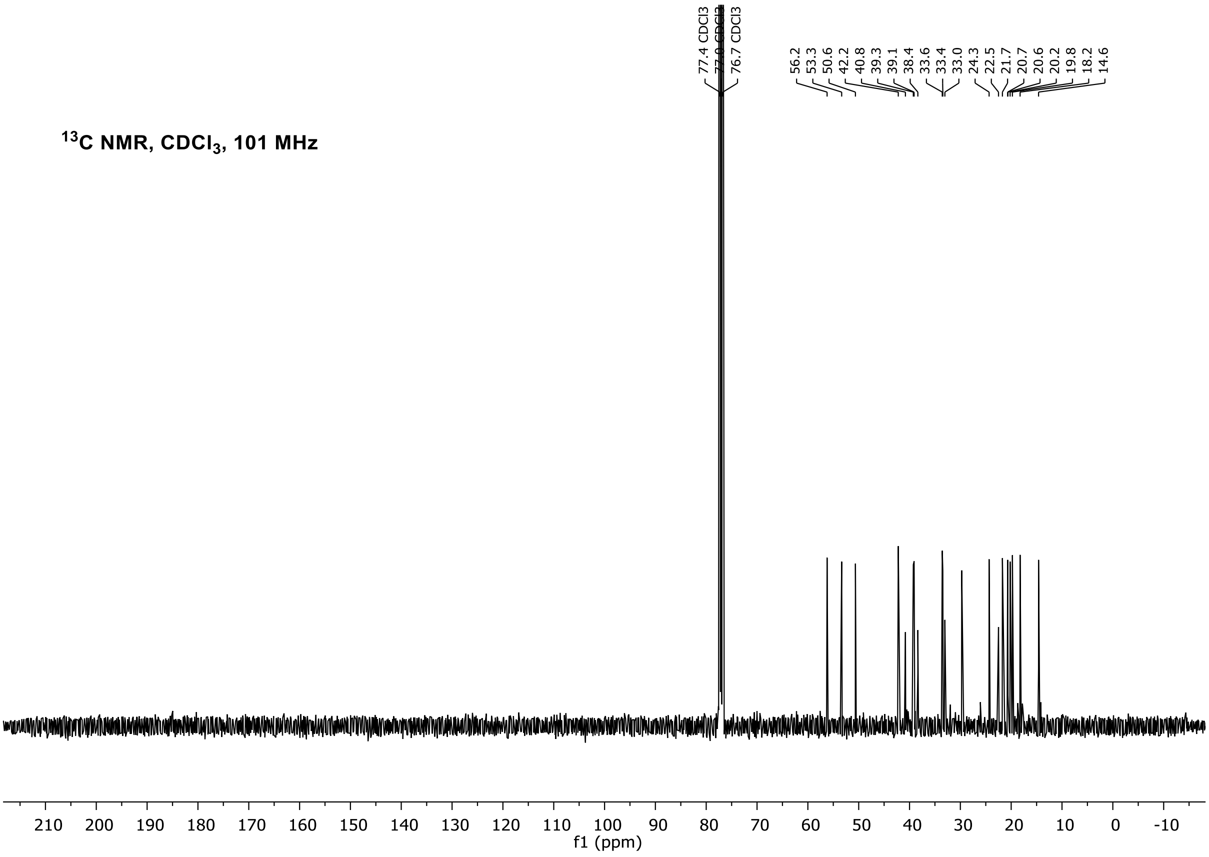
13C NMR, CDCl3, 101 MHz
77.4 CDCl3
77.0 CDCl3
76.7 CDCl3
56.2
53.3
50.6
42.2
40.8
39.3
39.1
38.4
33.6
33.4
33.0
24.3
22.5
21.7
20.7
20.6
20.2
19.8
18.2
14.6
f1 (ppm)

X-Ray Data

Carboxylic Acid 6

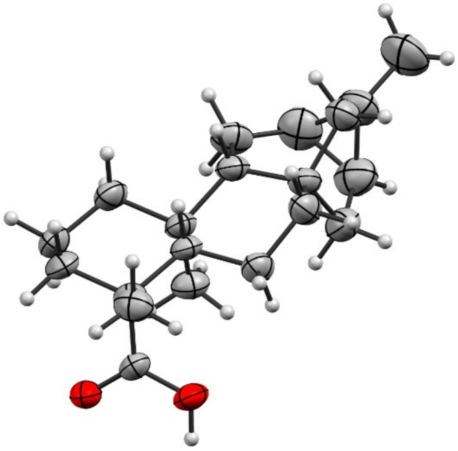

Identification code	I667
Empirical formula	C20 H30 O2
Formula weight	302.44
Temperature	193(2) K
Wavelength	1.54184 Å
Crystal system	Triclinic
Space group	P-1
Unit cell dimensions	a = 6.7083(9) Å
	b = 7.5468(10) Å
	c = 18.0182(14) Å
Volume	870.31(19) $Å^3$
Z	2
Density (calculated)	1.154 Mg/m^3
Absorption coefficient	0.557 mm-1
F(000)	332
Crystal size	0.019 x 0.016 x 0.009 mm^3
Theta range for data collection	2.457 to 62.201°.
Index ranges	-7<=h<=7, -8<=k<=8, -20<=l<=11
Reflections collected	3974
Independent reflections	2639 [R(int) = 0.0371]
Completeness to theta = 62.201°	96.00%
Absorption correction	Semi-empirical from equivalents
Max. and min. transmission	0.962 and 0.931
Refinement method	Full-matrix least-squares on F2
Data / restraints / parameters	2639 / 3 / 215
Goodness-of-fit on F2	1.043
Final R indices [I>2sigma(I)]	R1 = 0.0585, wR2 = 0.1470
R indices (all data)	R1 = 0.0841, wR2 = 0.1750
Extinction coefficient	0.0068(14)
Largest diff. peak and hole	0.204 and -0.200 e.$Å^{-3}$

Secondary Alcohol 22

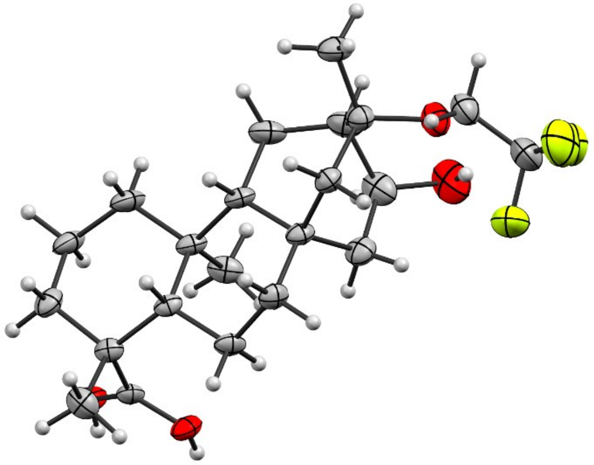

Identification code	mar21-11	
Empirical formula	C22 H33 F3 O4	
Formula weight	418.48	
Temperature	153(2) K	
Wavelength	0.71073 Å	
Crystal system	Monoclinic	
Space group	C2 (no. 5)	
Unit cell dimensions	a = 11.7589(6) Å	a= 90°
	b = 8.3291(4) Å	b= 100.463(2)°
	c = 21.6350(11) Å	g = 90°
Volume	2083.72(18) Å^3	
Z	4	
Density (calculated)	1.334 Mg/m^3	
Absorption coefficient	0.107 mm^{-1}	
F(000)	896	
Crystal size	0.160 x 0.080 x 0.040 mm^3	
Theta range for data collection	1.914 to 24.999°	
Index ranges	-13<=h<=13, -9<=k<=9, -25<=l<=25	
Reflections collected	17742	
Independent reflections	3658 [R(int) = 0.0423]	
Completeness to theta = 24.999°	99.90%	
Absorption correction	Semi-empirical from equivalents	
Max. and min. transmission	0.990 and 0.974	
Refinement method	Full-matrix least-squares on F$_2$	
Data / restraints / parameters	3658 / 4 / 278	
Goodness-of-fit on F2	1.073	
Final R indices [I>2sigma(I)]	R1 = 0.0380, wR2 = 0.0776	
R indices (all data)	R1 = 0.0492, wR2 = 0.0809	
Absolute structure parameter	1.6(3)	
Extinction coefficient	0.0067(9)	
Largest diff. peak and hole	0.181 and -0.128 e.Å^{-3}	

Chiral GC Data

Alcohol S-7.

Chiral gas chromatography (GC) was performed on the following column: Chirasil-Dex CB, Varian, CP7502 (25.0 m × 250 um x 0.25 um), Average velocity 20, H2-flux, T = 170 °C.

```
ata File C:\HPCHEM\1\DATA\LW22CA-1.D                              Sample Name: LW22CA
 170° 30 min, 1.2 flow

=====================================================================
Injection Date  : 13.08.2019 08:35:54              Seq. Line :   1
Sample Name     : LW22CA                            Location : Vial 1
Acq. Operator   : Peter                                  Inj :   1
                                                  Inj Volume : Manually
Sequence File   : C:\HPCHEM\1\SEQUENCE\Y04.S
Method          : C:\HPCHEM\1\METHODS\JURIA1LW.M
Last changed    : 13.08.2019 08:34:58  by Peter
```

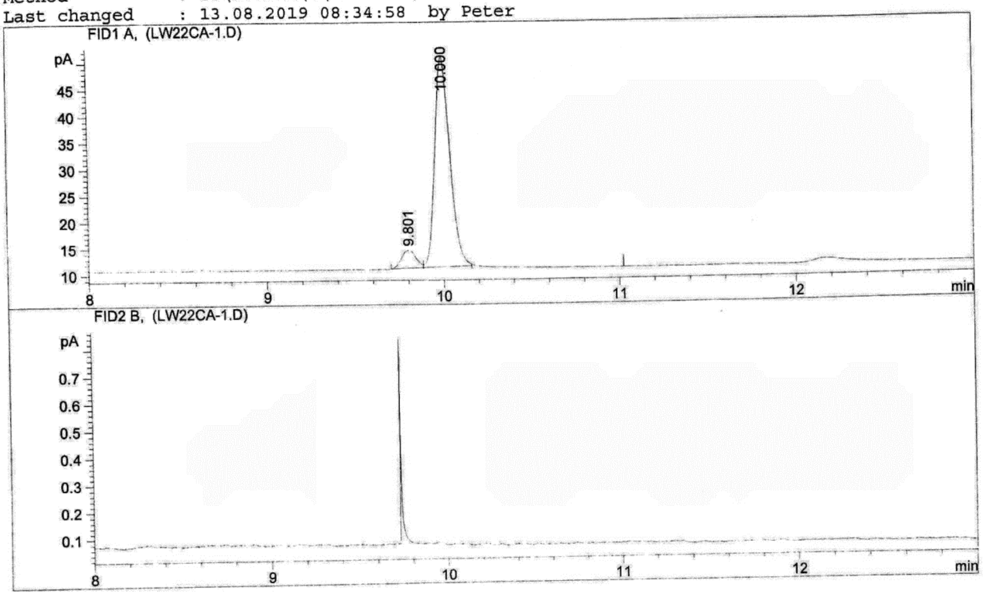


```
=====================================================================
                        Area Percent Report
=====================================================================

Sorted By             :        Signal
Multiplier            :        1.0000
Dilution              :        1.0000
Use Multiplier & Dilution Factor with ISTDs


Signal 1: FID1 A,

Peak RetTime Type  Width      Area       Height     Area
  #   [min]        [min]     [pA*s]       [pA]        %
----|-------|----|-------|----------|----------|--------|
  1   9.801 PV    0.0862   19.39958    3.42100  7.61490
  2  10.000 VB    0.0835  235.35851   39.53039 92.38510

Totals :                  254.75809   42.95139

 Results obtained with enhanced integrator!

Signal 2: FID2 B,

=====================================================================
                        *** End of Report ***



GC1 (Chiral) 13.08.2019 09:43:52  Peter                    Page 1 of 1
```

References

[1] Deschamp, J.; Hermant, T.; Riant, O. An easy route toward enantio-enriched polycyclic derivatives via an asymmetric domino conjugate reduction–aldol cyclization catalyzed by a chiral Cu(I) complex. *Tetrahedron* **2012**, *68*, 3457–3467.

[2] Liu, J.; Ma, D. A Unified Approach for the Assembly of Atisine- and Hetidine-type Diterpenoid Alkaloids: Total Syntheses of Azitine and the Proposed Structure of Navirine C. *Angew. Chem. Int. Ed.* **2018**, *57,* 6676–6680.

[3] Bohlmann, F.; Jakupovic, J.; Ahmed, M.; Grenz, M.; Suding, H.; Robinson, H.; King, R. M. Germacranolides and Diteroenes from Viguiera Species. *Phytochemistry* **1981**, *20*, 113–116.

[4] Diderot, N. T.; Silvere, N.; Yasin, A.; Zareen, S.; Fabien, Z.; Etienne, T.; Choudhary, M. I.; Atta-ur-Rahman. Prolyl Endopeptidase and Thrombin Inhibitory Diterpenoids from the Bark of Xylopia aethiopica. *Biosci. Biotechnol. Biochem.* **2005**, *69*, 9, 1763–1766.

[5] Arnone, A.; Mondelli, R.; Pyrek, J. St. ^{13}C NMR Studies of Trachylobane Diterpenes: Complete Carbon Assignment. *Organic Magnetic Resonance*, **1979**, *12*, 7, 429–431.

[6] a) http://openflask.blogspot.com/2014/01/tfdo-synthesis-procedure.html. b) Adam, W.; van Barneveld, C.; Golsch, D. Direct synthesis of 3-aryl-1,2,4,5-tetrazine N-1-oxides by the oxidation with methyl(trifluoromethyl)dioxirane. *Tetrahedron*, *52*, 7, 2377–2384.

[7] Herz, W.; Kulanthaivel, P. Ent-kauranes and trachylobanes from Helianthus radula. *Phytochemistry*, **1983**, *22*, 11, 2543–2546.

[8] Ngamrojnavanich, N.; Tonsiengsom, S.; Lertpratchya, P.; Roengsumran, S.; Puthong, S.; Petsom, A. Diterpenoids from the Stem Barks of Croton robustus. *Arch. Pharm. Res.* **2003**, *26*, 11, 898–901.

[9] Scher, J. M.; Schinkovitz, A.; Zapp, J.; Wang, Y.; Franzblau, S. G.; Becker, H.; Lankin, D. C.; Pauli, G. F. Structure and Anti-TB Activity of Trachylobanes from the Liverwort Jungermannia exsertifolia ssp. cordifolia. *J. Nat. Prod.* **2010**, *73*, 4, 656–663.

[10] Briggs, L. H.; White, G. W. Constitutents of the Essential Oil of Araucauria Araucana. *Tetrahedron* **1975**, 31, 1311–1314.

[11] Pyrek, J. St. New Pentacyclic Diterpene Acid Trachyloban-19-oic Acid from Sunflower. *Tetrahedron* **1970**, *26*, 5029–5032.

[12] Gaussian 16, Revision C.01, Frisch, M. J.; Trucks, G. W.; Schlegel, H. B.; Scuseria, G. E.; Robb, M. A.; Cheeseman, J. R.; Scalmani, G.; Barone, V.; Petersson, G. A.; Nakatsuji, H.; Li, X.; Caricato, M.; Marenich, A. V.; Bloino, J.; Janesko, B. G.; Gomperts, R.; Mennucci, B.; Hratchian, H. P.; Ortiz, J. V.; Izmaylov, A. F.; Sonnenberg, J. L.; Williams-Young, D.; Ding, F.;

Lipparini, F.; Egidi, F.; Goings, J.; Peng, B.; Petrone, A.; Henderson, T.; Ranasinghe, D.; Zakrzewski, V. G.; Gao, J.; Rega, N.; Zheng, G.; Liang, W.; Hada, M.; Ehara, M.; Toyota, K.; Fukuda, R.; Hasegawa, J.; Ishida, M.; Nakajima, T.; Honda, Y.; Kitao, O.; Nakai, H.; Vreven, T.; Throssell, K.; Montgomery, J. A., Jr.; Peralta, J. E.; Ogliaro, F.; Bearpark, M. J.; Heyd, J. J.; Brothers, E. N.; Kudin, K. N.; Staroverov, V. N.; Keith, T. A.; Kobayashi, R.; Normand, J.; Raghavachari, K.; Rendell, A. P.; Burant, J. C.; Iyengar, S. S.; Tomasi, J.; Cossi, M.; Millam, J. M.; Klene, M.; Adamo, C.; Cammi, R.; Ochterski, J. W.; Martin, R. L.; Morokuma, K.; Farkas, O.; Foresman, J. B.; Fox, D. J. Gaussian, Inc., *Wallingford CT*, **2016**.

[13] Chai, J.-D.; Head-Gordon, M. Long-range corrected hybrid density functionals with damped atom–atom dispersion corrections. *Phys. Chem. Chem. Phys.* **2008**, *10*, 6615–6620.

[14] Ditchfield, R., Hehre W. J.; Pople, J. A. Self-Consistent Molecular-Orbital Methods. IX. An Extended Gaussian-Type Basis for Molecular-Orbital Studies of Organic Molecules. *J. Chem. Phys.* **1971**, *54*, 724–728.

[15] Miertuš, S.; Tomasi, J. Approximate evaluations of the electrostatic free energy and internal energy changes in solution processes. *Chem. Phys.* **1982**, *65*, 239–245.

[16] Miertuš, S., Scrocco, E.; Tomasi, J. Electrostatic interaction of a solute with acontinuum. A direct utilization of AB initio molecular potentials for the prevision of solvent effects. *Chem. Phys.* **1981**, *55*, 117–129.

[17] Pascual-Ahuir, L., Silla, E.; Tuñón, I. GEPOL: An improved description of molecular surfaces. III. A new algorithm for the computation of a solvent-excluding surface. *J. Comp. Chem.* **1994**, *15*, 1127–1138.

[18] Legault, C.: CylView. http://www.cylview.org.
